# Cumulative and different genetic effects contributed to yield heterosis using maternal and paternal backcross populations in Upland cotton

**DOI:** 10.1038/s41598-019-40611-9

**Published:** 2019-03-08

**Authors:** Lingling Ma, Yumei Wang, Babar Ijaz, Jinping Hua

**Affiliations:** 1 Laboratory of Cotton Genetics, Genomics and Breeding/Beijing Key Laboratory of Crop Genetic Improvement/Key Laboratory of Crop Heterosis and Utilization of Ministry of Education, College of Agronomy and Biotechnology, China Agricultural University, Beijing, 100193 China; 2 Institute of Cash Crops, Hubei Academy of Agricultural Sciences, Wuhan, 430064 Hubei China

## Abstract

Heterosis has been utilized in commercial production, but the heterosis mechanism has remained vague. Hybrid cotton is suitable to dissect the heterosis mechanism. In order to explore the genetic basis of heterosis in Upland cotton, we generated paternal and maternal backcross (BC/P and BC/M) populations. Data for yield and yield-component traits were collected over 2 years in three replicated BC/P field trials and four replicated BC/M field trials. At single-locus level, 26 and 27 QTLs were identified in BC/P and BC/M populations, respectively. Six QTLs shared in both BC populations. A total of 27 heterotic loci were detected. Partial dominant and over-dominant QTLs mainly determined yield heterosis in the BC/P and BC/M populations. QTLs for different traits displayed varied genetic effects in two BC populations. Eleven heterotic loci overlapped with QTLs but no common heterotic locus was detected in both BC populations. We resolved the 333 kb (48 genes) and 516 kb (25 genes) physical intervals based on 16 QTL clusters and 35 common QTLs, respectively, in more than one environment or population. We also identified 189 epistatic QTLs and a number of QTL × environment interactions in two BC populations and the corresponding MPH datasets. The results indicated that cumulative effects contributed to yield heterosis in Upland cotton, including epistasis, QTL × environment interaction, additive, partial dominance and over-dominance.

## Introduction

Heterosis refers to the phenomenon of F_1_ hybrids performing better over their parents in yield, quality and adaptation. Dominance, over-dominance and epistasis hypotheses have been proposed to explain the heterosis mechanism. The three hypotheses demonstrated complementarity between dominant alleles and deleterious recessive alleles^1,2^, superiority of heterozygote^3,4^ or mimicry over-dominance with repulsion-phase linkage of favorable alleles^5,6^ and interactions among non-allelic genes^7–9^, respectively. Some previous studies reported the major role of dominance effect on heterosis in rice^10^ and maize^11^. However, over-dominance had also been detected as the primary genetic basis of heterosis for decades, such as in maize^12,13^, rice^14^, rapeseed^15^ and tomato^16^. A *SFT* gene was reported to cause strong yield heterosis governing by over-dominance in plant architecture^17^. The *Dw3* gene contributed to heterosis for plant height in a way of repulsion linkage in sorghum^18^. Additionally, novel experimental design and molecular quantitative genetics approach has been used to elucidate the importance of epistasis at two-locus level in rice during the past decades^19–22^. Recently, Jiang *et al*. suggested that dominance effects played a less prominent role than epistatic effects in grain-yield heterosis in wheat by developing a quantitative genetic framework^23^.

Recombinant inbred line (RIL) population is available to dissect additive and additive × additive effects but lacks heterozygous genotypes to dissect dominance and dominance-related genetic effects. So attempts have been reported by constructing testcross (TC) or backcross (BC) populations and immortalized F_2_ (IF_2_) population to create heterozygotes in rice^10,14,24,25^, maize^11,13,26^ and cotton^27,28^. Dominance complementation was considered as the major genetic basis of heterosis in rice because heterozygotes were superior to respective homozygotes in a BC_1_F_1_ population in rice^10^. Most QTLs underlying grain yield displayed apparent over-dominance effects, and little difference was observed between heterozygous genotypes of nine families of hybrids in three RIL populations in maize^13^. Epistasis and over-dominance were the major genetic bases of inbreeding depression and heterosis for grain and biomass yield by using five rice populations^14^. Heterotic effects and dominance × dominance interaction explained the genetic basis of heterosis in an IF_2_ population deriving from an elite rice hybrid^21^. Over-dominance, pseudo-over-dominance and epistasis were estimated as important contributors to yield heterosis using a high-density genetic map in rice^22^. Among main-effect QTLs and digenic epistatic QTLs pairs, over-dominant loci were the most important than additive, complete and partially dominant loci in two BC populations based on one same RIL population in rice^24^. Dominance, over-dominance and epistasis contributed to the genetic basis of heterosis using a 3,184 bin-map in an IF_2_ population in maize^29^. Moreover, new strategy of heterotic haplotype capture was proposed to trace novel heterozygous chromosome blocks for breeding^30^. A recent report proved that the new statistical models of QTL mapping can completely dissect large-scale time course data in post-genome era^31^.

Yield potential has always been a vital target of plant breeding in cotton. Significant yield heterosis was previously reported in cotton^27^. It is also a major breeding solution to exploit heterosis for improving yield on Upland cotton. For decades, 271 QTLs were available for yield and yield-component traits in the CottonGen database^32^. Among 4268 QTLs in Cotton QTLdb database^33^, 87, 59, 98, 169 and 305 QTLs were detected for seed-cotton yield, lint yield, boll number per plant, boll weight and lint percentage, respectively. However, less QTL have been resolved for seed-cotton yield, lint yield and boll number per plant due to complex experiment management, heavy workload and highly accurate data. The *qSCYchr07a* displayed strong over-dominance effect and the *qSCYchr07c* explained 38.96% of phenotypic variation for seed-cotton yield^34^. A total of 14 QTLs were identified for seed-cotton yield, lint-cotton yield and lint percentage in a RIL population of Upland cotton^35^. Dominance and over-dominance contributed to seed-cotton yield heterosis in an IF_2_ population derived from a heterotic hybrid of ‘XZM 2’ in Upland cotton^36^. Heterotic QTL analysis suggested that over-dominance mainly contributed to cotton yield heterosis^37^. Twenty-three QTLs were identified for boll weight and lint percentage in an intraspecific population of Upland cotton^38^. Fifty-eight QTLs were resolved for three yield-component traits but not for direct yield traits by a linkage map harboring 2618 polymorphic SNP markers^39^. Using two parental BC populations, 58 QTLs were also just mapped for three yield-components in Upland cotton^28^. Therefore, more QTLs controlling yield traits directly need to be identified and the genetic basis for yield heterosis need to be explored in Upland cotton.

In our lab, we have resolved QTL analysis and heterosis for yield and yield-components using F_2_, RIL and maternal backcross (BC/M) populations derived from a commercial hybrid ‘Xinza 1’ in Upland cotton. Partial dominance, over-dominance, epistasis and QTL × environment interaction contributed to yield heterosis in the three populations derived from ‘Xinza 1’^27,40,41^. However, no paternal backcross (BC/P) population had been used to explore the genetic basis of yield heterosis in Upland cotton. Here, we generated a total of 354 BCF_1_ crosses for BC/P and BC/M populations by backcrossing the 177 RI lines to GX1135 and GX100-2, respectively. Backcrossing field trials were carried out including 354 BCF_1_ crosses, the RI lines as current female parents and the common male parent. This experimental design has the obvious advantages: (I) dissecting all genetic components concerning dominance, over-dominance and epistasis effects, and effects by paternal and maternal parents; (II) verifying common even stable QTLs for important traits using three corresponding populations (BC/P, BC/M and RIL) originated from the same hybrid; and (III) generating enough hybrid seeds when needed, similar to IF_2_ population^20^. Seven field trials were performed across two years following a randomized complete block design with two replications. We collected phenotypic data in three corresponding populations for yield and yield-component traits. The study provides new resource to explore the genetic basis of yield heterosis in Upland cotton.

## Results

### Phenotypic performance of parents and populations

Table 1 presents the measurement of yield and yield components over 2015 and 2016. The original female parent GX1135 showed superior performance than the original male parent GX100-2 across multiple environments. We estimated heterosis of the hybrids on average across all environments. Seed-cotton yield (SY) and lint yield (LY) displayed 24.47% and 27.18% mid-parent hybrid vigor on average, respectively, following 10.83% for boll number per plant (BNP), 3.98% for boll weight (BW) and 3.08% for lint percentage (LP) in seven experiments. Mean values were always larger for SY, LY, BNP and LP in BC/M population than in BC/P and RIL populations in both 2015E2 and 2016E2. However, mid-parent heterosis values decreased for a same trait in BC/M population in comparison with that in BC/P population. BNP showed significant and high correlation with SY, as same as with LY (Table 2). We also estimated correlations between measurements of the same trait between the BC/M and BC/P populations in Table 2. The same trait correlated lightly or no significantly between BC/M and BC/P populations. The high correlation showed between RIL-M and RIL-P populations, validating the accuracy of the measurement. The ANOVA analysis indicated that majority of genotype variance were significant at 0.01 or 0.05 probability levels for five traits in BC/P, RIL-P, BC/M and RIL-M populations (Table 3). On the contrary, genotype × environment variance displayed non-significant difference. Heritability of SY decreased from 0.76 in RIL population and 0.64 in BC population to 0.42 in MPH-P dataset in BC/P field trials, similar tendency for majority of traits in BC/P and BC/M trials (Table 3). In addition, significantly positive correlations were observed for yield and yield-components traits between BC and MPH datasets as well as between RIL and BC datasets (Table S1). On the contrary, there was no correlation for five traits between RIL and MPH datasets.

**Table 1:** Descriptive statistical analysis on yield and yield-component traits in BC, MPH and RIL datasets in BC/M and BC/P trials.

Trait^a^	Env.^b^	Mean			Min			Max			CV%^c^		Parents		F_1_	MPH (%)	CK^d^
		BC	MPH(%)	RIL	BC	MPH(%)	RIL	BC	MPH(%)	RIL	BC	RIL	GX1135	GX100-2			
SY/g	2015E1	35.64	5.06	30.81	17.26	−19.91	11.70	63.75	27.07	57.77	25.36	30.14	38.01	37.36	56.36	49.55	58.43
	2015E2 ^†^	60.93	6.54	52.75	12.36	−35.57	21.06	93.08	39.71	89.96	21.96	27.59	57.84	50.86	90.40	36.06	70.88
	2015E3	74.90	8.24	64.70	48.15	−19.47	22.47	100.99	36.97	100.55	12.39	18.59	74.03	71.55	82.57	13.43	80.15
	2016E2	77.15	10.53	63.99	46.80	−11.94	26.65	110.69	42.82	102.51	13.77	22.03	75.78	61.63	75.74	10.25	73.57
	2015E2^†^	59.05	5.89	52.31	25.25	−21.66	19.57	91.51	33.29	96.73	20.63	31.17	47.01	39.05	50.23	16.59	65.40
	2016E1	58.27	8.95	51.27	31.44	−18.10	16.89	87.31	45.41	93.16	21.19	29.47	73.43	47.15	70.15	16.36	78.26
	2016E2	68.13	8.30	61.36	43.95	−16.83	24.40	112.03	36.33	109.20	17.41	22.57	69.66	53.87	79.72	29.07	100.61
LY/g	2015E1	14.66	1.23	10.40	6.66	−7.43	4.28	26.58	13.42	24.35	26.57	16.73	16.25	12.60	21.86	51.59	23.85
	2015E2 ^†^	24.19	3.24	20.82	4.99	−14.41	7.60	38.84	17.46	34.49	22.16	28.43	23.79	17.89	33.57	12.74	27.31
	2015E3	31.00	0.94	21.57	21.28	−8.06	9.27	43.94	17.48	39.52	13.40	10.24	28.23	27.73	32.01	14.40	32.89
	2016E2	32.32	4.84	26.25	19.56	−5.43	11.00	48.75	20.76	44.47	14.74	23.25	34.17	22.36	31.89	12.82	31.58
	2015E2^†^	22.89	3.17	20.21	9.92	−8.98	8.10	34.85	13.06	36.38	20.34	31.05	19.46	15.44	29.52	12.07	18.88
	2016E1	22.46	4.15	19.67	12.37	−7.33	6.25	36.50	20.54	35.55	22.24	29.88	34.92	25.03	39.60	51.36	52.99
	2016E2	27.27	4.25	24.55	17.80	−6.22	11.31	46.11	15.77	43.78	18.42	23.67	28.51	19.23	32.29	35.27	42.56
BNP	2015E1	14.48	1.20	13.26	7.29	−4.20	6.19	17.66	8.76	16.71	18.89	21.64	13.03	11.26	14.35	18.12	14.95
	2015E2	11.12	1.03	10.16	3.20	−5.28	3.84	17.39	8.40	17.69	21.40	27.00	11.75	8.83	12.50	21.49	12.46
	2015E3	21.60	0.74	21.15	16.43	−5.43	12.86	31.00	6.96	28.71	9.37	11.33	21.25	21.18	22.50	6.06	21.21
	2016E2	25.01	1.25	23.32	18.94	−3.25	15.00	35.00	6.88	30.44	9.90	12.63	22.16	21.75	23.22	5.77	20.31
	2015E2	10.76	1.02	9.92	5.73	−4.09	4.00	17.88	7.46	16.50	19.53	29.12	8.38	8.38	11.25	2.88	8.00
	2016E1	24.82	1.81	23.15	18.22	−5.58	13.29	32.81	9.88	32.56	12.03	15.97	22.25	21.75	24.75	15.75	20.75
	2016E2	23.74	1.52	22.56	16.63	−4.84	14.22	29.19	6.63	29.69	10.21	11.90	23.44	20.47	23.22	5.77	20.31
BW/g	2015E1	4.55	1.72	4.18	3.54	−0.91	2.83	5.52	0.99	5.75	7.95	5.55	4.25	4.46	4.78	9.88	5.77
	2015E2	5.47	0.11	5.20	4.40	−1.04	3.99	6.30	1.10	6.25	5.86	8.17	5.53	6.22	6.34	0.47	6.25
	2015E3	4.69	1.56	4.42	4.08	−0.74	3.35	5.63	1.11	5.47	6.12	4.85	5.09	4.59	4.95	2.20	5.03
	2016E2	5.40	0.25	5.03	4.54	−0.33	3.39	6.21	0.89	6.07	5.16	8.43	5.33	5.18	5.37	2.19	6.41
	2015E2	5.49	0.12	5.25	4.41	−0.72	3.53	6.40	0.96	6.45	6.38	9.20	5.66	5.23	6.53	1.08	5.99
	2016E1	4.75	0.17	4.54	3.82	−0.74	3.30	5.95	1.37	6.26	8.42	10.60	5.26	5.56	5.95	9.46	6.12
	2016E2	5.43	0.07	5.13	4.55	−0.69	3.65	6.43	0.84	6.04	5.86	8.08	5.72	5.44	5.73	2.59	6.51
LP/%	2015E1	41.05	1.72	40.73	36.77	−1.30	33.64	45.15	4.68	46.25	3.84	10.01	42.75	33.72	38.80	1.47	40.82
	2015E2	39.71	1.21	39.42	35.13	−2.79	34.21	44.88	4.49	47.25	3.39	5.35	41.13	35.17	39.23	1.08	42.23
	2015E3	41.04	1.56	40.51	36.31	−2.45	35.78	46.49	5.45	45.58	3.61	12.20	38.11	38.82	38.76	0.77	41.07
	2016E2	41.86	0.74	41.02	37.44	−2.98	34.04	45.66	3.99	49.32	3.59	5.77	45.09	36.28	42.10	3.48	42.93
	2015E2	38.81	1.67	38.70	35.04	−1.04	31.35	44.19	4.48	45.68	3.95	5.88	41.03	35.27	40.20	2.04	39.39
	2016E1	38.57	1.75	38.33	33.42	−2.48	31.96	43.61	4.80	45.60	4.57	6.78	42.08	34.46	40.60	6.67	42.63
	2016E2	39.95	1.58	40.05	36.80	−2.38	33.28	44.83	4.96	46.64	39.95	5.73	40.80	35.81	40.63	6.06	42.27

Data with underline indicated the detected QTL in BC/M trial, data without underline indicated the detected QTL in BC/P trial. ^a^SY/g: seed-cotton yield per plant; LY/g: lint yield per plant; BNP: boll number per plant; BW/g: boll weight; LP/%: lint percentage; trait abbreviates. ^b^Environment in 2015 and in 2016, E1: Handan, E2: Cangzhou, E3: Wuhan. ^c^Coefficient of variation. ^d^Competition control of ‘Ruiza 816’ in E1 and E2 and ‘Ezamian 10’ in E3. Hereinafter same. ^†^The values for SY and LY were predicted in 2015E2.

**Table 2:** Correlation analysis between yield and yield-component traits in BC and RIL populations in four BC/M trials and three BC/P trials.

Trait	Env.^a^	SY		LY		BNP		BW		LP	
		BC/M, BC/P	RIL-M, RIL-P	BC/M, BC/P	RIL-M, RIL-P	BC/M, BC/P	RIL-M, RIL-P	BC/M, BC/P	RIL-M, RIL-P	BC/M, BC/P	RIL-M, RIL-P
SY	2015E1/2016E1	0.11 ^b^	0.44								
	2015E2/2015E2	0.16	0.31								
	2016E2/2016E2	0.19	0.45								
LY	2015E1/2016E1	0.98, 0.97^c^	0.96, 0.97	0.10	0.43						
	2015E2/2015E2	0.99, 0.98	0.98, 0.98	0.15	0.31						
	2016E2/2016E2	0.97, 0.98	0.96, 0.96	0.21	0.43						
BNP	2015E1/2016E1	0.82, 0.45	0.72, 0.42	0.75, 0.46	0.70, 0.45	0.20	0.43				
	2015E2/2015E2	0.96, 0.96	0.95, 0.96	0.96, 0.95	0.95, 0.95	0.11	0.36				
	2016E2/2016E2	0.42, 0.47	0.59, 0.48	0.41, 0.48	0.62, 0.48	0.07	0.32				
BW	2015E1/2016E1	0.06, 0.27	0.27, 0.34	0.04, 0.22	0.19, 0.25	−0.01, −0.05	0.01, −0.02	0.16	0.47		
	2015E2/2015E2	0.22, 0.37	0.24, 0.37	0.19, 0.33	0.19, 0.33	−0.05, −0.03	−0.06, 0.11	0.28	0.42		
	2016E2/2016E2	0.10, 0.14	0.38, 0.40	0.03, 0.08	0.29, 0.30	0.01, −0.23	−0.09, −0.08	0.33	0.49		
LP	2015E1/2016E1	0.21, 0.07	0.13, −0.05	0.36, 0.28	0.20, 0.17	0.11, 0.18	0.22, 0.13	−0.17, −0.23	−0.39, −0.36	0.49	0.72
	2015E2/2015E2	−0.01, −0.10	0.08, −0.10	0.15, 0.09	0.26, 0.09	0.03, −0.29	0.15, −0.29	−0.14, −0.02	−0.24, −0.02	0.27	0.62
	2016E2/2016E2	0.11, 0.15	0.04, 0.02	0.35, 0.34	0.28, 0.24	0.07, 0.15	0.20, 0.11	−0.26, −0.29	−0.30, −0.37	0.25	0.42

Critical values of correlation coefficients at probabilities of 0.05 and 0.01 are 0.14 and 0.19, respectively. ^a^Environments are presented as (of BC/M trial)/(of BC/P trial). ^b^Correlation coefficients within underline referred to the measurements of the same trait between BC/M trial and BC/P trial. ^c^Correlation coefficients between two traits in each cell are presented as “(in BC/M trial in the environment), (in BC/P trial in the environment)”.

**Table 3:** ANOVA and heritability of for yield and its components in different populations from both BC/M and BC/P trials.

Trait	Source of variation^a^	BC/M^†^	*H* ^*2£*^	MPH-M	*H* ^*2*^	RIL-M	*H* ^*2*^	BC/P^§^	*H* ^*2*^	MPH-P	*H* ^*2*^	RIL-P	*H* ^*2*^
		MS^b^		MS		MS		MS		MS		MS	
SY	G	593.42**	0.66	170.58	—	593.42**	0.65	460.96**	0.64	271.74**	0.42	834.44**	0.76
	E	84368.39**		1569.58**		116296.78**		10555.01**		1067.42**		10942.12**	
	G × E	226.24**		200.58		206.47		202.81		173.40		253.80*	
	Error	157.96		185.84		204.92		182.90		180.61		216.86	
LY	G	103.29**	0.65	28.81	—	103.29**	0.64	77.26**	0.64	45.15**	0.41	127.27**	0.73
	E	13895.78**		314.74**		18399.46**		2519.36**		81.72		2510.98**	
	G × E	40.03**		33.73		37.20		33.07		27.35		41.91	
	Error	27.41		33.32		37.21		31.74		31.63		36.87	
BNP	G	22.15**	0.53	7.33	—	22.15	—	15.92**	0.32	15.24	—	23.78**	0.44
	E	17120.09**		9.62		10719.36**		21064.51**		60.98**		19182.16**	
	G × E	11.34**		10.84		11.90		10.89		11.86		16.66**	
	Error	9.36		9.82		44.85		12.23		14.46		13.32	
BW	G	1.10**	0.86	0.26**	0.42	1.10**	0.85	0.88**	0.32	0.32**	0.38	0.87**	0.82
	E	81.61**		2.42**		119.95**		48.91**		0.99**		50.00**	
	G × E	0.16*		0.16		0.17*		0.67		0.21		0.20	
	Error	0.14		0.15		0.15		0.64		0.22		0.17	
LP	G	28.95**	0.89	3.55**	0.31	35.50**	0.64	11.48**	0.74	2.73	0.19	30.29**	0.88
	E	165.52**		78.33**		197.38**		210.28**		3.41		254.76**	
	G × E	3.19		2.44		9.68		3.68		2.50		4.75	
	Error	2.86		2.43		18.17		3.39		2.29		4.64	

‘*’ and ‘**’indicated that the correlation was significant at 0.05 and 0.01 probability levels, respectively. The cells with ‘—’ refer to be not suitable to compute heritability due to the estimated method (See part of Method) and the low-accuracy raw datasets. ^a^G, genotype, E, environment, G × E, genotype × environment. ^**†**^Data from 2015E1, 2015E2, 2015E3 and 2016E2. ^§^Data from 2015E2, 2016E1 and 2016E2. ^b^Standard deviation. ^c^Heritibility.

### Single-locus QTLs for yield and yield-component traits

Figure S1 and Table S2 present single-locus QTLs in multiple populations over 2 years by the composite interval mapping (CIM) method. We identified 35 QTLs in BC/M and BC/P populations, 27 heterotic loci by MPH-M and MPH-P datasets, and 41 QTLs in RIL-M and RIL-P populations.

For seed-cotton yield per plant, a total of 10 QTLs were anchored to six chromosomes, respectively. Six common and stable QTLs were identified across multiple environments or in multiple populations. The common *qSY-Chr*2*-1* was simultaneously identified in RIL-P, BC/P and MPH-P datasets over two years. *qSY-Chr*2*-1* explained 27.26% of phenotypic variation in BC/P population in 2016E1 and it was 12.41% in MPH-P dataset. The *qSY-Chr*2*0-*1 was detected in RIL population in two continuous years. Both *qSY-Chr2*1*-1* and *qSY-Chr*2*1-*2 shared between TC and RIL population.

For lint yield per plant, 13 QTLs were identified. They located on nine chromosomes. Six common QTLs explained 5.44–19.61% of phenotypic variation. Four, two and six QTLs were detected in the RIL-M, BC/M and MPH-M datasets, respectively. Six, four, four QTLs were resolved in the RIL-P, BC/P and MPH-P datasets, respectively. *qLY-Chr2-*1 was detected not only in RIL population across three environments but also in the BC/P and MPH-P across three environments. The QTL explained 7.75 to 19.42% of phenotypic variation. In BC/P population, both *qLY-Chr*2*-1* and *qLY-Chr*2*-*2 displayed over-dominant effect in 2016E1 or 2016E2. At the same time, *qLY-Chr*2*-*2 contributed to lint yield heterosis, providing alleles by the female parent among RI lines. Over-dominant *qLY-Chr13-1* was identified in both BC/M and MPH-M datasets. The *qLY-Chr13-1* increased the lint yield with negative additive effect.

For boll number per plant, 23 QTLs were detected on 13 different chromosomes. There are seven common QTLs across multiple environments or datasets. Three QTLs (*qBNP-Chr1-3*, *qBNP-Chr*2*-*2, *and qBNP-Chr14-*2) were repeatedly identified in RIL population across more than one environment. Two common QTLs (*qBNP-Chr15-1*, *qBNP-Chr21-1*) validated each other in RIL and either of two BC populations. And *qBNP-Chr21-2* explained 8.88% and 12.22% of phenotypic variation in BC/M population in 2015E1 and 2016E2, respectively. Two common heterotic loci (*qBNP-Chr1-4* and *qBNP-Chr15-1*) were detected only in BC/P population. *qBNP-Chr1-4* displayed apparent over-dominant effect with *d/a* = 2.60 and *qBNP-Chr15-1* increased one boll number.

Here, nine QTLs were identified for boll weight. Six common QTLs located on chromosome 2, 5, 6, 20 and 23 across more than one environment, respectively. The common QTLs also showed same genetic effect orientation. The stable *qBW-Chr5-2* was identified in RIL population for four times across three locations in two years. It improved boll weight with partial dominance effect in both BC populations. Four heterotic QTLs (*qBW-Chr5-1*, *qBW-Chr5-2*, q*BW-Chr6-1* and *qBW-Chr23-1*) shared in BC/M and BC/P populations. The four QTLs explained 9.54%, 10.73%, 6.19% and 5.54% of phenotypic variation on average, respectively. The common QTL *qBW-Chr20-1* increased boll weight in both RIL and BC/P populations. The QTL provided alleles with negative additive effects.

For lint percentage, seven, six and three QTLs were resolved in RIL-M, BC/M and MPH-M datasets, respectively. Then, seven, five and four QTLs were identified in RIL-P, BC/P and MPH-P datasets, respectively. Three QTLs (*qLP-Chr5-1*, *qLP-Chr5-2* and *qLP-Chr13-3*) verified in RIL, BC/M and BC/P populations at the same time. The *qLP-Chr5-2* was simultaneously detected in the BC/M, BC/P and RIL populations. The *qLP-Chr19-1* and *qLP-Chr13-2* were found only in BC/P population, while *qLP-Chr4-1*, *qLP-Chr7-1* and *qLP-Chr13-2* were observed just in BC/M population. All of the five common QTLs were also detected in RIL population.

Taken together, 71 QTLs were detected for five yield and yield-component traits, including 35 common QTLs (49.30%) in more than one environment or population. A total of 21 QTLs were detected only in BC/M population and 20 QTLs only in BC/P population. Six QTLs were simultaneously detected in both BC/M and BC/P populations. In addition, 12 and 15 heterotic loci were identified using MPH-M and MPH-P datasets, respectively. But there is no common heterotic locus in both MPH datasets. However, 11 common heterotic loci overlapped with seven QTLs in BC/P or RIL-P population and four QTLs in BC/M or RIL-M population (Fig. S1). These overlapping regions distributed on chromosome 1, 2, 5, 6, 7, 13, 15, 21 and 23, respectively.

### Genetic effect at single locus level

Three types of genetic effects were summarized for single locus QTLs in two BC populations and two MPH datasets (Table 4). In BC/M population, 19 (51.35%) additive QTLs and 12 (32.43%) over-dominant QTLs contributed much to heterosis, following six (16.22%) partial dominant QTLs. Homozygous *P2P2* recessive alleles providing by paternal parent changed to be heterozygous *P1P2* alleles in RIL population after crossing to GX1135. In BC/P population, ten (31.25%) additive QTLs and six (18.75%) partial dominant QTLs played slight role in performance than 16 (50.00%) over-dominant QTLs. Homozygous *P1P1* dominant alleles from maternal parent changed to be heterozygous *P1P2* alleles in RIL population after crossing to GX100-2. In BC/M population, there was more over-dominant QTLs for LY, whereas more additive QTLs was resolved for BNP and BW. However, we detected the most over-dominant QTLs for SY and LY in BC/P population. In addition, additive, partial dominant and over-dominant QTLs played important role together for LP in both BC populations.

**Table 4:** Genetic effects of single locus QTLs for yield and yield-component traits in both BC/M and BC/P populations.

Trait	BC/M			Sum	BC/P			Sum
	A^§^	PD	OD		A	PD	OD	
SY	3	0	2	5	0	1	4	5
LY	1	0	6	7	1	0	4	5
BNP	5	1	1	7	3	0	2	5
BW	5	1	0	6	3	1	3	7
LP	5	4	3	12	3	4	3	10
Total	19	6	12	37	10	6	16	32

^§^Three genetic types of a single QTL: A, additive effect, PD, partial dominance effect, OD, over-dominance effect.

### Relationship between whole-genome marker heterozygosity and performance

The experimental design allowed us to dissect relationships between whole-genome marker heterozygosity and trait performance in BC/M, BC/P, MPH-M, and MPH-P datasets. We examined correlations between whole-genome marker heterozygosity of 654 polymorphic loci and one phenotypic dataset for yield and yield-component traits. The majority of correlations showed no significance in all of BC/M, BC/P, MPH-M, or MPH-P datasets (Table S3), demonstrating that overall marker heterozygosity contributed little to yield heterosis. The result was consistent with previous reports^10,20,27^. A previous study demonstrated that a few loci from female parents explained a large proportion of the yield advantage of hybrids but not universally via integrated genomic analyses^42^.

### Pleiotropic region and genetic contribution

Totally, 16 clusters showed pleiotropic effects in 20 cM interval. These clusters involved in 45 QTLs (63.38%) in present study (Fig. S1, Table S4). Cluster-Chr2-1 of SWU11889-SWU11976 was detected in BC/P and MPH-P datasets. The cluster increased 4.30 g seed-cotton yield, 2.26 g lint yield, 1 boll per plant and 0.11 g boll weight. Cluster-Chr5-2 and Cluster-Chr21-1 controlled seed-cotton yield, lint yield and yield-components at the same time.

Six QTL clusters contained QTLs for seed-cotton yield and lint yield at the same time. Importantly, four stable pleiotropic heterotic regions simultaneously controlled seed-cotton yield and lint yield on chromosome (Chr) 2, Chr 5, Chr 11 and Chr 25. Interestingly, all of heterotic loci showed over-dominant effect in the four stable pleiotropic heterotic regions. In BC/P populations, *qSY-Chr2-1* and *qLY-Chr2-1* clustered together and increased yield providing alleles with positive effect value. Three pleiotropic regions increased yield with negative effect value, harboring over-dominant *qSY-Chr5-2* and *qLY-Chr5-1*, *qSY-Chr11-1* and *qLY-Chr11-1*, *qSY-Chr25-1* and *qLY-Chr25-1*, respectively. We searched for physical locations of the flanking markers based on the CottonFGD database^43^. The *qBNP-Chr5-2* in BC/M population and the common QTL *qLP-Chr5-3* in RIL population shared a 333 kb pleiotropic region (Table S4). The pleiotropic region of TMB1296-HAU1603 contained 48 genes on chromosome 5 in Upland cotton (Table S5). Another 516 kb pleiotropic region of NAU2152-NAU5428 contained 25 genes including *qSY-Chr11-1* and *qLY-Chr11-1*. The *qSY-Chr11-1* and *qLY-Chr11-1* showed over-dominant effect.

### Epistasis across multiple environments

We estimated QTLs by inclusive composite interval mapping (ICIM) method in RIL-M, BC/M and MPH-M datasets, respectively. A total of 92, 60 and 35 main effect QTL and QTL × environments interaction (M-QTLs and QEs) were detected for five yield and yield-component traits (Tables 5, S6-A, S6-C, S6-E). The M-QTLs explained 0.41 to 2.47% of phenotypic variation (PV), while the QEs explained 0.14 to 1.49% of PV. At two-locus level, 314, 75 and 36 epistasis QTLs (E-QTLs and QQEs) were identified in RIL-M, BC/M and MPH-M datasets, respectively. The E-QTLs explained 1.06 to 2.93% of PV and QQEs explained 0.58 to 1.48% of PV (Tables 5, S6-B, S6-D, and S6-F).

**Table 5:** Summary on M-QTL and E-QTL by environment interaction for yield and yield components in BC/M and BC/P trials.

Trial	Trait	BC			MPH			RIL		
	M-QTL^a^	n^§^	V(A)%^c^	V(AE)%^d^	n	V(A)%	V(AE)%	n	V(A)%	V(AE)%
BC/M	SY	11	0.72	1.07	7	0.65	1.29	17	1.44	0.63
	LY	14	0.62	1.06	7	0.46	1.40	12	1.64	0.64
	BNP	9	0.53	1.16	6	0.41	1.49	16	0.90	0.81
	BW	13	1.33	0.59	5	0.65	0.96	22	1.98	0.31
	LP	13	1.55	0.71	10	0.88	0.99	25	2.47	0.14
	**Total**	**60**			**35**			**92**		
	**E-QTL** ^**b**^	**n**	**V(AA)%** ^**e**^	**V(AAE)%** ^**f**^	**n**	**V(AA)%**	**V(AAE)%**	**n**	**V(AA)%**	**V(AAE)%**
	SY	13	1.06	1.45	13	0.91	1.78	59	2.31	0.65
	LY	15	1.27	1.48	8	0.87	1.80	52	2.28	0.71
	BNP	8	1.48	1.18	7	0.16	2.78	25	2.31	0.63
	BW	3	2.93	1.21	0	-	-	77	2.75	0.13
	LP	36	1.99	0.58	8	1.48	1.33	101	2.67	0.20
	**Total**	**75**			**36**			**314**		
BC/P	**M-QTL**	**n**	**V(A)%**	**V(AE)%**	**n**	**V(A)%**	**V(AE)%**	**n**	**V(A)%**	**V(AE)%**
	SY	7	2.58	0.50	4	1.44	1.09	10	2.25	0.41
	LY	7	2.89	0.32	3	1.43	1.18	8	2.40	0.34
	BNP	3	1.86	0.49	5	1.37	1.64	9	1.25	1.15
	BW	4	2.35	0.75	5	0.87	1.54	11	2.88	0.23
	LP	13	2.86	0.24	5	0.76	1.83	19	2.75	0.22
	**Total**	**34**			**22**			**57**		
	**E-QTL**	**n**	**V(AA)%**	**V(AAE)%**	**n**	**V(AA)%**	**V(AAE)%**	**n**	**V(AA)%**	**V(AAE)%**
	SY	6	3.09	0.86	2	3.51	0.64	24	3.85	0.32
	LY	6	2.96	0.72	5	3.35	0.46	24	3.72	0.33
	BNP	14	2.39	1.39	6	1.72	1.77	10	2.13	1.78
	BW	10	3.29	0.55	6	2.96	1.38	26	3.96	0.25
	LP	21	2.97	0.21	2	0.65	2.80	31	3.23	0.14
	**Total**	**57**			**21**			**115**		

^a,b^The main effect QTL (M-QTL) and epistasis QTL (E-QTL) by software ICIMapping 4.1. ^§^The number of QTL. ^c,d,e,f^The proportion of phenotypic variation explained on average by single QTL (A), single QTL × environment interaction (AE), epistatic QTLs (AA), and epistatic QTLs × environment interaction (AAE) at the current scanning position, respectively.

A total of 57, 34 and 22 M-QTLs and QEs were detected for five yield and yield-component traits in RIL-P, BC/P and MPH-P datasets across three environments, respectively (Tables 5, S7-A, S7-C, S7-E). On average, the M-QTLs explained 1.86% to 2.89% of PV while the QEs explained 0.24% to 0.75% of PV. At two-locus level, we detected 115, 57 and 21 E-QTLs and QQEs in RIL-P, BC/P and MPH-P datasets, respectively. The E-QTLs explained 0.65 to 3.96% of PV and QQEs explained 0.14% to 2.80% of PV (Tables 5, S7-B, S7-D, and S7-F).

Three genetic types of epistasis were resolved for yield and yield-component traits in RIL-M, RIL-P, BC/M, BC/P, MPH-M and MPH-P datasets (Table S8): Type I, interaction between two M-QTLs; Type II, interaction between one M-QTL and non M-QTL; Type III, interaction between one non M-QTL and another non M-QTL^27^. The number of E-QTLs with Type III was 241, 59, 27 in RIL-M, BC/M and MPH-M datasets, respectively; that with Type II is 70, 14, 8; that with Type I is 3, 2, 1. The numbers of E-QTLs were 2, 0, 0 for Type I, respectively; 17, 4, 4 for Type II; 93, 57, 13 for Type III. The result demonstrated that large number of E-QTLs contributed to phenotype in RIL and BC populations and heterosis between numerous non M-QTLs, which displayed minor effect together.

## Discussions

### Experimental design for heterotic loci by multiple mapping populations

The extended design of NCIII was suitable to explore heterosis by creating heterozygotes by backcrossing or testcrossing parental lines to RIL or doubled  haploid (DH) lines^25,27,28^. In the present study, we constructed two parental BC populations based on one RIL population as the permanent experimental design. Superior phenotypic values displayed in BC/M population rather than that in BC/P population, suggesting that the performance of both parents determined the performance of their hybrid. In all of seven field trials, every backcrossing progeny inter-planted in the middle of both parents. The experimental design allowed calculating the mid-parent heterosis (MPH) so as to detect heterotic loci for measuring heterotic effect directly^24^. Hua *et al*. separated 33 heterotic loci that caused yield heterosis in rice^21^. Here, 27 heterotic loci were resolved from two parental BC populations (Table S2). Two stable heterotic loci (*qSY-Chr2-1* and *qLY-Chr2-1*) shared the region of SWU11889-SWU11950. The *qSY-Chr2-1* increased 2.61 g – 8.72 g seed-cotton yield across four environments (Table S9). At the same time, *qSY-Chr2-1* explained 12.85% and 27.26% of phenotypic variation in MPH-P and BC/P datasets, respectively. The *qLY-Chr2-1* increased 1.36 g- 4.11 g lint yield across six environments in 2015, 2016 and previous 2012^27,41^ (Table S9). In addition, the QTL explained 12.41% and 19.61% of phenotypic variation in the MPH-P and BC/P datasets, respectively. Both *qSY-Chr2-1* and *qLY-Chr2-1* displayed apparent over-dominance effect (OD) with the degree of dominance of *d/a* ranged from 1.6 to 4.9 in BC/P population. Three other common and clustering heterotic loci contributed to yield heterosis by the same genetic mode (Fig. S1, Table 5). These heterotic loci shared the region controlling SY and LY on chromosome 5, 11 and 25, respectively. Interestingly, 11 heterotic loci (64.71%) mentioned above overlapped with the QTLs which were detected in both BC/M and BC/P populations for yield-component traits, including *qSY-Chr2-1*, *qLY-Chr2-1*, *qLY-Chr2-2*, *qLY-Chr2-3*, *qLY-Chr13-1*, *qBNP-Chr1-4*, *qBW-Chr5-1*, *qBW-Chr23-1*, *qLP-Chr5-2*, *qLP-Chr7-1 and qLP-Chr13-3*. The results implied that some heterotic loci linked with QTLs together among five yield and yield-component traits. However, only two common heterotic loci (2.82%) were identified across multiple environments or populations in present study, including *qSY-Chr2-1* and *qLY-Chr2-1*. The result assumed that each measurement depended on the neighboring materials in one plot because of the sensitivity to environment for yield heterosis and the apparent marginal effect of Upland cotton plant.

### Common QTLs and their genetic effects at single locus level

In present study, 35 common QTLs (49.30%) were identified in more than one environment or population by using RIL, BC/M, BC/P, MPH-M and MPH-P datasets. In previous study, 58 common QTLs were identified using RIL and BC/M populations^27,41^. Among these common QTLs, 17 major QTLs explained over 10.00% of phenotypic variation. A total of 19 QTLs in present study were same to previous QTLs in three BC/M trials in 2012^27^ (Table S9). Totally, 9 common previous QTLs were identified in the F_2_ populations in 2008 and 2009^40^ (Table S10). Three QTLs of Cluster-Chr5-1 increased boll weight and lint percentage at the same time over F_2_, RIL, BC/M and BC/P populations, including *qBW-Chr5-1*, *qBW-Chr5-2* and *qLP-Chr5-1* (Tables S4, S9, S10). Taken together, a total of nine common QTLs validated across multiple years of 2012, 2015 and 2016 for seed-cotton yield and lint yield traits. The region of SWU20917-NAU6240 explained 10.78–37.72% of phenotypic variation across multiple environments in RIL and BC populations. All of QTLs flanking with SWU11887 increased phenotypic performance of SY, LY and BW on chromosome 2 (Table S4). In this study, the BNL1317 flanking *qSY-Chr9-1* for seed-cotton yield was common to the previous QTL with LOD 4.94 controlling lint percentage^35^. In present study, three markers of Gh157, BNL1495 and CGR5390 involved in *qBNP-Chr13-1*, *qLP-Chr13-2* and *qLP-Chr13-3* for boll number per plant and lint percentage on chromosome 13. The three markers were common to the previous markers for lint percentage^35^, the previous QTL (*qLY-Chr13-1*) for lint yield^40^ and previous association locus *qLP-D5-1* for lint percentage^44^. There are 150 same SSR markers between our linkage map in present study and the previous map including 2051 SSR loci^38^. Two QTLs (*qLY-Chr21-3*, *qBNP-Chr21-3*) involving in BNL3442a increased lint yield and boll number per plant in our study, similar in another previous report^36^. The common QTLs and validated QTLs across multiple environments and multiple populations provide a valuable resource for MAS and the further research. The results indicated that the design in present study was efficient to map common even stable QTLs or heterotic loci across multiple populations. In addition, we observed 48 genes in a 333 kb region (TMB1296-HAU1603) in the reference genome. And the 516 kb pleiotropic region (NAU2152-NAU5428) contained 25 genes in the reference genome (Table S5). In further study, we will focus on the two regions for fine mapping and gene function analysis. The availability of cotton genomic data for diploid species^45–47^, tetraploid genomes^48–51^ facilitated the development of single nucleotide polymorphism (SNP) markers. Until now, 302,735 SNPs were deposited in CottonGen database^32^. The development of CottonSNP63K array and high-throughput genotyping arrays facilitate applications of SNP markers to linkage mapping and GWAS in cotton^39,42–48^.

### Genetic basis on heterosis in Upland cotton

At single locus level, 19 (51.35%), 6 (16.22%), 12 (32.43%) QTLs were estimated in BC/M population for additive effect, partial dominance effect and over-dominance effect, respectively (Table 4). In BC/P population, the number of QTLs were 10 (31.25%) for additive effect, 6 (18.75%) for partial dominance effect and 16 (50.00%) for over-dominance effect, respectively. The result indicated that three types of genetic effects were detected at the single-locus level in BC/M population, similarly in BC/P population. However, the most QTLs showed additive effect, following over-dominant effect in BC/M population. The result was consistent with a null hypothesis that gene expression will be additive in the hybrid in comparison with their expression in the parents^52^. However, the most QTLs showed over-dominant effect in BC/P population. For yield and yield-component traits, additive effect is the most important in hybrids by crossing RI lines to superior performance parent harboring dominant alleles, whereas partial dominant and over-dominant effects are major genetic basis in hybrids by crossing RI lines to inferior performance parent harboring recessive alleles.

Epistasis refers to the interaction between alleles from different loci^7^. In present study, 75, 36, 57 and 21 epistatic QTLs (E-QTLs and QQEs) were identified in BC/M, MPH-M, BC/P and MPH-P datasets, respectively (Tables 5 and S6, S7). The result indicated that epistasis contributes to heterosis in consistent with previous studies^19–22^. Moreover, E-QTLs and QQE explained higher phenotypic variation (PV) than that by main effect QTL by environments (M-QTL and QEs) in both BC/M and BC/P populations. The QTLs for same trait explained more portion of PV in BC/P and MPH-P datasets than that in the BC/M and MPH-M datasets. On the contrary, QTL × environments interaction (QE or QQE) explained less PV in BC/P and MPH-P datasets than that in BC-M and MPH-M datasets. The results indicated that E-QTLs played role in yield heterosis in ‘Xinza 1’. Environment only explained 0.13–2.80% of PV at two-locus level, suggesting that yield heterosis was sensitive to environment in Upland cotton (Table 5). In a short, we detected additive, partial dominance, over-dominance at single locus level together with epistasis and environment interactions. The results indicated that cumulative effects controlled yield heterosis in Upland cotton, consistently with the previous results^27,40^. Guo *et al*. reported the contribution of over-dominant QTLs for heterosis by using an interspecific cotton population^37^. Similarly, genetic basis of grain yield heterosis were the cumulative effects of dominance, over-dominance, and epistasis in maize hybrid ‘Yuyu 22’^29^. Recently, additive, partial dominance and over-dominance controlled heterosis owing to allelic dosage effects in maize^53,54^ and rice^42^. The genome-wide heterozygosity of hybrids made a limited contribution in present study. The result was in consistent with previous report for biomass heterosis by characterizing the genomic architecture in 200 *Arabidopsis* hybrids^55^. As other polyploidy plants, cotton also exhibits better vigor after polyploidization event^52^. The yield and its component traits are complex quantitative traits. The genetic basis of heterosis is mysterious especially the allotetraploid Upland cotton. Pleitropic regions involving in heterotic loci contained numbers of genes. The regions with cumulative genetic effects maybe regulate yield heterosis in a particular inheritance mode such as dosage effects. Further work need to explore heterosis mechanism using one single and novel gene in Upland cotton.

## Methods

### Development of the experimental populations

Recombinant inbred line (RIL) population were previous developed by single seed descent method, which derived from the Upland cotton hybrid ‘Xinza 1’ (GX1135 × GX100-2)^27,40^. The 177 F_14_ individuals of RIL population were re-planted for inbred seeds. A total of 354 progenies were generated by backcrossing 177 RI lines to GX1135 (as the present common male parent) and GX100-2 (as the present common male parent), respectively. We named the maternal and paternal backcross populations as BC/M and BC/P populations for short, respectively, the same as RIL-M population and MPH-M dataset in BC/M field trials, and RIL-P population and MPH-P dataset in BC/P field trials (See below).

### Field design and management

Two kinds of backcross trials were carried out as follows: (I) the paternal BC trial (BC/P field trial), containing BC/P population by cross of 177 F_14_ RILs × GX100-2, RIL-P population and common male parent GX100-2 (original male parent); (II) the maternal BC field trial (BC/M trial), containing BC/M population by cross of 177 F_14_ RILs × GX1135, RIL-M population and common male parent GX1135 (original female parent). We carried out seven field trials over two years of 2015 and 2016 at three locations in China as follows: E1, Handan, Hebei Province; E2, Cangzhou, Hebei Province; E3, Wuhan, Hubei Province^56^. Four BC/M trials were performed in 2015E1, 2015E2, 2015E3 and 2016E2 (the year and location). Three BC/P field trials were constructed in 2015E2, 2016E1, and 2016E2. All of seven field trials followed a randomized complete block design with two replications. The control set was planted in seven field trials, respectively, including GX1135, ‘Xinza 1’ F_1_, GX100-2 and a competition control of Upland cotton hybrid variety (‘Ruiza 816’ or ‘Ezamian 1’)^56^. Unfortunately, three field trials encountered hailstone disaster on June 11, 2015 in E2 and on June 28, 2016 in E1. After the hailstone disasters, we immediately performed effective field managements to recover the damaged plants (2015E2 for one BC/P trial and one BC/M trial, 2016E3 for one BC/P trial). Therefore, we regarded as the experiments in a same identical and natural environment because of the well recovery of the plants (Fig. S2). The details for three field trials at E1 and E2 in 2016 are same to the arrangement in 2015 in the previous report^56^. The field management followed the conventional standard field practices.

### Phenotypic evaluation

We scored eight plants except the marginal one for phenotypic performance. We harvested seed cotton in each plot for seed-cotton yield per plant (SY) and boll number per plant (BNP) at maturity stage. Twenty-five naturally opening bolls were randomly hand-harvested in the middle of plants for boll weight (BW) and lint percentage (LP). We evaluated lint yield per plant (LY) by multiplying SY by LP. In addition, SY in 2015E2 was predicted by multiplying BNP and BW due to unfavorable hailstone disasters. A total of 45,174 plants were measured for yield and yield-component traits at three locations across two testing years. At last, we collected seven complete datasets from four BC/M trials and three BC/P trials for five yield and yield-component traits.

### The genetic linkage map information

A total of 623 polymorphic SSR loci were previously classified into 31 linkage groups anchored on 26 chromosomes^27^. The genetic map covered 3889.9 cM (88.20%) with interval of 6.2 cM on average. The genotypes of 177 individuals in the RIL population were reported previously, as well as that of the BC/M population^27^. The genotypic data of the BC/P population were deduced from that of RIL population (Table S11).

### Statistical analysis

Data for five yield and yield-component traits were obtained over 2 years from four populations (the RIL-M, BC/M, RIL-P, BC/P population) and the currently common male parents. Basic statistical analysis was performed by the software SPSS (version 19.0, SPSS, Chicago). Two mid-parents heterosis datasets (MPH-M and MPH-P datasets) were assessed by the equation:

$${\rm{MPH}}={{\rm{F}}}_{1}-{\rm{MP}},\,{\rm{MP}}=({{\rm{P}}}_{1}+{{\rm{P}}}_{2})/2,$$

where F_1_ refers to phenotype value of each BC_1_F_1_ progeny in BC/M or BC/P populations_;_ P_1_ refers to the recurrent female parent of the RIL-M or RIL-P populations; P_2_ refers to the currently male donor parent of GX1135 in BC/M trial or GX100-2 in BC/P trial. Heterosis (%) was assessed by the equation^30^

$${\rm{MPH}}( \% )=({{\rm{F}}}_{1}-{\rm{MP}})/{\rm{MP}}\times 100 \% .$$

The dataset of mean value for two replications were used to map quantitative trait locus (QTL) in every single environment for five yield-related traits. We estimated variance for multiple datasets in multiple environments for five yield-related traits by R software. The linear model formula was as

$${y}={G}+{E}+G\times E+block+error,$$

where *G* refers to genotype effect, *E* to environment effect, *G* × *E* to genotype-by-environment interaction effect, *block* to repeat effects in one environment, *error* to error effect. Based on the variance component, heritability was calculated in the equation as

$${h}^{2}={{\delta }^{2}}_{G}/[{{\delta }^{2}}_{G}+({{\delta }^{2}}_{G\times E})/env+{{\delta }^{2}}_{e}/(env\times rep)],$$

where *δ*^*2*^_*G*_, *δ*^*2*^_*G×E*_, and *δ*^*2*^_*e*_ refer to the genotypic variance, genotype-by-environment interaction variance, and error variance, *env* to the number of the environments, and *rep* to the number of replications per environment^57^. For the low-accuracy raw datasets, larger error variance was not allowed to estimate heritability due to the environment sensitivity and/or bigger artificial error. We used QTL Cartographer (Version 2.5)^58^ to map single-locus QTL by composite interval mapping (CIM) method. The genetic effect values were estimated in the confidence interval of 95%. The threshold of LOD values were estimated after 1000 permutations tests to declare a significant QTL with a significant level of *P* < 0.05. However, a common QTL was considered with LOD 2.0 in another environment or population^27,39^. Common QTLs were evaluated by linked position and shared common markers^59^. Stable QTLs in the present study referred to common QTLs with stable genetic effect orientation in multiple environments and/or populations. The degree of dominance was estimated for common QTLs derived from different populations or datasets^15^. Genetic effects of single-locus QTLs were defined as: additive effect loci just detected in BC populations, the complete or partial dominance effect loci with *d*/*a* ≤ 1, over-dominance effect loci with *d*/*a* > 1 or QTLs detected by MPH dataset^25,60^. The genetic effects of single QTL were assessed following: additive effect,

$$a=({P}_{1}{{P}_{1}}^{RIL}-{P}_{2}{{P}_{2}}^{RIL})/2,$$

where *P*_*1*_*P*_*1*_ and *P*_*2*_*P*_*2*_ stands for effects of homozygous genotypes from RIL population; dominance effect,

$$d=({P}_{1}{{P}_{2}}^{He}-{P}_{2}{{P}_{2}}^{He}),$$

where *P*_*1*_*P*_*2*_ and *P*_*2*_*P*_*2*_ stand for effects of heterozygous genotypes after correction of BC_1_F_14_ observation for mid-parent performance (MPH);

$${P}_{1}{{P}_{2}}^{BC1F14}-\,{P}_{2}{{P}_{2}}^{BC1F14}=a+d,$$

for BC/M and BC/P populations^25,27^. The software IciMapping 4.1 (www.isbreeding.net) was used to test additive, dominance and epistasis under environments by inclusive composite interval mapping (ICIM) method. The main-effect QTL (M-QTL) and its environmental interaction (QTL × environment, QE), epistatic QTLs (E-QTLs) and its environmental interactions (QTLs × environment, QQE) were conducted using RIL, BC and MPH datasets under multiple environments in two parental BC trials. A threshold LOD 2.5 and 5 scores were used to declare significant M-QTL and E-QTLs, respectively^27,61–66^.

## Supplementary information

Supplementary Information

Dataset 1

Dataset 2

## Data Availability

The authors state that all data necessary for confirming the conclusions presented in the article represented fully within the article and in the Table S11.
