# Supplementary material for: Cumulative and different genetic effects contributed to yield heterosis using maternal and paternal backcross populations in Upland cotton
Source: Sci Rep. 2019 Mar 8;9:3984. doi: 10.1038/s41598-019-40611-9 (PMC6408543; doi:10.1038/s41598-019-40611-9)
Supplement: Supplementary file 1 — Supplementary Information [file 41598_2019_40611_MOESM1_ESM.docx]

**Cumulative and different genetic effects contributed to yield heterosis using maternal and paternal backcross populations in Upland cotton**

**Lingling Ma ^1^, Yumei Wang ^2^, Babar Ijaz ^1^, Jinping Hua ^1, *^**

**^1^** Laboratory of Cotton Genetics, Genomics and Breeding /Beijing Key Laboratory of Crop Genetic Improvement /Key Laboratory of Crop Heterosis and Utilization of Ministry of Education, College of Agronomy and Biotechnology, China Agricultural University, Beijing 100193, China

^2^ Institute of Cash Crops, Hubei Academy of Agricultural Sciences, Wuhan 430064, Hubei, China

^*^ Corresponding author: College of Agronomy and Biotechnology, China Agricultural University, Beijing 100193, China. E-mail: jinping_hua@cau.edu.cn

E-mail addresses of all authors:

[LLinging00@cau.edu.cn](mailto:LLinging00@cau.edu.cn.) (Lingling Ma)

[yumeiwang001@126.com](mailto:yumeiwang001@126.com) (Yumei Wang)

babar_ijaz@cau.edu.cn (Babar Ijaz)

[jinping_hua@cau.edu.cn](mailto:jinping_hua@cau.edu.cn) (Jinping Hua)

**Supplementary Figure S1. Locations of QTLs controlling yield and yield-component traits identified in BC, MPH and RIL datasets in BC/M and BC/P trials.** * and ** (# and ##), marker showed respectively segregation distortion significant at P = 0.05 and 0.01 levels; markers with * and ** skewed toward the GX1135 alleles, and markers with # and ## skewed toward the GX100-2 alleles. SY: seed-cotton yield per plant; LY: lint yield per plant; BNP: boll number per plant; BW: boll weight; LP: lint percentage; trait abbreviates. Two black boxes focused the important regions we analyzed.

SY LY BNP BW LP

BC

MPH


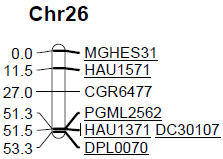

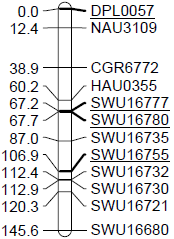


**XZV**

RIL


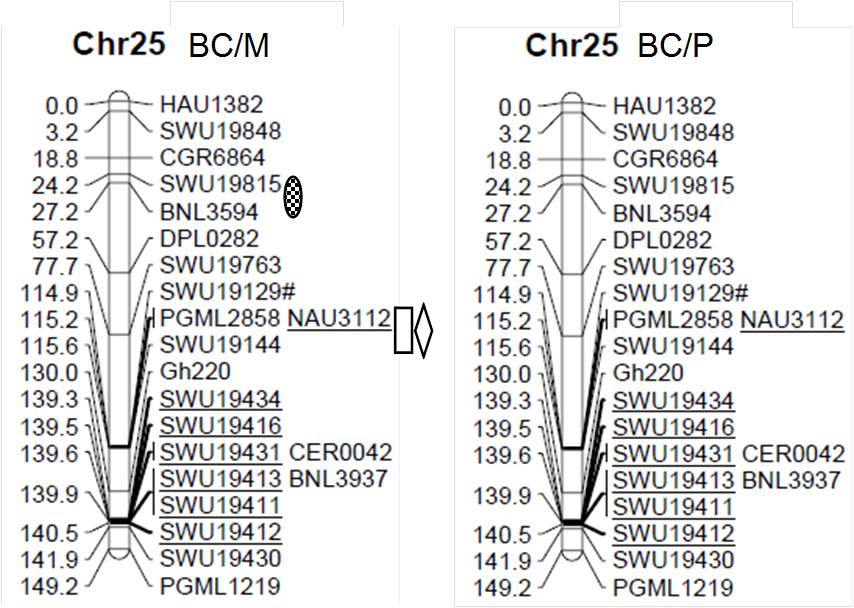


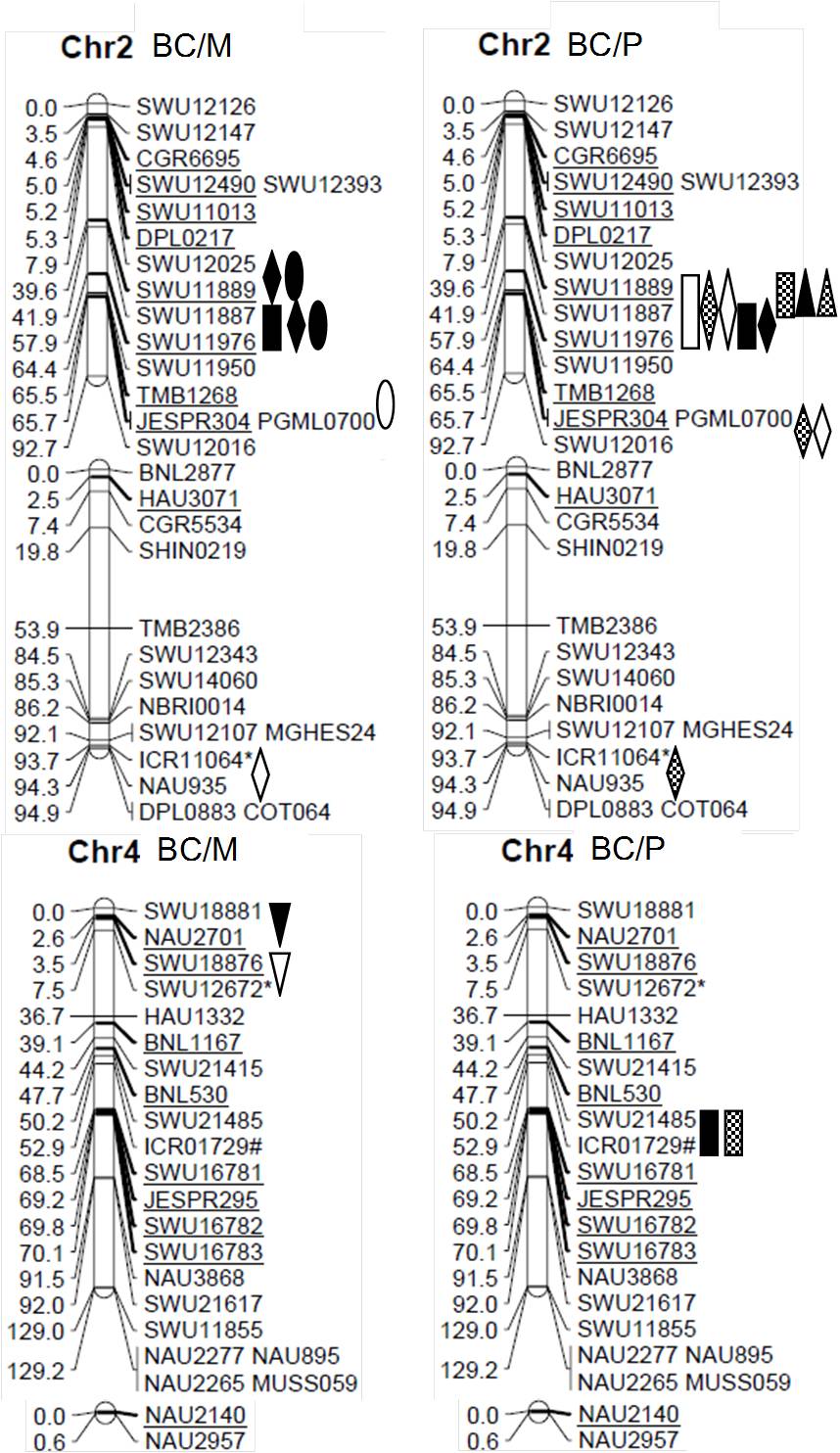

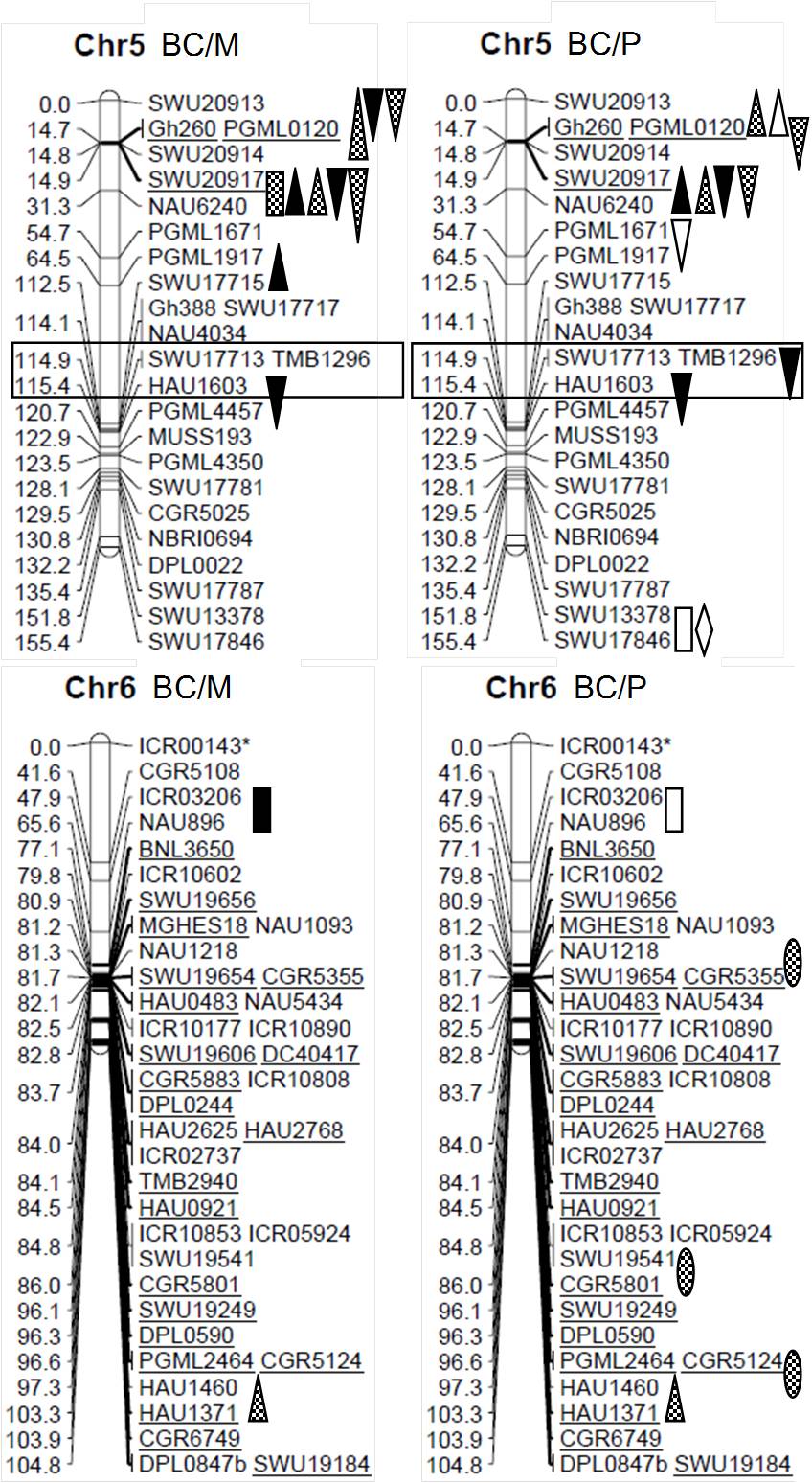

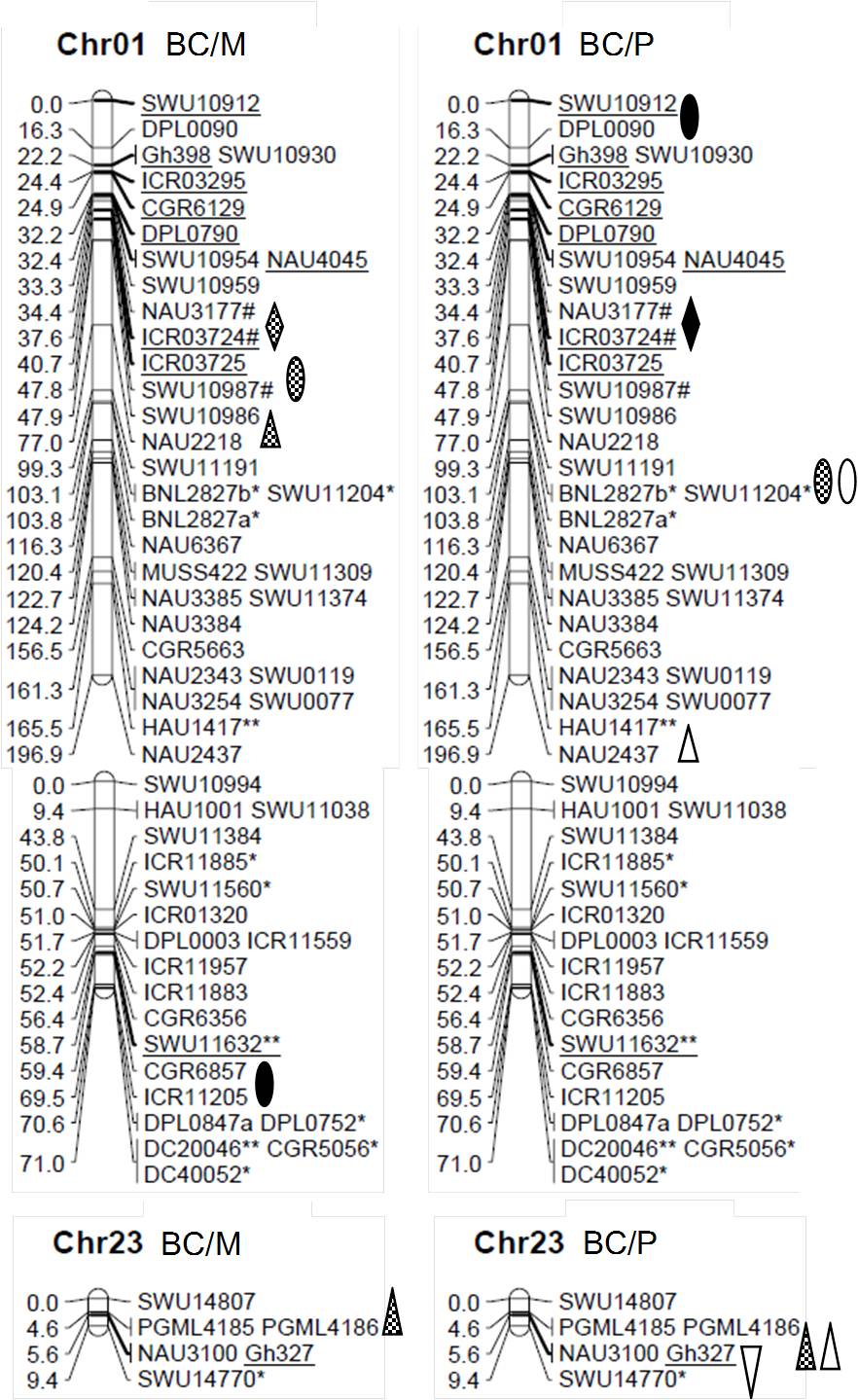


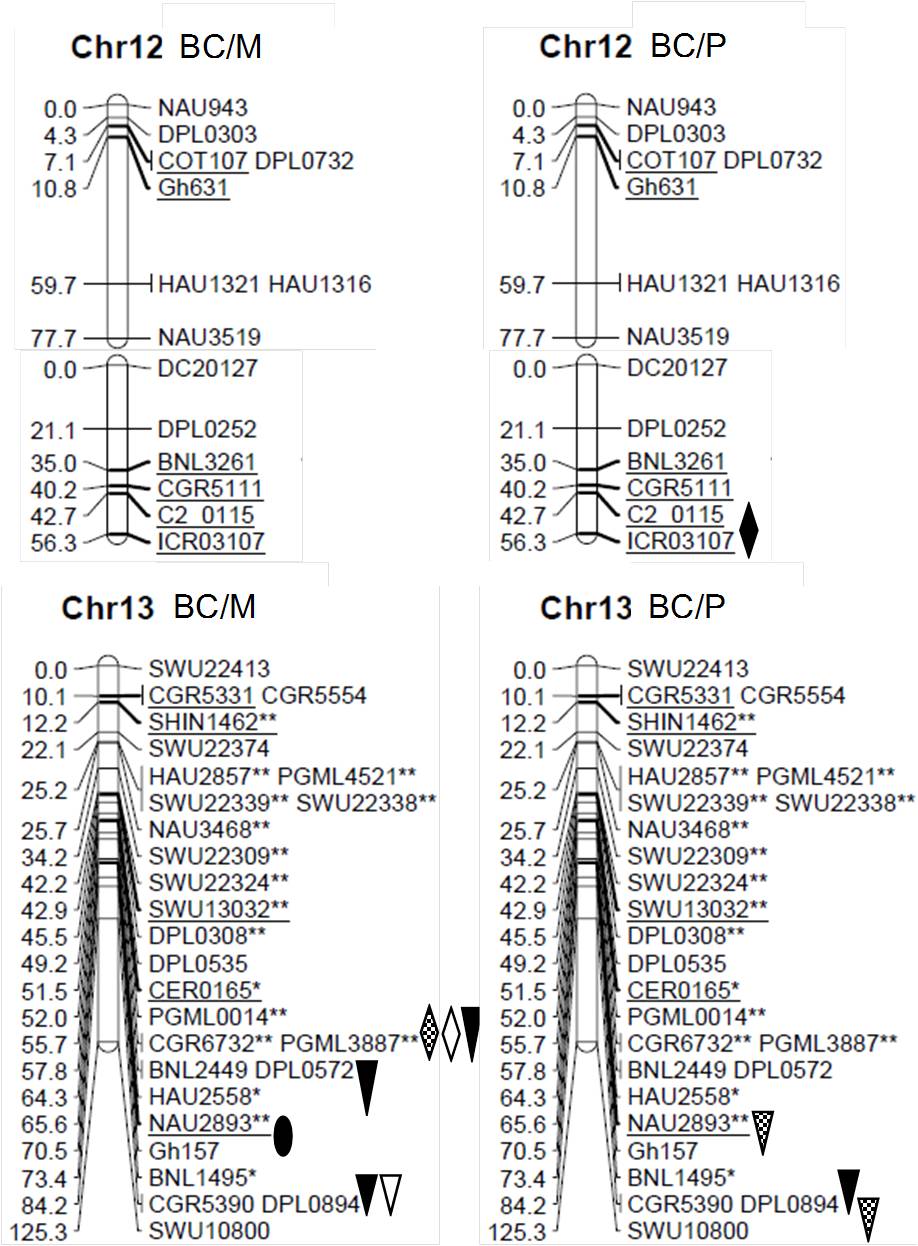

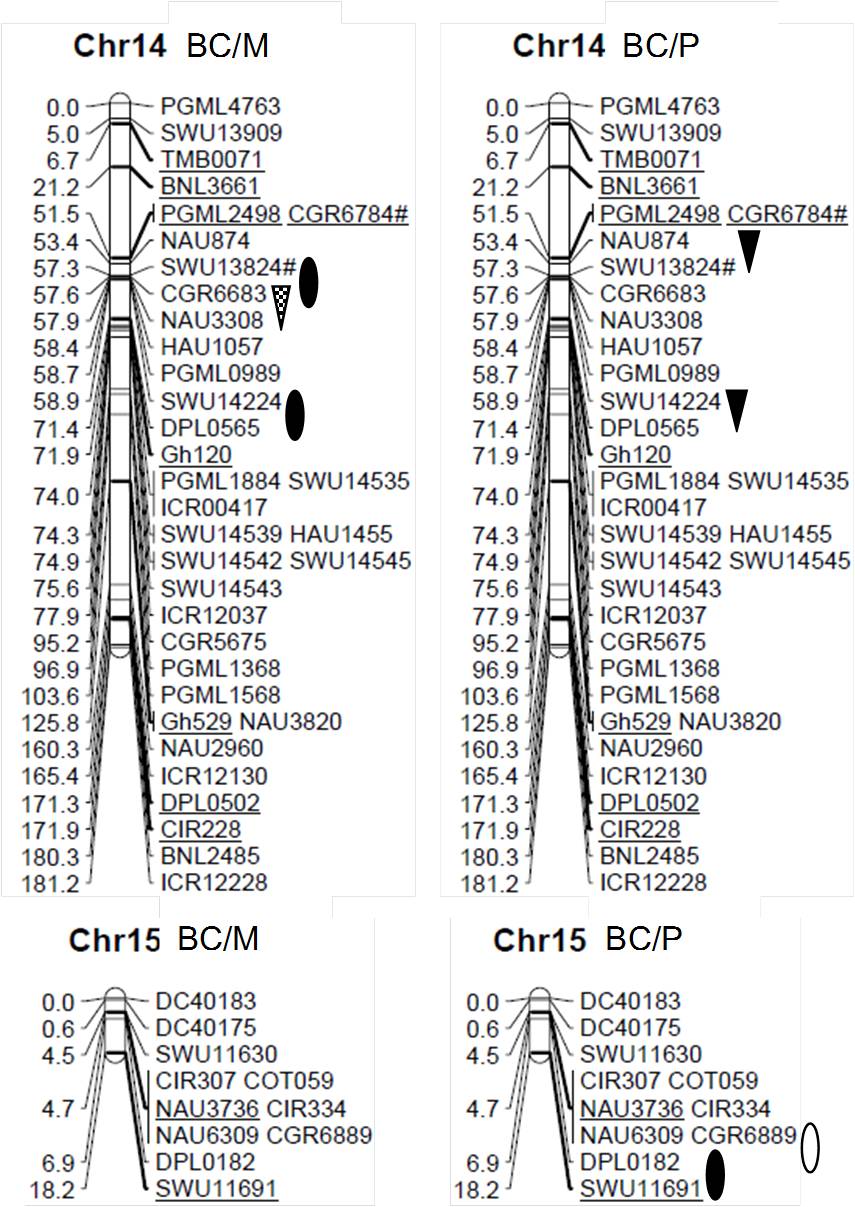

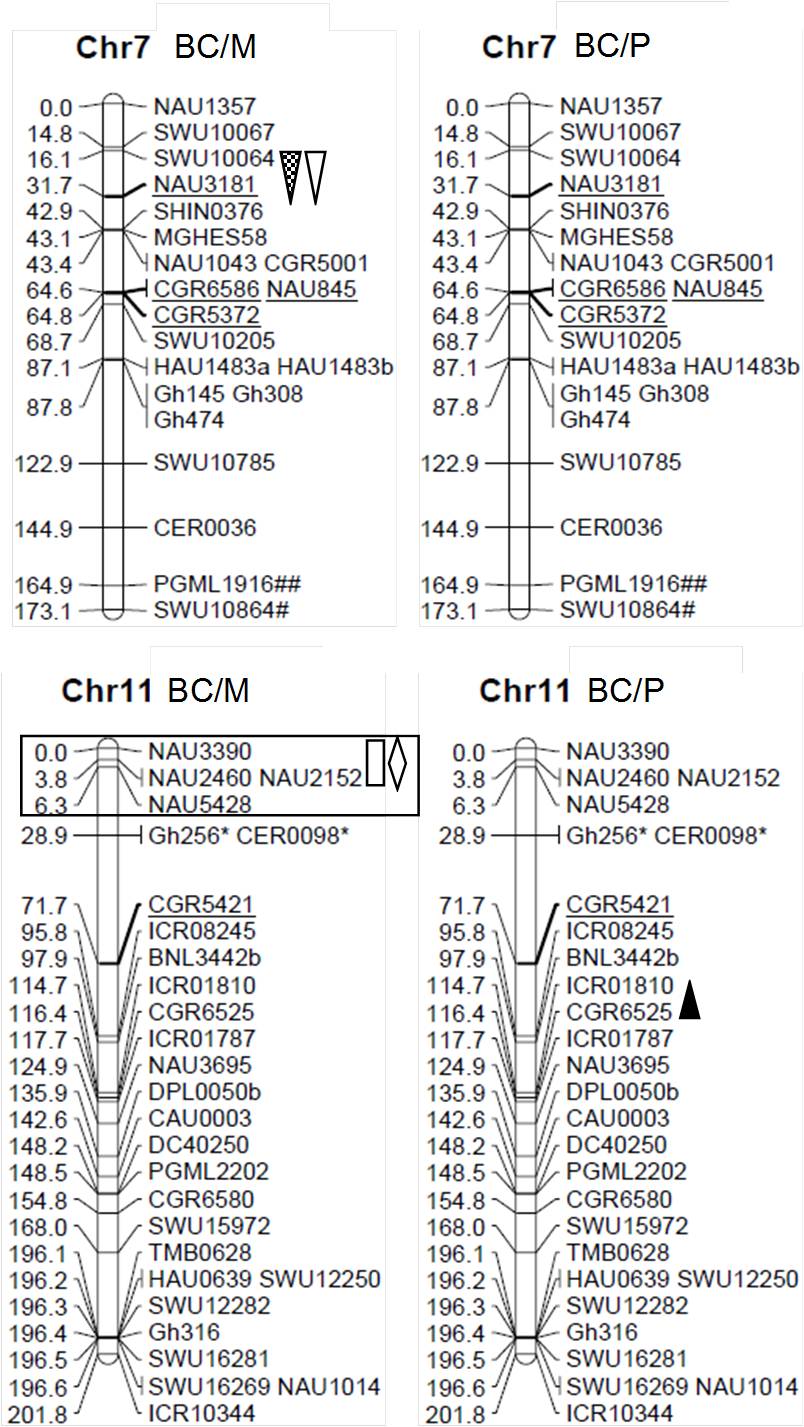


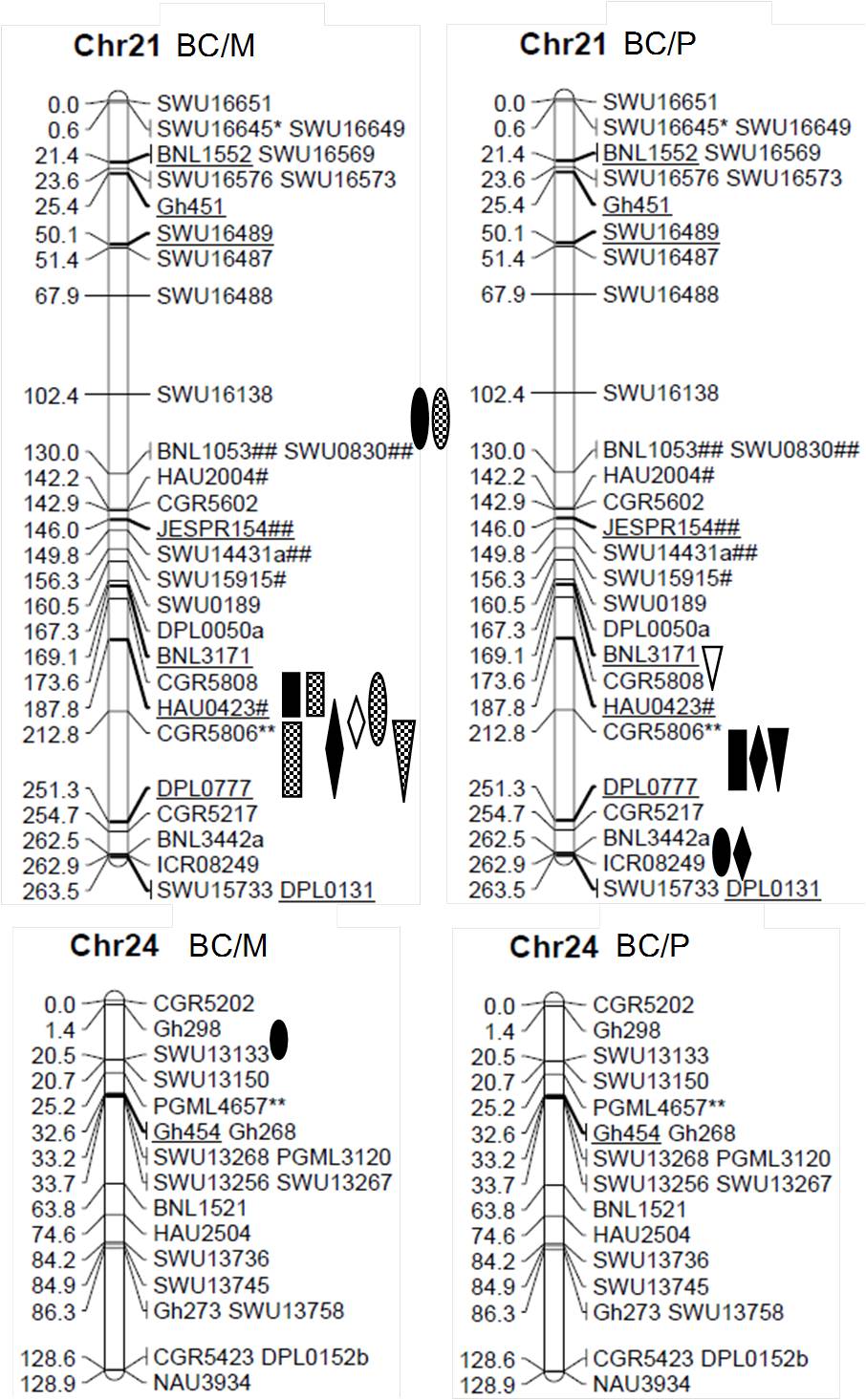

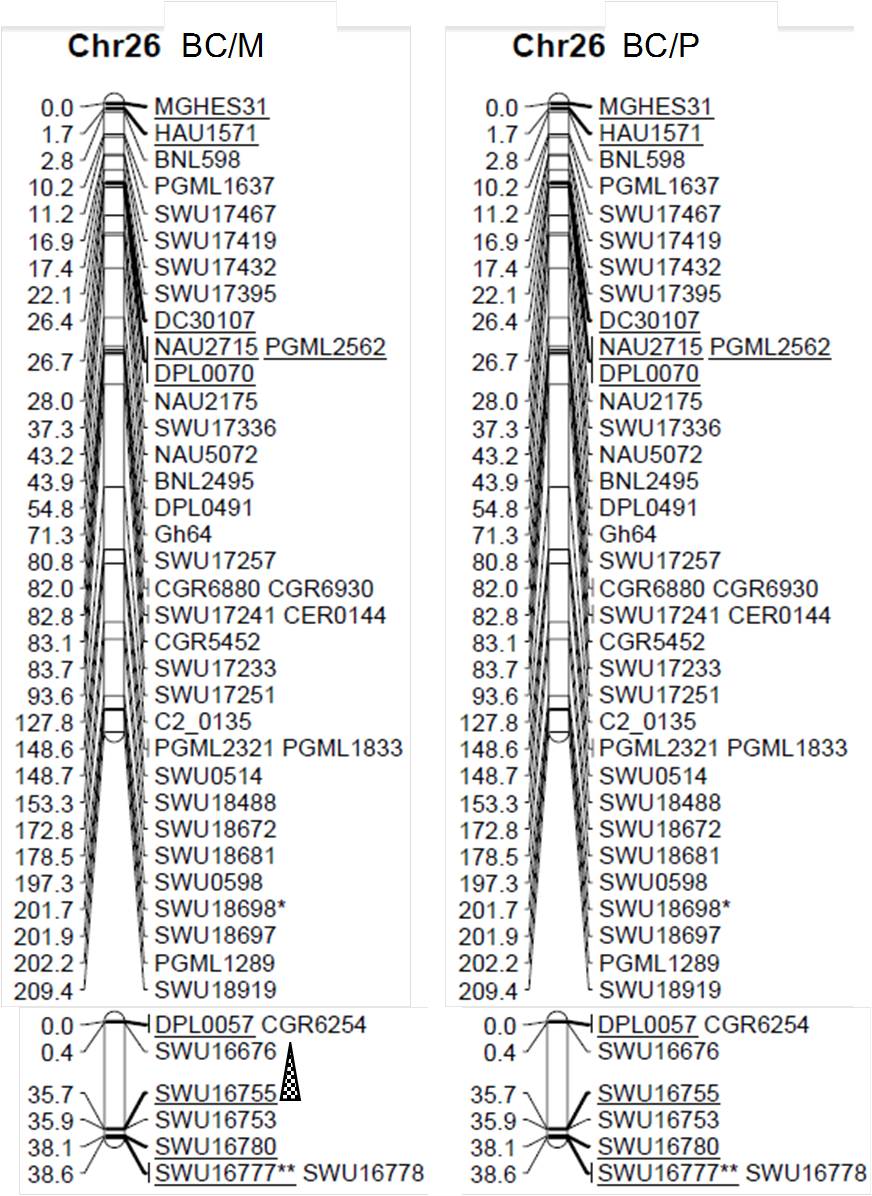

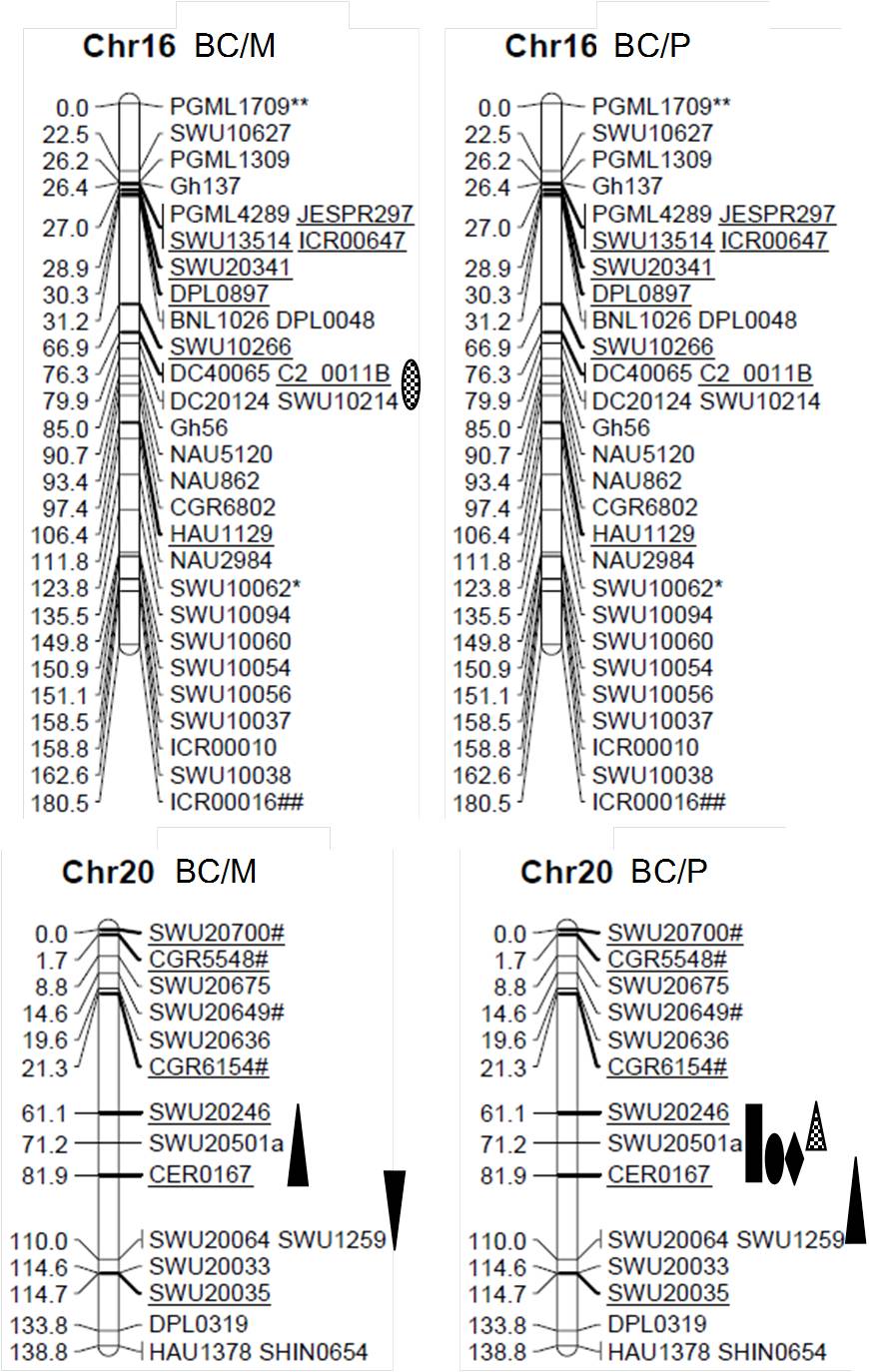


**Supplementary Figure S2. Plants in the BC/P trial encountered hailstone in 2016E1 (Handan City, Hebei Province).** A. Plants for 2 days after hailstone disaster (AHD) on June 30^th^, 2016. B. Plants for 4 days AHD with recovered cultivation of fertilization and loosening the soil on July 2^ed^, 2016. C. The recovery plants for 12 days AHD on July 10^th^, 2016. D. Plants for 62 days AHD on August 30^th^, 2016. E, F. The recovery plants without apical bud (E) and with apical bud (F) for 35 days AHD on August 3^th^, 2016. G. The overall plants of the trial in 2015E1 for 62 days AHD on August 30^th^, 2016.


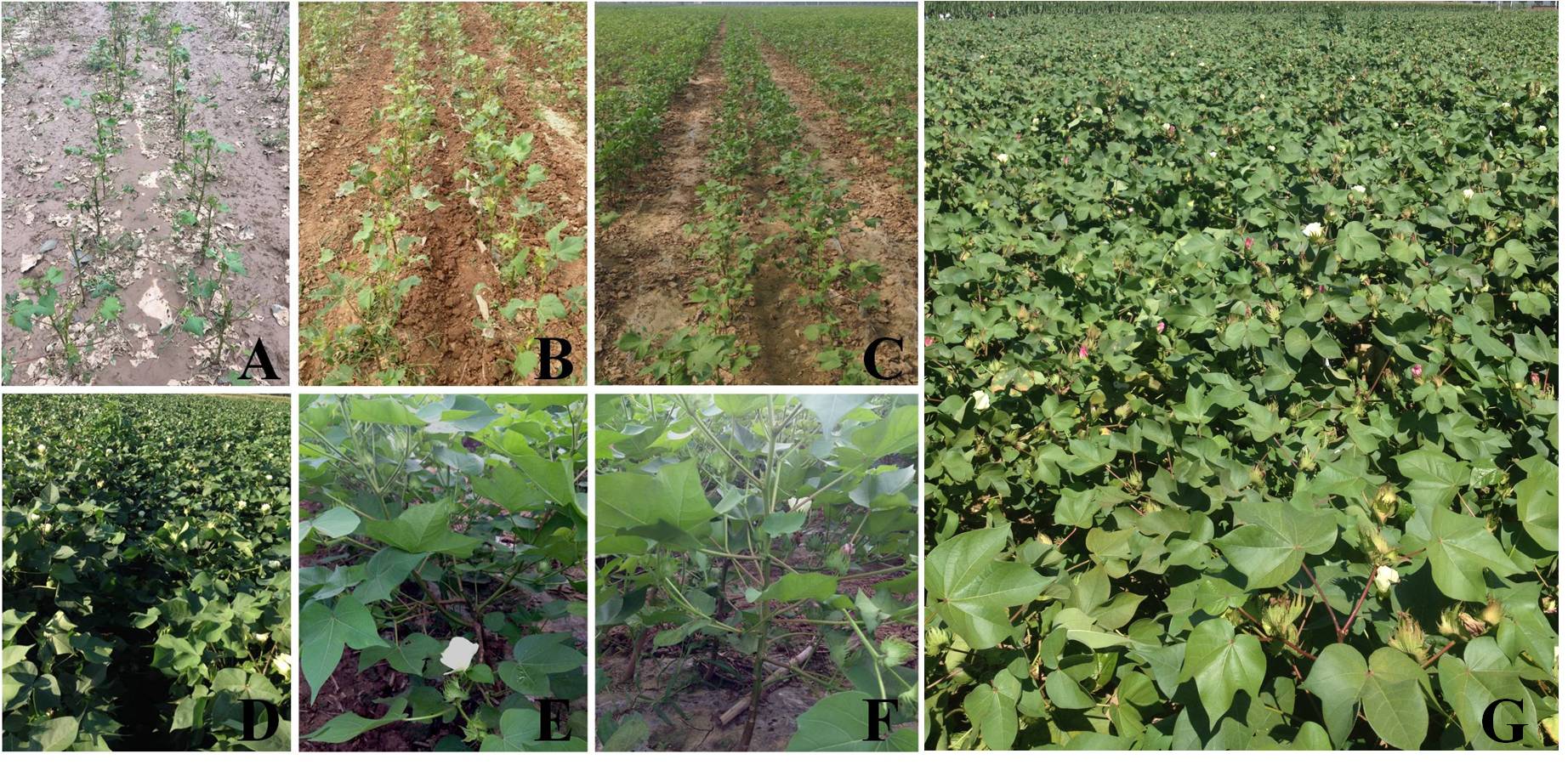


**Supplementary Table S1. Correlation analysis for yield and yield-components traits in BC, MPH and RIL datasets in both BC/M and BC/P trials.** Data presents correlation value in BC/M trial / correlation value in BC/P trial, respectively. Env., the environment in BC/M trial, and another corresponding environment in BC/P trial. Critical values for correlation coefficients at probabilities of 0.05 and 0.01 are 0.14 and 0.19, respectively.

| **Trait** | **Env.** | **BC and MPH** | **RIL and BC** | **RIL and MPH** |
| --- | --- | --- | --- | --- |
| SY | 2015E1/2016E1 | 0.67/0.69 | 0.43/0.56 | -0.12/-0.10 |
|  | 2015E2/2015E2 | 0.11/0.28 | 0.41/0.33 | -0.07/-0.07 |
|  | 2016E2/2016E2 | 0.56/0.62 | 0.50/0.57 | -0.25/-0.18 |
|  | 2015E3/ - | 0.64/ - | 0.45/ - | -0.21/ - |
| LY | 2015E1/2016E1 | 0.67/0.71 | 0.42/0.59 | -0.08/-0.04 |
|  | 2015E2/2015E2 | 0.11/0.26 | 0.19/0.32 | -0.09/-0.07 |
|  | 2016E2/2016E2 | 0.63/0.62 | 0.52/0.57 | -0.22/-0.18 |
|  | 2015E3/ - | 0.64/ - | 0.48/ - | -0.18/ - |
| BNP | 2015E1/2016E1 | 0.67/0.58 | 0.38/0.38 | -0.19/-0.35 |
|  | 2015E2/2015E2 | 0.59/0.68 | 0.56/0.44 | -0.12/-0.18 |
|  | 2016E2/2016E2 | 0.68/0.67 | 0.30/0.31 | -0.28/-0.32 |
|  | 2015E3/ - | 0.52/ - | 0.35/ - | -0.39/ - |
| BW | 2015E1/2016E1 | 0.65/0.73 | 0.39/0.41 | -0.31/-0.20 |
|  | 2015E2/2015E2 | 0.57/0.64 | 0.48/0.34 | -0.25/-0.34 |
|  | 2016E2/2016E2 | 0.61/0.61 | 0.50/0.55 | -0.38/-0.19 |
|  | 2015E3/ - | 0.62/ - | 0.39/ - | -0.29/ - |
| LP | 2015E1/2016E1 | 0.57/0.49 | 0.62/0.68 | -0.16/-0.14 |
|  | 2015E2/2015E2 | 0.46/0.40 | 0.80/0.63 | -0.07/-0.30 |
|  | 2016E2/2016E2 | 0.56/0.45 | 0.66/0.67 | -0.23/-0.25 |
|  | 2015E3/ - | 0.44/ - | 0.48/ - | -0.16/ - |

**Table S2. Single locus QTLs for yield and yield-component traits in BC, MPH and RIL datasets in both BC trials by composite interval mapping (CIM) method.** SY, seed-cotton yield per plant, LY, lint yield per plant, BNP, boll number per plant, BW, boll weight, LP, lint percentage, trait abbreviate. Chr, chromosome. Var %, the phenotypic variation explained by a single QTL. Effect value, the phenotypic effect of a single QTL or a heterotic QTL, it referred to additive effect in RIL population, the total effect in BC population, the dominance effect in MPH dataset. Bold figures indicated common QTL verified in more than one environment or population. The underlined data indicated that QTLs were detected in BC/M trial, the remaining data without underline indicated that QTLs were detected in BC/P trial. QTL within * for seed yield trait was common to main-effect QTL (M-QTLs) by inclusive composite interval mapping (ICIM) method in Table S6-A,-C,-E.

| **QTL** | **Env.** | **Flanking makers** | | **RIL** | | |  | **BC** | | |  | **MPH** | | | ***d/a*** | **Type** |
| --- | --- | --- | --- | --- | --- | --- | --- | --- | --- | --- | --- | --- | --- | --- | --- | --- |
|  |  |  |  | **LOD** | **Effect value** | **Var%** |  | **LOD** | **Effect value** | **Var%** |  | **LOD** | **Effect value** | **Var%** |  |  |
| ***qSY-Chr2-1**** | 2015E2 | SWU11887 | SWU11976 | 3.23 | 3.92 | 7.11 |  |  |  |  |  |  |  |  |  |  |
|  | 2015E2 | SWU11887 | SWU11976 | 2.89 | 4.53 | 7.46 |  |  |  |  |  |  |  |  |  |  |
|  | 2016E1 | SWU11889 | SWU11887 |  | (±2.46) | |  | 8.88 | 6.46 | 27.26 |  | 4.46 | 4.00 | 12.41 | 1.63 | OD |
|  | 2016E2 | SWU11889 | SWU11887 |  |  |  |  |  |  |  |  | 3.10 | 2.61 | 7.01 |  | OD |
| ***qSY-Chr4-1*** | 2016E2 | SWU21485 | ICR01729 | 3.25 | 3.68 | 6.31 |  | 2.84 | 3.07 | 6.03 |  |  | (±0.61) |  | 0.17 | PD |
| *qSY-Chr5-1** | 2016E2 | SWU20917 | NAU6240 |  |  |  |  | 3.63 | 3.28 | 9.41 |  |  |  |  |  | A |
| *qSY-Chr5-2* | 2015E2 | SWU13378 | SWU17846 |  |  |  |  |  |  |  |  | 4.90 | -3.73 | 10.10 |  | OD |
| ***qSY-Chr6-1*** | 2015E2 | ICR03206 | NAU896 |  |  |  |  |  |  |  |  | 3.42 | -3.09 | 6.90 |  | OD |
|  | 2016E2 | ICR03206 | NAU896 | 2.81 | 3.53 | 5.96 |  |  |  |  |  |  |  |  |  |  |
| *qSY-Chr11-1* | 2016E2 | NAU3390 | NAU2460 |  |  |  |  |  |  |  |  | 3.56 | -2.62 | 7.43 |  | OD |
| ***qSY-Chr20-1*** | 2015E2 | SWU20501a | CER0167 | 3.78 | -4.49 | 7.36 |  |  |  |  |  |  |  |  |  |  |
|  | 2016E1 | SWU20246 | SWU20501a | 2.56 | -4.01 | 6.73 |  |  |  |  |  |  |  |  |  |  |
| ***qSY-Chr21-1*** | 2015E1 | CGR5808 | HAU0423 |  |  |  |  | 3.86 | 3.03 | 9.33 |  |  |  |  |  | A |
|  | 2015E3 | CGR5808 | HAU0423 | 4.58 | -4.19 | 9.20 |  |  |  |  |  |  |  |  |  |  |
| ***qSY-Chr21-2*** | 2015E1 | CGR5806 | DPL0777 |  |  |  |  | 2.63 | -2.66 | 6.96 |  |  |  |  |  | A |
|  | 2016E1 | CGR5806 | DPL0777 | 3.06 | -4.84 | 9.78 |  |  |  |  |  |  |  |  |  |  |
| *qSY-Chr25-1* | 2015E2 | NAU3112 | SWU19144 |  |  |  |  |  |  |  |  | 3.66 | -3.62 | 7.41 |  | OD |
| ***qLY-Chr1-1*** | 2015E2 | NAU3177 | ICR03724 |  |  |  |  | 2.58 | -1.27 | 5.44 |  |  |  |  |  | A |
|  | 2016E2 | NAU3177 | ICR03724 | 3.37 | -1.67 | 7.30 |  |  |  |  |  |  |  |  |  |  |
| ***qLY-Chr2-1*** | 2015E1 | SWU12025 | SWU11889 | 4.72 | 4.11 | 19.42 |  |  |  |  |  |  |  |  |  |  |
|  | 2015E2 | SWU11887 | SWU11976 | 3.83 | 1.66 | 7.75 |  |  |  |  |  |  |  |  |  |  |
|  | 2015E2 | SWU11887 | SWU11976 | 3.66 | 1.90 | 8.85 |  |  |  |  |  |  |  |  |  |  |
|  | 2016E1 | SWU11889 | SWU11887 |  | (±0.68) | |  | 6.68 | 2.23 | 19.61 |  |  |  |  | 2.31 | OD |
|  | 2016E1 | SWU11976 | SWU11950 |  |  |  |  |  |  |  |  | 5.15 | 1.56 | 12.85 |  |  |
|  | 2016E2 | SWU11887 | SWU11976 |  | (±0.24) | |  | 3.20 | 1.42 | 7.81 |  | 2.86 | 1.18 | 8.03 | 4.89 | OD |
| ***qLY-Chr2-2*** | 2016E1 | PGML0700 | SWU12016 |  | (±0.22) | |  | 2.54 | 1.67 | 9.77 |  | 4.51 | 1.89 | 18.79 | 8.64 | OD |
| ***qLY-Chr2-3*** | 2015E2 | ICR11064 | NAU935 |  |  |  |  | 2.67 | 1.12 | 5.68 |  |  |  |  |  | A |
|  | 2016E2 | ICR11064 | NAU935 |  |  |  |  |  |  |  |  | 2.65 | 0.99 | 5.64 |  | OD |
| *qLY-Chr5-1* | 2015E2 | SWU13378 | SWU17846 |  |  |  |  |  |  |  |  | 3.72 | -1.22 | 7.48 |  | OD |
| *qLY-Chr11-1* | 2016E2 | NAU3390 | NAU2460 |  |  |  |  |  |  |  |  | 3.88 | -1.24 | 9.10 |  | OD |
| *qLY-Chr12-1* | 2015E2 | C2_0115 | ICR03107 | 3.37 | 1.93 | 9.12 |  |  |  |  |  |  |  |  |  |  |
| ***qLY-Chr13-1*** | 2015E1 | PGML0014 | CGR6732 |  | (±0.05) | |  | 2.62 | -0.98 | 5.95 |  | 3.03 | -1.03 | 6.46 | 18.90 | OD |
| *qLY-Chr20-1* | 2015E2 | SWU20501a | CER0167 | 3.48 | -1.65 | 6.70 |  |  |  |  |  |  |  |  |  |  |
| *qLY-Chr21-1* | 2016E2 | SWU16489 | SWU16487 |  |  |  |  |  |  |  |  | 3.48 | 1.13 | 7.57 |  | OD |
| ***qLY-Chr21-2*** | 2015E1 | HAU0423 | CGR5806 |  |  |  |  |  |  |  |  | 3.72 | 1.03 | 8.02 |  | OD |
|  | 2015E3 | HAU0423 | CGR5806 | 4.92 | -1.78 | 9.82 |  |  |  |  |  |  |  |  |  |  |
|  | 2016E1 | CGR5806 | DPL0777 | 3.65 | -2.40 | 16.15 |  |  |  |  |  |  |  |  |  |  |
|  | 2016E2 | CGR5806 | DPL0777 | 3.29 | -2.53 | 16.66 |  |  |  |  |  |  |  |  |  |  |
| *qLY-Chr21-3* | 2015E2 | BNL3442a | ICR08249 | 3.88 | -1.78 | 7.52 |  |  |  |  |  |  |  |  |  |  |
| *qLY-Chr25-1* | 2015E2 | NAU3112 | SWU19144 |  |  |  |  |  |  |  |  | 4.82 | -1.71 | 9.95 |  | OD |
| *qBNP-Chr1-1* | 2016E2 | SWU10912 | DPL0090 | 3.94 | -0.91 | 11.17 |  |  |  |  |  |  |  |  |  |  |
| *qBNP-Chr1-2* | 2015E2 | NAU3177 | ICR03724 |  |  |  |  | 3.77 | -0.69 | 7.97 |  |  |  |  |  | A |
| ***qBNP-Chr1-3*** | 2015E3 | CGR6857 | ICR11205 | 3.77 | 0.76 | 7.52 |  |  |  |  |  |  |  |  |  |  |
|  | 2016E1 | CGR6857 | ICR11205 | 2.72 | 0.93 | 5.92 |  |  |  |  |  |  |  |  |  |  |
| ***qBNP-Chr1-4*** | 2016E1 | SWU11191 | BNL2827b |  | (±0.49) |  |  | 3.07 | -0.78 | 6.67 |  | 7.20 | -1.27 | 14.68 | 2.60 | OD |
| *qBNP-Chr2-1* | 2015E1 | SWU12025 | SWU11889 | 4.11 | 1.69 | 16.59 |  |  |  |  |  |  |  |  |  |  |
| ***qBNP-Chr2-2*** | 2015E1 | SWU11887 | SWU11976 | 5.02 | 1.33 | 10.21 |  |  |  |  |  |  |  |  |  |  |
|  | 2015E2 | SWU11887 | SWU11976 | 3.22 | 0.75 | 7.43 |  |  |  |  |  |  |  |  |  |  |
| *qBNP-Chr2-3* | 2015E1 | TMB1268 | JESPR304 |  |  |  |  |  |  |  |  | 3.87 | -0.65 | 8.33 |  | OD |
| *qBNP-Chr5-1* | 2015E1 | SWU20917 | NAU6240 | 7.55 | -1.01 | 19.85 |  |  |  |  |  |  |  |  |  |  |
| *qBNP-Chr6-1* | 2016E1 | NAU1218 | SWU19654 |  |  |  |  | 3.64 | -1.07 | 7.40 |  |  |  |  |  | A |
| *qBNP-Chr6-2* | 2016E1 | SWU19541 | CGR5801 |  |  |  |  | 3.47 | -1.02 | 7.28 |  |  |  |  |  | A |
| *qBNP-Chr6-3* | 2016E1 | CGR5124 | HAU1460 |  |  |  |  | 7.01 | 1.40 | 14.34 |  |  |  |  |  | A |
| *qBNP-Chr13-1* | 2015E1 | NAU2893 | Gh157 | 5.49 | -0.74 | 10.08 |  |  |  |  |  |  |  |  |  |  |
| *qBNP-Chr14-1* | 2015E1 | SWU13824 | CGR6683 | 2.83 | 0.51 | 5.00 |  |  |  |  |  |  |  |  |  |  |
| ***qBNP-Chr14-2*** | 2015E2 | SWU14224 | DPL0565 | 2.51 | 0.64 | 5.35 |  |  |  |  |  |  |  |  |  |  |
|  | 2015E3 | SWU14224 | DPL0565 | 2.84 | 0.63 | 6.76 |  |  |  |  |  |  |  |  |  |  |
| ***qBNP-Chr15-1*** | 2016E1 | CGR6889 | DPL0182 |  |  |  |  |  |  |  |  | 4.05 | 0.91 | 7.82 |  | OD |
|  | 2016E2 | DPL0182 | SWU11691 | 2.70 | -0.63 | 5.43 |  |  |  |  |  |  |  |  |  |  |
| *qBNP-Chr16-1* | 2015E3 | C2_0011B | DC20124 |  |  |  |  | 3.43 | -0.62 | 7.15 |  |  |  |  |  | A |
| *qBNP-Chr19-1* | 2015E3 | NAU3437 | NAU2894 | 3.66 | 0.74 | 7.44 |  |  |  |  |  |  |  |  |  |  |
| *qBNP-Chr20-1* | 2015E2 | SWU20501a | CER0167 | 4.35 | -0.87 | 8.94 |  |  |  |  |  |  |  |  |  |  |
| ***qBNP-Chr21-1*** | 2015E3 | SWU16138 | BNL1053 | 2.54 | 0.71 | 6.61 |  | 2.85 | 0.60 | 6.47 |  |  | (±0.11) | | 0.15 | PD |
| ***qBNP-Chr21-2*** | 2015E1 | CGR5808 | HAU0423 |  |  |  |  | 2.95 | 0.62 | 8.88 |  |  |  |  |  | A |
|  | 2016E2 | HAU0423 | CGR5806 |  |  |  |  | 4.27 | -1.02 | 12.22 |  |  |  |  |  | A |
| *qBNP-Chr21-3* | 2015E2 | BNL3442a | ICR08249 | 3.41 | -0.78 | 6.90 |  |  |  |  |  |  |  |  |  |  |
| *qBNP-Chr24-1* | 2015E3 | Gh268 | SWU13268 | 4.28 | 0.73 | 9.11 |  |  |  |  |  |  |  |  |  |  |
| *qBNP-Chr25-1* | 2016E2 | SWU19815 | BNL3594 |  |  |  |  | 4.49 | 0.81 | 10.56 |  |  |  |  |  | A |
| *qBW-Chr1-1* | 2015E2 | HAU1417 | NAU2437 |  |  |  |  |  |  |  |  | 5.74 | -0.12 | 12.62 |  | OD |
| ***qBW-Chr2-1*** | 2015E2 | SWU11889 | SWU11887 | 2.54 | 0.13 | 7.12 |  |  |  |  |  |  |  |  |  |  |
|  | 2016E2 | SWU11889 | SWU11887 |  |  |  |  | 4.68 | 0.11 | 11.22 |  |  |  |  |  | A |
| ***qBW-Chr5-1*** | 2015E1 | SWU20913 | Gh260 |  |  |  |  | 4.83 | 0.13 | 13.51 |  |  |  |  |  | A |
|  | 2015E3 | PGML0120 | SWU20914 |  |  |  |  | 3.73 | 0.08 | 7.60 |  |  |  |  |  | A |
|  | 2016E1 | SWU20913 | Gh260 |  | (±0.03) | |  | 5.24 | 0.13 | 10.95 |  | 2.67 | 0.10 | 6.09 | 3.58 | OD |
| ***qBW-Chr5-2*** | 2015E2 | SWU20917 | NAU6240 | 2.63 | 0.13 | 7.18 |  |  |  |  |  |  |  |  |  |  |
|  | 2015E3 | SWU20917 | NAU6240 | 3.68 | 0.14 | 11.38 |  | 4.00 | 0.10 | 12.34 |  |  | (±0.04) | | 0.28 | PD |
|  | 2016E2 | SWU20917 | NAU6240 | 4.61 | 0.15 | 13.31 |  | 3.93 | 0.11 | 11.11 |  |  | (±0.02) | | 0.18 | PD |
|  | 2016E2 | SWU20917 | NAU6240 | 3.66 | 0.13 | 9.06 |  |  |  |  |  |  |  |  |  |  |
| ***qBW-Chr6-1*** | 2015E3 | HAU1460 | HAU1371 |  |  |  |  | 2.82 | -0.07 | 5.69 |  |  |  |  |  | A |
|  | 2016E2 | HAU1460 | HAU1371 |  |  |  |  | 3.21 | -0.10 | 6.70 |  |  |  |  |  | A |
| *qBW-Chr11-1* | 2016E1 | ICR01810 | CGR6525 | 3.42 | -0.13 | 7.33 |  |  |  |  |  |  |  |  |  |  |
| ***qBW-Chr20-1*** | 2015E2 | CER0167 | SWU20064 | 2.76 | -0.11 | 5.52 |  |  |  |  |  |  |  |  |  |  |
|  | 2015E3 | SWU20501a | CER0167 | 2.58 | -0.11 | 7.14 |  |  |  |  |  |  |  |  |  |  |
|  | 2016E1 | SWU20246 | SWU20501a |  |  |  |  | 3.77 | -0.11 | 7.46 |  |  |  |  |  | A |
|  | 2016E2 | SWU20246 | SWU20501a | 4.58 | -0.15 | 11.55 |  |  |  |  |  |  |  |  |  |  |
| ***qBW-Chr23-1*** | 2015E2 | SWU14807 | PGML4185 |  |  |  |  | 2.88 | 0.08 | 5.60 |  |  |  |  |  | A |
|  | 2015E2 | PGML4186 | NAU3100 |  | (±0.01) | |  | 2.52 | 0.08 | 5.61 |  | 2.54 | 0.08 | 5.42 | 15.98 | OD |
| *qBW-Chr26-1* | 2015E2 | SWU16676 | SWU16755 |  |  |  |  | 4.94 | 0.14 | 17.04 |  |  |  |  |  | A |
| ***qLP-Chr4-1*** | 2015E1 | SWU18876 | SWU12672 |  |  |  |  |  |  |  |  | 2.70 | 0.32 | 5.80 |  | OD |
|  | 2015E3 | SWU18881 | NAU2701 | 3.44 | -0.53 | 6.92 |  |  |  |  |  |  |  |  |  |  |
| ***qLP-Chr5-1*** | 2015E2 | SWU20913 | Gh260 | 5.19 | -0.66 | 9.73 |  | 6.57 | -0.50 | 13.75 |  |  | (±0.16) | | 0.24 | PD |
|  | 2015E3 | SWU20913 | Gh260 |  |  |  |  | 7.59 | -0.59 | 15.76 |  |  |  |  |  | A |
|  | 2016E1 | PGML0120 | SWU20914 |  |  |  |  | 7.33 | -0.67 | 14.23 |  |  |  |  |  | A |
| ***qLP-Chr5-2*** | 2015E1 | SWU20917 | NAU6240 | 4.03 | 0.17 | 11.27 |  |  |  |  |  |  |  |  |  |  |
|  | 2015E1 | NAU6240 | PGML1671 |  |  |  |  | 5.00 | -0.60 | 14.15 |  |  | (±0.77) | | 4.46 | OD |
|  | 2015E2 | SWU20917 | NAU6240 | 5.58 | -0.80 | 14.22 |  |  |  |  |  |  |  |  |  |  |
|  | 2015E2 | SWU20917 | NAU6240 | 7.59 | -0.99 | 18.75 |  | 5.88 | -0.60 | 15.42 |  |  | (±0.38) | | 0.39 | PD |
|  | 2015E3 | SWU20917 | NAU6240 |  |  |  |  | 8.04 | -0.71 | 22.38 |  |  | (±0.32) | | 0.31 | PD |
|  | 2015E3 | SWU20917 | NAU6240 | 9.06 | -1.03 | 26.92 |  |  |  |  |  |  |  |  |  |  |
|  | 2016E1 | PGML1671 | PGML1917 |  |  |  |  |  |  |  |  | 2.62 | -0.28 | 5.18 |  |  |
|  | 2016E1 | SWU20917 | NAU6240 | 7.36 | -1.13 | 18.80 |  | 7.75 | -0.81 | 20.82 |  |  |  |  | 0.53 | PD |
|  | 2016E2 | SWU20917 | NAU6240 | 7.45 | -1.06 | 20.01 |  | 5.16 | -0.52 | 10.78 |  |  | (±0.54) | | 0.51 | PD |
|  | 2016E2 | SWU20917 | NAU6240 | 9.73 | -1.04 | 20.51 |  | 7.78 | -0.69 | 19.50 |  |  | (±0.35) | | 0.34 | PD |
| ***qLP-Chr5-3*** | 2015E1 | HAU1603 | PGML4457 | 3.35 | 0.13 | 6.32 |  |  |  |  |  |  |  |  |  |  |
|  | 2015E2 | TMB1296 | HAU1603 | 2.68 | -0.51 | 4.96 |  |  |  |  |  |  |  |  |  |  |
|  | 2016E1 | TMB1296 | HAU1603 | 2.54 | -0.55 | 4.35 |  |  |  |  |  |  |  |  |  |  |
| ***qLP-Chr7-1*** | 2015E1 | SWU10064 | NAU3181 |  |  |  |  | 3.72 | -0.46 | 8.11 |  |  |  |  |  | A |
|  | 2016E2 | SWU10064 | NAU3181 |  |  |  |  |  |  |  |  | 2.70 | 0.32 | 7.71 |  | OD |
| *qLP-Chr13-1* | 2015E1 | PGML0014 | CGR6732 |  |  |  |  | 3.42 | -0.41 | 6.36 |  |  |  |  |  | A |
| ***qLP-Chr13-2*** | 2015E3 | NAU2893 | Gh157 | 3.73 | -0.57 | 7.89 |  |  |  |  |  |  |  |  |  |  |
|  | 2016E1 | NAU2893 | Gh157 |  |  |  |  | 2.67 | -0.41 | 4.98 |  |  |  |  |  | A |
|  | 2016E2 | NAU2893 | Gh157 | 2.68 | -0.57 | 5.38 |  |  |  |  |  |  |  |  |  |  |
| ***qLP-Chr13-3*** | 2015E2 | BNL1495 | CGR5390 | 4.79 | -0.72 | 11.27 |  |  |  |  |  | 4.75 | 0.46 | 13.26 | 0.64 | PD |
|  | 2015E2 | BNL1495 | CGR5390 | 3.54 | -0.66 | 8.21 |  |  |  |  |  |  |  |  |  |  |
|  | 2015E3 | BNL1495 | CGR5390 | 2.99 | -0.54 | 7.05 |  |  |  |  |  |  |  |  |  |  |
|  | 2016E1 | BNL1495 | CGR5390 | 6.21 | -0.95 | 13.25 |  |  |  |  |  |  |  |  |  |  |
|  | 2016E2 | BNL1495 | CGR5390 | 3.00 | -0.61 | 6.44 |  |  |  |  |  |  |  |  |  |  |
|  | 2016E2 | DPL0894 | SWU10800 |  |  |  |  | 5.17 | -0.65 | 17.59 |  |  | (±0.04) | | 0.07 | PD |
| ***qLP-Chr14-1*** | 2015E1 | CGR6683 | NAU3308 |  |  |  |  | 2.71 | 0.36 | 4.99 |  |  |  |  |  | A |
|  | 2016E1 | NAU874 | SWU13824 | 3.00 | 0.64 | 5.83 |  |  |  |  |  |  |  |  |  |  |
| *qLP-Chr14-2* | 2016E1 | SWU14224 | DPL0565 | 3.52 | 0.74 | 7.89 |  |  |  |  |  |  |  |  |  |  |
| *qLP-Chr18-1* | 2016E1 | SWU22187 | DC40150 |  |  |  |  |  |  |  |  | 4.27 | 0.38 | 9.41 |  | OD |
| ***qLP-Chr19-1*** | 2015E2 | NAU3437 | NAU2894 |  |  |  |  | 2.88 | 0.37 | 5.68 |  |  |  |  |  | A |
|  | 2016E1 | NAU3437 | NAU2894 | 2.69 | 1.03 | 15.67 |  |  |  |  |  |  |  |  |  |  |
|  | 2016E2 | NAU3437 | NAU2894 | 3.00 | 0.65 | 8.11 |  |  |  |  |  |  |  |  |  |  |
| *qLP-Chr20-1* | 2015E1 | CER0167 | SWU20064 | 4.23 | -0.15 | 8.11 |  |  |  |  |  |  |  |  |  |  |
| *qLP-Chr21-1* | 2015E2 | BNL3171 | CGR5808 |  |  |  |  |  |  |  |  | 2.80 | -0.33 | 6.59 |  | OD |
| ***qLP-Chr21-2*** | 2016E2 | CGR5806 | DPL0777 | 2.53 | -0.74 | 9.87 |  |  |  |  |  |  |  |  |  |  |
|  | 2016E2 | CGR5806 | DPL0777 |  |  |  |  | 3.08 | -0.45 | 8.70 |  |  |  |  |  | A |
| *qLP-Chr23-1* | 2016E2 | Gh327 | SWU14770 |  |  |  |  |  |  |  |  | 3.33 | 0.33 | 7.13 |  | OD |

**Supplementary Table S3. Correlation analyses between overall marker heterozygosity and trait performance for yield and yield-components traits in BC and MPH datasets.** Data presents correlation value in BC/M trial / correlation value in BC/P trial, respectively. Env., the environment in BC/M trial, and another corresponding environment in BC/P trial.

| **Env.** | **Population** | **SY** | **LY** | **BNP** | **BW** | **LP** |
| --- | --- | --- | --- | --- | --- | --- |
| 2015E1/2016E1 | BC | -0.05/0.02 | -0.04/0.02 | -0.10/0.01 | -0.08/-0.06 | 0.01/0.05 |
|  | MPH | 0.04/-0.01 | 0.01/-0.03 | -0.08/0.01 | -0.02/-0.01 | -0.10/0.02 |
| 2015E2/2015E2 | BC | -0.01/0.12 | -0.01/0.14 | 0.03/0.09 | -0.14/0.02 | 0.02/0.09 |
|  | MPH | 0.01/0.01 | 0.01/-0.01 | -0.08/0.07 | -0.16/0.004 | -0.04/0.11 |
| 2016E2/2016E2 | BC | 0.00/-0.04 | 0.00/-0.02 | -0.03/-0.02 | -0.05/0.04 | 0.01/0.04 |
|  | MPH | 0.02/-0.02 | 0.01/0.01 | -0.09/-0.09 | -0.03/0.04 | -0.05/0.11 |
| 2015E3/ - | BC | 0.04/ - | 0.03/ - | 0.09/ - | 0.03/ - | -0.07/ - |
|  | MPH | 0.02/ - | 0.02/ - | 0.08/ - | -0.03/ - | 0.14/ - |

**Supplementary Table S4. Clusters in present study for five yield and yield-component traits.** See footnotes in Table S2.

| **Cluster** | **QTL** | **Env.** | **Flanking makers** | | **BC** | | | |  | **MPH** | | |  | **RIL** | | |
| --- | --- | --- | --- | --- | --- | --- | --- | --- | --- | --- | --- | --- | --- | --- | --- | --- |
|  |  |  |  |  | **LOD** | **Effect value** | | **Var%** |  | **LOD** | **Effect value** | **Var %** |  | **LOD** | **Effect value** | **Var%** |
| *Cluster-Chr1-1* | ***qLY-Chr1-1*** | 2015E2 | NAU3177 | ICR03724 | 2.58 | -1.27 | 5.44 | |  |  |  |  |  |  |  |  |
|  |  | 2016E2 | NAU3177 | ICR03724 |  |  |  | |  |  |  |  |  | 3.37 | -1.67 | 7.30 |
|  | *qBNP-Chr1-2* | 2015E2 | NAU3177 | ICR03724 | 3.77 | -0.69 | 7.97 | |  |  |  |  |  |  |  |  |
| *Cluster-Chr2-1* | ***qSY-Chr2-1*** | 2015E2 | SWU11887 | SWU11976 |  |  |  | |  |  |  |  |  | 3.23 | 3.92 | 7.11 |
|  |  | 2015E2 | SWU11887 | SWU11976 |  |  |  | |  |  |  |  |  | 2.89 | 4.53 | 7.46 |
|  |  | 2016E1 | SWU11889 | SWU11887 | 8.88 | 6.46 | 27.26 | |  | 4.46 | 4.00 | 12.41 |  |  | (±2.46) |  |
|  |  | 2016E2 | SWU11889 | SWU11887 |  |  |  | |  | 3.10 | 2.61 | 7.01 |  |  |  |  |
|  | ***qLY-Chr2-1*** | 2015E1 | SWU12025 | SWU11889 |  |  |  | |  |  |  |  |  | 4.72 | 4.11 | 19.42 |
|  |  | 2015E2 | SWU11887 | SWU11976 |  |  |  | |  |  |  |  |  | 3.83 | 1.66 | 7.75 |
|  |  | 2015E2 | SWU11887 | SWU11976 |  |  |  | |  |  |  |  |  | 3.66 | 1.90 | 8.85 |
|  |  | 2016E1 | SWU11889 | SWU11887 | 6.68 | 2.23 | 19.61 | |  | 5.15 | 1.56 | 12.85 |  |  | (±0.68) |  |
|  |  | 2016E2 | SWU11887 | SWU11976 | 3.20 | 1.42 | 7.81 | |  | 2.86 | 1.18 | 8.03 |  |  | (±0.24) |  |
|  | *qBNP-Chr2-1* | 2015E1 | SWU12025 | SWU11889 |  |  |  | |  |  |  |  |  | 4.11 | 1.69 | 16.59 |
|  | ***qBNP-Chr2-2*** | 2015E1 | SWU11887 | SWU11976 |  |  |  | |  |  |  |  |  | 5.02 | 1.33 | 10.21 |
|  |  | 2015E2 | SWU11887 | SWU11976 |  |  |  | |  |  |  |  |  | 3.22 | 0.75 | 7.43 |
|  | ***qBW-Chr2-1*** | 2015E2 | SWU11889 | SWU11887 |  |  |  | |  |  |  |  |  | 2.54 | 0.13 | 7.12 |
|  |  | 2016E2 | SWU11889 | SWU11887 | 4.68 | 0.11 | 11.22 | |  |  |  |  |  |  |  |  |
| *Cluster-Chr2-2* | ***qLY-Chr2-2*** | 2016E1 | PGML0700 | SWU12016 | 2.54 | 1.67 | 9.77 | |  | 4.51 | 1.89 | 18.79 |  |  | (±0.22) |  |
|  | *qBNP-Chr2-3* | 2015E1 | TMB1268 | JESPR304 |  |  |  | |  | 3.87 | -0.65 | 8.33 |  |  |  |  |
| *Cluster-Chr5-1* | ***qBW-Chr5-1*** | 2015E1 | SWU20913 | Gh260 | 4.83 | 0.13 | 13.51 | |  |  |  |  |  |  |  |  |
|  |  | 2015E3 | PGML0120 | SWU20914 | 3.73 | 0.08 | 7.60 | |  |  |  |  |  |  |  |  |
|  |  | 2016E1 | SWU20913 | Gh260 | 5.24 | 0.13 | 10.95 | |  | 2.67 | 0.10 | 6.09 |  |  | (±0.03) |  |
|  | ***qLP-Chr5-1*** | 2015E2 | SWU20913 | Gh260 | 6.57 | -0.50 | 13.75 | |  |  | (±0.16) |  |  | 5.19 | -0.66 | 9.73 |
|  |  | 2015E3 | SWU20913 | Gh260 | 7.59 | -0.59 | 15.76 | |  |  |  |  |  |  |  |  |
|  |  | 2016E1 | PGML0120 | SWU20914 | 7.33 | -0.67 | 14.23 | |  |  |  |  |  |  |  |  |
| *Cluster-Chr5-2* | *qSY-Chr5-1* | 2016E2 | SWU20917 | NAU6240 | 3.63 | 3.28 | 9.41 | |  |  |  |  |  |  |  |  |
|  | *qBNP-Chr5-1* | 2015E1 | SWU20917 | NAU6240 |  |  |  | |  |  |  |  |  | 7.55 | -1.01 | 19.85 |
|  | ***qBW-Chr5-2*** | 2015E2 | SWU20917 | NAU6240 |  |  |  | |  |  |  |  |  | 2.63 | 0.13 | 7.18 |
|  |  | 2015E3 | SWU20917 | NAU6240 | 4.00 | 0.10 | 12.34 | |  |  | (±0.04) |  |  | 3.68 | 0.14 | 11.38 |
|  |  | 2016E2 | SWU20917 | NAU6240 | 3.93 | 0.11 | 11.11 | |  |  | (±0.02) |  |  | 4.61 | 0.15 | 13.31 |
|  |  | 2016E2 | SWU20917 | NAU6240 |  |  |  | |  |  |  |  |  | 3.66 | 0.13 | 9.06 |
|  | ***qLP-Chr5-2*** | 2015E1 | SWU20917 | NAU6240 | 5.00 | -0.60 | 14.15 | |  |  | (±0.77) |  |  | 4.03 | 0.17 | 11.27 |
|  |  | 2015E2 | SWU20917 | NAU6240 |  |  |  | |  |  |  |  |  | 5.58 | -0.80 | 14.22 |
|  |  | 2015E2 | SWU20917 | NAU6240 | 5.88 | -0.60 | 15.42 | |  |  |  |  |  | 7.59 | -0.99 | 18.75 |
|  |  | 2015E3 | SWU20917 | NAU6240 | 8.04 | -0.71 | 22.38 | |  |  |  |  |  | 9.06 | -1.03 | 26.92 |
|  |  | 2016E1 | PGML1671 | PGML1917 | 7.75 | -0.81 | 20.82 | |  | 2.62 | -0.28 | 5.18 |  | 7.36 | -1.13 | 18.80 |
|  |  | 2016E2 | SWU20917 | NAU6240 | 5.16 | -0.52 | 10.78 | |  |  | (±0.54) |  |  | 7.45 | -1.06 | 20.01 |
|  |  | 2016E2 | SWU20917 | NAU6240 | 7.78 | -0.69 | 19.50 | |  |  | (±0.35) |  |  | 9.73 | -1.04 | 20.51 |
| *Cluster-Chr5-3* | *qSY-Chr5-2* | 2015E2 | SWU13378 | SWU17846 |  |  |  | |  | 4.90 | -3.73 | 10.10 |  |  |  |  |
|  | *qLY-Chr5-1* | 2015E2 | SWU13378 | SWU17846 |  |  |  | |  | 3.72 | -1.22 | 7.48 |  |  |  |  |
| *Cluster-Chr6-1* | *qBNP-Chr6-3* | 2016E1 | CGR5124 | HAU1460 | 7.01 | 1.40 | 14.34 | |  |  |  |  |  |  |  |  |
|  | ***qBW-Chr6-1*** | 2015E3 | HAU1460 | HAU1371 | 2.82 | -0.07 | 5.69 | |  |  |  |  |  |  |  |  |
|  |  | 2016E2 | HAU1460 | HAU1371 | 3.21 | -0.10 | 6.70 | |  |  |  |  |  |  |  |  |
| *Cluster-Chr11-1* | *qSY-Chr11-1* | 2016E2 | NAU3390 | NAU2460 |  |  |  | |  | 3.56 | -2.62 | 7.43 |  |  |  |  |
|  | *qLY-Chr11-1* | 2016E2 | NAU3390 | NAU2460 |  |  |  | |  | 3.88 | -1.24 | 9.10 |  |  |  |  |
| *Cluster-Chr13-1* | ***qLY-Chr13-1*** | 2015E1 | PGML0014 | CGR6732 | 2.62 | -0.98 | 5.95 | |  | 3.03 | -1.03 | 6.46 |  |  |  |  |
|  | *qBNP-Chr13-1* | 2015E1 | NAU2893 | Gh157 |  |  |  | |  |  |  |  |  | 5.49 | -0.74 | 10.08 |
|  | *qLP-Chr13-1* | 2015E1 | PGML0014 | CGR6732 | 3.42 | -0.41 | 6.36 | |  |  |  |  |  |  |  |  |
|  | ***qLP-Chr13-3*** | 2015E3 | NAU2893 | Gh157 |  |  |  | |  |  |  |  |  | 3.73 | -0.57 | 7.89 |
|  |  | 2016E1 | NAU2893 | Gh157 | 2.67 | -0.41 | 4.98 | |  |  |  |  |  |  |  |  |
|  |  | 2016E2 | NAU2893 | Gh157 |  |  |  | |  |  |  |  |  | 2.68 | -0.57 | 5.38 |
| *Cluster-Chr14-1* | ***qBNP-Chr14-2*** | 2015E2 | SWU14224 | DPL0565 |  |  |  | |  |  |  |  |  | 2.51 | 0.64 | 5.35 |
|  |  | 2015E3 | SWU14224 | DPL0565 |  |  |  | |  |  |  |  |  | 2.84 | 0.63 | 6.76 |
|  | *qLP-Chr14-3* | 2016E1 | SWU14224 | DPL0565 |  |  |  | |  |  |  |  |  | 3.52 | 0.74 | 7.89 |
| *Cluster-Chr19-1* | *qBNP-Chr19-2* | 2015E3 | NAU3437 | NAU2894 |  |  |  | |  |  |  |  |  | 3.66 | 0.74 | 7.44 |
|  | ***qLP-Chr19-1*** | 2015E2 | NAU3437 | NAU2894 | 2.88 | 0.37 | 5.68 | |  |  |  |  |  |  |  |  |
|  |  | 2016E1 | NAU3437 | NAU2894 |  |  |  | |  |  |  |  |  | 2.69 | 1.03 | 15.67 |
|  |  | 2016E2 | NAU3437 | NAU2894 |  |  |  | |  |  |  |  |  | 3.00 | 0.65 | 8.11 |
| *Cluster-Chr20-1* | ***qSY-Chr20-1*** | 2015E2 | SWU20501a | CER0167 |  |  |  | |  |  |  |  |  | 3.78 | -4.49 | 7.36 |
|  |  | 2016E1 | SWU20246 | SWU20501a |  |  |  | |  |  |  |  |  | 2.56 | -4.01 | 6.73 |
|  | *qLY-Chr20-1* | 2015E2 | SWU20501a | CER0167 |  |  |  | |  |  |  |  |  | 3.48 | -1.65 | 6.70 |
|  | *qBNP-Chr20-1* | 2015E2 | SWU20501a | CER0167 |  |  |  | |  |  |  |  |  | 4.35 | -0.87 | 8.94 |
|  | ***qBW-Chr20-1*** | 2015E2 | CER0167 | SWU20064 |  |  |  | |  |  |  |  |  | 2.76 | -0.11 | 5.52 |
|  |  | 2015E3 | SWU20501a | CER0167 |  |  |  | |  |  |  |  |  | 2.58 | -0.11 | 7.14 |
|  |  | 2016E1 | SWU20246 | SWU20501a | 3.77 | -0.11 | 7.46 | |  |  |  |  |  |  |  |  |
|  |  | 2016E2 | SWU20246 | SWU20501a |  |  |  | |  |  |  |  |  | 4.58 | -0.15 | 11.55 |
|  | *qLP-Chr20-1* | 2015E1 | CER0167 | SWU20064 |  |  |  | |  |  |  |  |  | 4.23 | -0.15 | 8.11 |
| *Cluster-Chr21-1* | ***qSY-Chr21-1*** | 2015E1 | CGR5808 | HAU0423 | 3.86 | 3.03 | 9.33 | |  |  |  |  |  |  |  |  |
|  |  | 2015E3 | CGR5808 | HAU0423 |  |  |  | |  |  |  |  |  | 4.58 | -4.19 | 9.20 |
|  | ***qSY-Chr21-2*** | 2015E1 | CGR5806 | DPL0777 | 2.63 | -2.66 | 6.96 | |  |  |  |  |  |  |  |  |
|  |  | 2016E1 | CGR5806 | DPL0777 |  |  |  | |  |  |  |  |  | 3.06 | -4.84 | 9.78 |
|  | ***qLY-Chr21-2*** | 2015E1 | HAU0423 | CGR5806 |  |  |  | |  | 3.72 | 1.03 | 8.02 |  |  |  |  |
|  |  | 2015E3 | HAU0423 | CGR5806 |  |  |  | |  |  |  |  |  | 4.92 | -1.78 | 9.82 |
|  |  | 2016E1 | CGR5806 | DPL0777 |  |  |  | |  |  |  |  |  | 3.65 | -2.40 | 16.15 |
|  |  | 2016E2 | CGR5806 | DPL0777 |  |  |  | |  |  |  |  |  | 3.29 | -2.53 | 16.66 |
|  | ***qBNP-Chr21-1*** | 2015E1 | CGR5808 | HAU0423 | 2.95 | 0.62 | 8.88 | |  |  |  |  |  |  |  |  |
|  |  | 2016E2 | HAU0423 | CGR5806 | 4.27 | -1.02 | 12.22 | |  |  |  |  |  |  |  |  |
|  | ***qLP-Chr21-2*** | 2016E2 | CGR5806 | DPL0777 |  |  |  | |  |  |  |  |  | 2.53 | -0.74 | 9.87 |
|  |  | 2016E2 | CGR5806 | DPL0777 | 3.08 | -0.45 | 8.70 | |  |  |  |  |  |  |  |  |
| *Cluster-Chr21-2* | *qLY-Chr21-3* | 2015E2 | BNL3442a | ICR08249 |  |  |  | |  |  |  |  |  | 3.88 | -1.78 | 7.52 |
|  | *qBNP-Chr21-3* | 2015E2 | BNL3442a | ICR08249 |  |  |  | |  |  |  |  |  | 3.41 | -0.78 | 6.90 |
| *Cluster-Chr23-1* | ***qBW-Chr23-1*** | 2015E2 | SWU14807 | PGML4185 | 2.88 | 0.08 | 5.60 | |  |  |  |  |  |  |  |  |
|  |  | 2015E2 | PGML4186 | NAU3100 | 2.52 | 0.08 | 5.61 | |  | 2.54 | 0.08 | 5.42 |  |  |  |  |
|  | *qLP-Chr23-1* | 2016E2 | Gh327 | SWU14770 |  |  |  | |  | 3.33 | 0.33 | 7.13 |  |  |  |  |
| *Cluster-Chr25-1* | *qSY-Chr25-1* | 2015E2 | NAU3112 | SWU19144 |  |  |  | |  | 3.66 | -3.62 | 7.41 |  |  |  |  |
|  | *qLY-Chr25-1* | 2015E2 | NAU3112 | SWU19144 |  |  |  | |  | 4.82 | -1.71 | 9.95 |  |  |  |  |

**Supplementary Table S6-A. Main effect QTLs and environmental interactions detected for yield and yield-component traits in RIL-M populations.** The result detected by software ICIMapping 4.1. E1, Cangzhou in 2015; E2, Wuhan in 2015; E3, Handan in 2015; E4, Cangzhou in 2016. Chr represented the linkage group number of the loci being tested in the analysis. V(A)% and V(AE)%: percentage of the total phenotypic variation explained by one QTL and by QTL × environmentat the current scanning position, respectively. Effect value: A, the total genetic effect of one QTL; AE, the effect value by QTL × environment; AE1, AE2, AE3 and AE4: the effect values under environments of E1, E2, E3 and E4, respectively. Bold figures indicated common QTLs between E-QTLs by QEs and M-QTLs by QEs in the population.

| **Trait** | **Chr** | **Left marker** | **Right marker** | **LOD** | **V(A)%** | **V(AE)%** | **Effect value** | | | | |
| --- | --- | --- | --- | --- | --- | --- | --- | --- | --- | --- | --- |
|  |  |  |  |  |  |  | **A** | **AE1** | **AE2** | **AE3** | **AE4** |
| SY | 2 | **SWU11887** | **SWU11976** | 8.40 | 2.85 | 1.47 | 2.13 | 1.97 | -1.76 | 0.99 | -1.20 |
|  | 5 | SWU20917 | NAU6240 | 4.17 | 1.99 | 0.35 | 1.79 | -0.18 | 0.19 | -1.05 | 1.04 |
|  | 6 | **ICR03206** | **NAU896** | 2.54 | 0.83 | 1.12 | 1.16 | -0.33 | -1.10 | -0.85 | 2.28 |
|  | 7 | **PGML1916** | **SWU10864** | 3.22 | 1.14 | 0.21 | 1.39 | -0.31 | 0.84 | 0.22 | -0.75 |
|  | 11 | **SWU15972** | TMB0628 | 2.99 | 1.09 | 0.85 | -1.32 | -1.40 | -0.89 | 0.97 | 1.32 |
|  | 14 | NAU2960 | ICR12130 | 2.91 | 0.01 | 1.34 | 0.10 | 1.17 | -0.85 | 1.64 | -1.96 |
|  | 18 | **SWU22290** | Gh501 | 3.01 | 1.61 | 0.44 | -1.61 | -0.39 | -0.19 | 1.40 | -0.82 |
|  | 18 | NAU748 | SWU22192 | 4.29 | 2.18 | 0.29 | -1.87 | -0.93 | -0.38 | 0.68 | 0.63 |
|  | 18 | SWU22192 | DPL0864 | 3.90 | 2.24 | 0.27 | -1.89 | -1.02 | 0.06 | 0.83 | 0.12 |
|  | 20 | **SWU20246** | **SWU20501a** | 3.01 | 1.24 | 0.83 | -1.41 | -0.83 | 0.74 | 1.47 | -1.38 |
|  | 20 | **DPL0319** | HAU1378 | 2.73 | 1.02 | 0.51 | -1.28 | 0.46 | -1.26 | 1.15 | -0.36 |
|  | 21 | HAU0423 | **CGR5806** | 4.94 | 1.88 | 1.27 | -1.76 | -0.78 | -1.01 | 2.49 | -0.71 |
|  | 22 | CAU0161 | NAU2026 | 2.78 | 0.81 | 0.22 | -1.23 | -0.03 | 0.22 | -0.98 | 0.79 |
|  | 24 | **SWU13267** | **BNL1521** | 3.42 | 1.68 | 0.50 | 1.64 | 1.47 | -0.95 | -0.33 | -0.19 |
|  | 26 | SWU18488 | SWU18672 | 3.22 | 1.30 | 0.60 | -1.44 | -0.03 | -1.54 | 1.14 | 0.44 |
|  | 27 | SWU10994 | HAU1001 | 2.95 | 1.16 | 0.24 | -1.36 | 0.07 | -0.93 | 0.02 | 0.83 |
|  | 31 | SWU16676 | SWU16755 | 2.73 | 1.45 | 0.19 | -1.52 | 0.42 | -0.47 | 0.67 | -0.62 |
| LY | 2 | SWU11887 | SWU11976 | 9.18 | 3.37 | 1.53 | 0.98 | 0.82 | -0.76 | 0.46 | -0.52 |
|  | 7 | PGML1916 | SWU10864 | 2.76 | 1.03 | 0.10 | 0.58 | -0.18 | 0.05 | 0.27 | -0.14 |
|  | 11 | SWU15972 | TMB0628 | 3.24 | 0.94 | 1.04 | -0.52 | -0.64 | -0.42 | 0.42 | 0.64 |
|  | 14 | SWU14224 | DPL0565 | 2.83 | 1.31 | 0.47 | 0.61 | 0.59 | 0.01 | -0.27 | -0.33 |
|  | 18 | Gh60 | SWU22281 | 2.92 | 1.59 | 0.47 | -0.67 | -0.16 | 0.01 | 0.57 | -0.43 |
|  | 18 | SWU22192 | DPL0864 | 3.56 | 2.06 | 0.21 | -0.76 | -0.34 | 0.08 | 0.34 | -0.07 |
|  | 21 | HAU0423 | CGR5806 | 4.68 | 1.68 | 1.21 | -0.70 | -0.33 | -0.42 | 1.02 | -0.27 |
|  | 24 | SWU13267 | BNL1521 | 3.38 | 1.66 | 0.40 | 0.69 | 0.54 | -0.38 | -0.10 | -0.06 |
|  | 25 | CGR6864 | SWU19815 | 3.26 | 1.75 | 0.40 | -0.70 | -0.43 | -0.06 | 0.51 | -0.02 |
|  | 25 | SWU19430 | PGML1219 | 3.29 | 1.53 | 0.98 | -0.66 | 0.01 | 0.15 | 0.65 | -0.81 |
|  | 26 | SWU18488 | SWU18672 | 3.47 | 1.33 | 0.65 | -0.61 | 0.02 | -0.66 | 0.53 | 0.11 |
|  | 31 | SWU16676 | SWU16755 | 2.82 | 1.48 | 0.21 | -0.65 | 0.20 | -0.21 | 0.29 | -0.27 |
| BNP | 2 | SWU11887 | SWU11976 | 4.65 | 0.79 | 1.67 | 0.23 | 0.47 | -0.41 | 0.16 | -0.22 |
|  | 5 | TMB1296 | HAU1603 | 2.90 | 0.05 | 1.36 | -0.06 | 0.03 | -0.38 | 0.47 | -0.12 |
|  | 7 | NAU1357 | **SWU10067** | 2.93 | 0.76 | 0.65 | -0.23 | 0.36 | -0.14 | -0.18 | -0.04 |
|  | 8 | SHIN1341 | PGML2605 | 3.16 | 1.23 | 0.44 | -0.29 | 0.29 | 0.00 | -0.14 | -0.15 |
|  | 11 | ICR08245 | BNL3442b | 2.60 | 0.05 | 1.01 | 0.06 | -0.06 | -0.30 | 0.43 | -0.06 |
|  | 14 | SWU14224 | DPL0565 | 3.92 | 1.77 | 0.39 | 0.35 | 0.23 | 0.07 | -0.10 | -0.20 |
|  | 16 | DPL0048 | **SWU10266** | 2.79 | 1.56 | 0.19 | 0.33 | -0.09 | 0.00 | -0.10 | 0.19 |
|  | 16 | **SWU10214** | **Gh56** | 2.56 | 0.13 | 1.24 | -0.10 | 0.51 | -0.20 | -0.14 | -0.17 |
|  | 17 | HAU1413 | CGR6017 | 2.55 | 1.31 | 0.32 | -0.30 | -0.07 | 0.14 | 0.14 | -0.21 |
|  | 19 | **NAU3437** | **NAU2894** | 3.30 | 0.48 | 1.55 | 0.18 | -0.32 | 0.55 | -0.10 | -0.12 |
|  | 19 | PGML4342 | SWU14431b | 2.69 | 1.15 | 0.82 | -0.28 | 0.19 | -0.06 | 0.24 | -0.36 |
|  | 21 | JESPR154 | SWU14431a | 2.50 | 0.81 | 0.68 | 0.24 | -0.05 | 0.38 | -0.17 | -0.16 |
|  | 22 | CAU0161 | NAU2026 | 2.59 | 0.40 | 0.65 | -0.18 | -0.03 | 0.19 | -0.36 | 0.21 |
|  | 23 | Gh327 | SWU14770 | 3.54 | 1.55 | 0.48 | -0.33 | 0.26 | 0.05 | -0.08 | -0.23 |
|  | 25 | SWU19430 | PGML1219 | 3.15 | 1.27 | 0.93 | -0.30 | -0.04 | 0.02 | 0.37 | -0.35 |
|  | 27 | **CGR6857** | **ICR11205** | 2.96 | 1.09 | 0.53 | 0.28 | -0.17 | 0.32 | -0.02 | -0.13 |
| BW | 2 | SWU11887 | SWU11976 | 3.70 | 1.75 | 0.15 | 0.06 | 0.00 | -0.03 | 0.01 | 0.01 |
|  | 4 | SWU21415 | BNL530 | 5.85 | 3.13 | 0.09 | 0.08 | 0.01 | -0.01 | 0.02 | -0.01 |
|  | 5 | SWU20917 | **NAU6240** | 9.59 | 4.81 | 0.66 | 0.10 | 0.01 | 0.02 | 0.04 | -0.06 |
|  | 5 | HAU1603 | PGML4457 | 2.79 | 0.66 | 1.17 | 0.04 | -0.03 | -0.03 | 0.08 | -0.03 |
|  | 9 | Gh111 | Gh27 | 3.00 | 1.58 | 0.08 | -0.06 | 0.01 | 0.01 | -0.02 | 0.00 |
|  | 14 | CGR6784 | **NAU874** | 2.85 | 1.37 | 0.31 | -0.05 | -0.01 | 0.03 | -0.04 | 0.02 |
|  | 14 | NAU2960 | ICR12130 | 3.00 | 1.24 | 0.31 | -0.05 | 0.01 | -0.04 | 0.02 | 0.01 |
|  | 16 | SWU10094 | SWU10060 | 3.85 | 1.55 | 0.27 | 0.06 | -0.01 | -0.03 | 0.01 | 0.03 |
|  | 16 | **SWU10038** | **ICR00016** | 2.71 | 1.05 | 0.31 | 0.05 | 0.01 | -0.04 | 0.00 | 0.03 |
|  | 17 | CGR5871 | SWU12876 | 2.74 | 1.39 | 0.16 | 0.05 | 0.01 | -0.02 | 0.02 | -0.01 |
|  | 19 | **SWU17897** | **CGR5539** | 3.47 | 1.65 | 0.10 | -0.06 | 0.01 | 0.00 | 0.01 | -0.02 |
|  | 20 | **SWU20501a** | **CER0167** | 12.00 | 6.44 | 0.33 | -0.11 | 0.03 | 0.02 | -0.03 | -0.02 |
|  | 21 | CGR5808 | HAU0423 | 5.69 | 2.98 | 0.05 | -0.08 | 0.00 | 0.01 | 0.01 | -0.01 |
|  | 22 | SWU21646 | SWU21585 | 5.29 | 2.08 | 0.62 | 0.06 | -0.03 | -0.04 | 0.02 | 0.05 |
|  | 23 | Gh327 | SWU14770 | 3.47 | 1.73 | 0.22 | 0.06 | 0.02 | -0.03 | 0.02 | -0.01 |
|  | 24 | SWU13150 | PGML4657 | 2.81 | 1.32 | 0.16 | -0.05 | 0.00 | -0.03 | 0.02 | 0.01 |
|  | 24 | **HAU2504** | SWU13736 | 3.44 | 1.58 | 0.18 | -0.06 | 0.00 | 0.03 | 0.00 | -0.02 |
|  | 26 | NAU2175 | SWU17336 | 3.41 | 1.10 | 0.68 | 0.05 | 0.03 | 0.05 | -0.04 | -0.03 |
|  | 26 | **SWU18672** | SWU18681 | 4.13 | 2.03 | 0.11 | 0.06 | -0.02 | 0.00 | 0.00 | 0.02 |
|  | 29 | **C2_0115** | **ICR03107** | 2.61 | 1.25 | 0.14 | 0.05 | 0.02 | -0.02 | 0.00 | 0.00 |
|  | 30 | **BNL243** | **CER0168** | 4.11 | 2.01 | 0.17 | -0.06 | 0.02 | -0.03 | -0.01 | 0.02 |
|  | 31 | SWU16676 | SWU16755 | 2.63 | 0.93 | 0.46 | -0.04 | 0.04 | 0.02 | -0.03 | -0.03 |
| LP | 2 | SWU12025 | **SWU11889** | 4.36 | 1.98 | 0.21 | 0.31 | -0.02 | -0.09 | 0.17 | -0.06 |
|  | 3 | **SWU12819** | **SWU12765** | 5.99 | 2.99 | 0.06 | 0.38 | 0.04 | -0.03 | -0.07 | 0.06 |
|  | 4 | SWU18881 | NAU2701 | 3.15 | 1.43 | 0.12 | -0.26 | -0.01 | -0.11 | 0.10 | 0.01 |
|  | 5 | **SWU20917** | **NAU6240** | 24.52 | 13.13 | 0.10 | -0.79 | 0.04 | 0.07 | 0.00 | -0.11 |
|  | 5 | **TMB1296** | **HAU1603** | 3.89 | 1.86 | 0.15 | -0.30 | -0.07 | 0.14 | 0.00 | -0.06 |
|  | 6 | **BNL3650** | ICR10602 | 2.86 | 1.30 | 0.16 | -0.25 | 0.04 | 0.07 | -0.15 | 0.04 |
|  | 7 | **SWU10064** | **NAU3181** | 5.79 | 2.90 | 0.01 | -0.37 | 0.04 | -0.01 | -0.03 | -0.01 |
|  | 11 | BNL3442b | **ICR01810** | 3.16 | 1.45 | 0.09 | 0.26 | -0.06 | 0.07 | 0.06 | -0.07 |
|  | 13 | **NAU2893** | **Gh157** | 13.10 | 6.88 | 0.15 | -0.59 | -0.04 | 0.13 | -0.10 | 0.01 |
|  | 13 | **BNL1495** | **CGR5390** | 3.17 | 1.49 | 0.08 | -0.27 | -0.02 | -0.08 | 0.10 | 0.00 |
|  | 14 | NAU3308 | HAU1057 | 6.36 | 3.06 | 0.21 | 0.38 | 0.04 | -0.09 | 0.14 | -0.09 |
|  | 14 | Gh120 | PGML1884 | 2.93 | 1.38 | 0.15 | 0.26 | 0.06 | -0.02 | -0.13 | 0.09 |
|  | 14 | NAU3820 | **NAU2960** | 2.75 | 1.16 | 0.19 | 0.23 | -0.12 | 0.14 | -0.03 | 0.01 |
|  | 19 | **NAU3437** | **NAU2894** | 3.63 | 1.66 | 0.22 | 0.28 | -0.01 | 0.02 | -0.15 | 0.14 |
|  | 20 | **CER0167** | SWU20064 | 3.07 | 1.22 | 0.22 | 0.24 | -0.06 | 0.13 | 0.06 | -0.13 |
|  | 22 | **SWU21635** | **SWU21646** | 2.57 | 1.16 | 0.16 | 0.23 | -0.07 | 0.10 | -0.10 | 0.07 |
|  | 22 | **PGML1712** | SWU21538 | 4.13 | 2.01 | 0.07 | 0.31 | -0.04 | -0.06 | 0.09 | 0.01 |
|  | 22 | **DPL0562** | **CAU0161** | 6.21 | 3.12 | 0.02 | 0.40 | -0.03 | -0.03 | 0.05 | 0.02 |
|  | 25 | HAU1382 | **SWU19848** | 2.72 | 1.31 | 0.11 | -0.25 | -0.08 | 0.11 | 0.02 | -0.04 |
|  | 25 | **CGR6864** | SWU19815 | 4.87 | 2.45 | 0.07 | -0.34 | 0.06 | 0.05 | -0.01 | -0.09 |
|  | 25 | Gh220 | SWU19434 | 5.13 | 2.53 | 0.14 | -0.35 | -0.01 | 0.02 | 0.11 | -0.12 |
|  | 26 | **SWU17251** | **C2_0135** | 3.32 | 1.65 | 0.01 | 0.28 | 0.01 | 0.03 | 0.00 | -0.03 |
|  | 27 | **SWU11038** | **SWU11384** | 3.04 | 1.44 | 0.14 | -0.26 | 0.07 | -0.04 | 0.08 | -0.11 |
|  | 27 | CGR5056 | DC40052 | 2.66 | 1.31 | 0.04 | -0.25 | 0.08 | -0.04 | -0.03 | -0.01 |
|  | 28 | **SWU12343** | **SWU14060** | 2.91 | 0.95 | 0.49 | 0.21 | 0.11 | -0.09 | -0.20 | 0.18 |

**Supplementary Table S6-B. Epistatic QTLs and environmental interactions detected for yield and yield-component traits in RIL-M populations.** See footnotes in Table S2. Ch*i* and Ch*j* represented the linkage group number of the loci being tested in the analysis. V(AA)% and V(AAE)%: percentage of the total phenotypic variation, explaining by epistatic QTLs and by epistatic QTLs × the environment, respectively. Effect value: AA, the total effect value of epistatic QTLs; AAEl, AAE2, AAE3 and AAE4 indicated the effects by epistatic QTLs × environment in E1, E2, E3 and E4, respectively.

| **Trait** | **Ch*i*** | **Left marker** | **Right marker** | **Ch*j*** | **Left marker** | **Right marker** | **LOD** | **V(AA)%** | **V(AAE)%** | **Effect value** | | | | |
| --- | --- | --- | --- | --- | --- | --- | --- | --- | --- | --- | --- | --- | --- | --- |
|  |  |  |  |  |  |  |  |  |  | **AA** | **AAE1** | **AAE2** | **AAE3** | **AAE4** |
| SY | 1 | SWU10986 | NAU2218 | 2 | **SWU11887** | **SWU11976** | 5.50 | 2.21 | 0.31 | -1.89 | -0.06 | -0.03 | 1.02 | -0.93 |
|  | 1 | HAU1417 | NAU2437 | 3 | HAU2424 | CER0028 | 6.07 | 2.56 | 0.64 | 2.03 | -0.05 | -0.70 | -0.93 | 1.67 |
|  | 5 | SWU13378 | SWU17846 | 9 | NAU3966 | SWU15157 | 5.46 | 2.47 | 0.32 | -2.01 | -0.78 | 0.20 | 1.10 | -0.52 |
|  | 9 | Gh158 | DC40407 | 10 | CGR5873 | ICR00093 | 5.45 | 1.84 | 0.94 | 1.75 | 1.28 | -1.12 | -1.36 | 1.19 |
|  | 7 | **PGML1916** | **SWU10864** | 10 | ICR00093 | ICR07050 | 6.12 | 1.71 | 1.45 | -1.87 | -2.93 | 1.23 | 0.73 | 0.96 |
|  | 2 | SWU12393 | SWU11013 | 10 | Gh320 | HAU0635 | 5.24 | 1.27 | 1.09 | 1.43 | 0.56 | -0.78 | -1.62 | 1.83 |
|  | 7 | SWU10067 | SWU10064 | 11 | PGML2202 | CGR6580 | 7.92 | 3.48 | 0.47 | 2.38 | -0.23 | 1.12 | -1.26 | 0.37 |
|  | 5 | SWU17787 | SWU13378 | 11 | NAU1014 | ICR10344 | 5.06 | 2.06 | 0.61 | 1.85 | 1.04 | -1.17 | -0.78 | 0.91 |
|  | 8 | HAU1470a | SHIN1341 | 12 | DPL0303 | COT107 | 6.08 | 2.52 | 0.66 | -2.01 | -0.51 | -1.42 | 0.86 | 1.08 |
|  | 7 | CGR5001 | CGR6586 | 13 | NAU3468 | SWU22309 | 5.17 | 1.34 | 0.85 | 1.49 | 1.28 | 0.87 | -1.81 | -0.34 |
|  | 3 | SWU12732 | SWU12783 | 14 | SWU14224 | DPL0565 | 5.50 | 2.68 | 0.33 | 2.08 | 0.81 | -0.54 | -0.89 | 0.62 |
|  | 10 | SWU20501b | CGR5873 | 14 | SWU14545 | SWU14543 | 5.37 | 1.90 | 0.77 | 1.76 | 1.20 | -1.44 | -0.79 | 1.04 |
|  | 2 | SWU12126 | SWU12147 | 16 | PGML1709 | SWU10627 | 5.83 | 2.06 | 0.97 | -1.82 | -0.97 | 1.01 | 1.43 | -1.47 |
|  | 9 | PGML2830 | CGR6876 | 16 | DPL0048 | SWU10266 | 6.44 | 1.94 | 1.15 | 1.78 | 0.26 | -0.83 | -1.52 | 2.09 |
|  | 5 | SWU20913 | Gh260 | 16 | SWU10266 | DC40065 | 5.11 | 1.98 | 0.60 | -1.79 | -1.39 | -0.22 | 1.31 | 0.31 |
|  | 11 | CGR6580 | **SWU15972** | 16 | SWU10038 | ICR00016 | 6.18 | 3.27 | 0.12 | 2.39 | 0.45 | 0.02 | -0.73 | 0.26 |
|  | 16 | ICR00010 | SWU10038 | 17 | ICR03391 | SWU12838a | 6.36 | 2.61 | 0.65 | 2.05 | 1.76 | -0.59 | -0.60 | -0.57 |
|  | 6 | CGR5108 | **ICR03206** | 17 | CGR5576 | NAU3765 | 7.04 | 3.32 | 0.10 | 2.36 | 0.68 | 0.05 | -0.60 | -0.13 |
|  | 1 | SWU11191 | BNL2827b | 17 | CGR5576 | NAU3765 | 5.07 | 2.24 | 0.64 | -1.95 | -1.75 | 0.89 | 0.35 | 0.51 |
|  | 14 | PGML1568 | Gh529 | 18 | SWU22287 | **SWU22290** | 5.04 | 1.49 | 1.24 | -1.57 | 0.48 | 0.84 | 1.10 | -2.42 |
|  | 3 | SWU12732 | SWU12783 | 19 | SWU17782 | DPL0056 | 6.06 | 2.80 | 0.45 | 2.13 | 1.15 | -0.70 | -0.94 | 0.49 |
|  | 7 | NAU1357 | SWU10067 | 20 | CGR5548 | SWU20675 | 5.40 | 2.05 | 0.71 | -1.83 | -1.72 | 0.05 | 1.23 | 0.44 |
|  | 14 | PGML4763 | SWU13909 | 20 | SWU20636 | CGR6154 | 5.68 | 2.15 | 0.86 | 1.88 | 1.13 | -0.34 | -1.80 | 1.00 |
|  | 16 | NAU2984 | SWU10062 | 20 | **SWU20246** | **SWU20501a** | 5.20 | 2.31 | 0.14 | 1.95 | 0.40 | 0.45 | -0.75 | -0.10 |
|  | 20 | SWU20649 | SWU20636 | 20 | SWU1259 | SWU20033 | 5.23 | 2.26 | 0.71 | -1.92 | -1.74 | 0.67 | 1.07 | 0.00 |
|  | 10 | BNL2960 | SWU20511 | 20 | SWU20035 | **DPL0319** | 5.33 | 1.57 | 0.88 | 1.61 | 1.08 | 0.19 | -1.97 | 0.71 |
|  | 6 | **NAU896** | BNL3650 | 21 | SWU0830 | HAU2004 | 5.01 | 1.79 | 0.88 | -1.75 | -1.42 | 1.38 | 1.08 | -1.04 |
|  | 7 | Gh474 | SWU10785 | 21 | **CGR5806** | DPL0777 | 5.71 | 1.27 | 1.02 | 1.43 | -0.37 | 1.32 | -1.93 | 0.98 |
|  | 19 | NAU3437 | NAU2894 | 22 | SWU21635 | SWU21646 | 5.04 | 2.25 | 0.31 | 1.93 | -0.61 | 0.17 | -0.78 | 1.22 |
|  | 7 | CGR5001 | CGR6586 | 22 | SWU21646 | SWU21585 | 6.91 | 2.96 | 0.37 | 2.18 | 0.40 | 0.09 | -1.27 | 0.78 |
|  | 19 | NAU3437 | NAU2894 | 24 | **BNL1521** | HAU2504 | 6.41 | 2.27 | 0.67 | -1.93 | -1.14 | 0.65 | 1.40 | -0.91 |
|  | 5 | PGML1917 | SWU17715 | 24 | SWU13745 | Gh273 | 5.09 | 2.24 | 0.50 | 1.93 | 1.52 | -0.27 | -0.94 | -0.30 |
|  | 2 | PGML0700 | SWU12016 | 25 | HAU1382 | SWU19848 | 5.88 | 2.17 | 0.54 | 1.88 | 0.76 | 0.04 | -1.60 | 0.80 |
|  | 11 | PGML2202 | CGR6580 | 25 | SWU19815 | BNL3594 | 8.08 | 2.97 | 1.24 | 2.19 | 1.35 | -1.54 | -1.28 | 1.46 |
|  | 16 | SWU10062 | SWU10094 | 26 | NAU2175 | SWU17336 | 5.08 | 1.73 | 0.88 | -1.69 | -2.03 | 0.50 | 1.10 | 0.42 |
|  | 24 | **SWU13267** | **BNL1521** | 26 | SWU17336 | NAU5072 | 6.33 | 2.48 | 0.49 | 2.06 | 0.42 | -0.40 | -1.25 | 1.24 |
|  | 18 | DC40150 | ICR02849 | 26 | DPL0491 | Gh64 | 5.09 | 2.68 | 0.23 | -2.12 | -0.91 | 0.30 | 0.37 | 0.24 |
|  | 13 | NAU2893 | Gh157 | 26 | Gh64 | SWU17257 | 5.52 | 2.63 | 0.23 | -2.08 | -0.59 | -0.42 | 1.06 | -0.05 |
|  | 2 | PGML0700 | SWU12016 | 26 | C2_0135 | PGML2321 | 5.38 | 2.48 | 0.28 | -1.99 | 0.29 | 0.32 | 0.50 | -1.12 |
|  | 6 | CGR5801 | SWU19249 | 26 | C2_0135 | PGML2321 | 5.81 | 2.70 | 0.16 | -2.11 | -0.27 | 0.43 | 0.41 | -0.57 |
|  | 9 | PGML2830 | CGR6876 | 26 | SWU18681 | SWU0598 | 9.25 | 3.83 | 0.47 | -2.48 | -1.32 | -0.04 | 1.05 | 0.30 |
|  | 6 | **NAU896** | BNL3650 | 27 | SWU11038 | SWU11384 | 5.24 | 1.85 | 0.74 | 1.72 | 1.90 | -0.61 | -0.79 | -0.51 |
|  | 19 | TMB0107 | NAU3217 | 27 | SWU11384 | ICR11885 | 5.67 | 1.85 | 1.31 | -1.76 | -0.46 | 1.74 | 0.90 | -2.18 |
|  | 21 | SWU16651 | SWU16645 | 27 | SWU11384 | ICR11885 | 5.04 | 1.14 | 1.66 | -1.37 | -1.22 | 1.34 | 1.92 | -2.04 |
|  | 1 | ICR03724 | ICR03725 | 27 | ICR11883 | CGR6356 | 6.32 | 3.08 | 0.28 | -2.24 | -0.75 | -0.07 | 1.08 | -0.27 |
|  | 20 | SWU20675 | SWU20649 | 27 | ICR11883 | CGR6356 | 5.72 | 2.25 | 0.63 | -1.96 | -1.20 | 0.06 | 1.62 | -0.48 |
|  | 11 | NAU1014 | ICR10344 | 28 | BNL2877 | HAU3071 | 5.74 | 2.49 | 0.72 | 1.99 | 1.56 | -1.14 | -0.86 | 0.45 |
|  | 13 | SWU22374 | HAU2857 | 28 | BNL2877 | HAU3071 | 5.22 | 2.10 | 0.69 | -1.89 | -0.54 | -0.03 | 1.72 | -1.15 |
|  | 2 | SWU12126 | SWU12147 | 28 | SHIN0219 | TMB2386 | 5.47 | 2.62 | 0.35 | 2.05 | 0.61 | 0.85 | -0.96 | -0.51 |
|  | 25 | BNL3594 | DPL0282 | 28 | SHIN0219 | TMB2386 | 6.81 | 2.87 | 0.72 | 2.15 | -0.23 | -0.82 | -0.77 | 1.82 |
|  | 27 | ICR11205 | DPL0847a | 28 | SHIN0219 | TMB2386 | 5.07 | 1.90 | 0.90 | -1.77 | 0.66 | -0.17 | 1.37 | -1.87 |
|  | 20 | SWU20636 | CGR6154 | 28 | NBRI0014 | SWU12107 | 5.77 | 2.75 | 0.26 | 2.13 | 0.80 | -0.13 | -1.02 | 0.35 |
|  | 28 | NBRI0014 | SWU12107 | 29 | BNL3261 | CGR5111 | 5.95 | 3.10 | 0.15 | 2.24 | 0.41 | -0.30 | -0.66 | 0.54 |
|  | 7 | SWU10067 | SWU10064 | 29 | BNL3261 | CGR5111 | 5.20 | 2.20 | 0.72 | 1.90 | 1.65 | -1.13 | 0.28 | -0.81 |
|  | 20 | SWU20035 | **DPL0319** | 29 | BNL3261 | CGR5111 | 6.98 | 2.86 | 0.45 | 2.14 | 0.79 | -0.39 | -1.23 | 0.83 |
|  | 25 | HAU1382 | SWU19848 | 29 | C2_0115 | ICR03107 | 5.82 | 1.35 | 1.55 | 1.47 | -0.34 | -0.27 | -1.88 | 2.50 |
|  | 26 | SWU17251 | C2_0135 | 29 | C2_0115 | ICR03107 | 5.35 | 2.38 | 0.26 | -1.96 | -0.67 | -0.19 | 1.07 | -0.21 |
|  | 16 | SWU10266 | DC40065 | 30 | CER0168 | SWU21718 | 5.75 | 2.48 | 0.68 | 2.00 | 0.33 | -0.89 | -0.94 | 1.51 |
|  | 28 | SHIN0219 | TMB2386 | 30 | CER0168 | SWU21718 | 5.06 | 2.48 | 0.31 | 2.00 | 0.93 | 0.02 | -1.16 | 0.21 |
| LY | 1 | HAU1417 | NAU2437 | 3 | HAU2424 | CER0028 | 5.29 | 2.15 | 0.70 | 0.78 | -0.05 | -0.20 | -0.48 | 0.73 |
|  | 1 | NAU3384 | CGR5663 | 4 | ICR01729 | SWU16781 | 5.14 | 2.36 | 0.28 | -0.82 | -0.01 | -0.03 | 0.42 | -0.38 |
|  | 4 | BNL530 | SWU21485 | 6 | HAU1460 | HAU1371 | 6.86 | 2.04 | 0.99 | -0.76 | -0.07 | -0.59 | 0.86 | -0.21 |
|  | 1 | CGR6129 | DPL0790 | 8 | HAU3177 | NAU4064 | 6.15 | 2.75 | 0.77 | 0.89 | -0.52 | 0.25 | -0.37 | 0.64 |
|  | 5 | DPL0022 | SWU17787 | 8 | DC20094 | HAU1470b | 5.60 | 1.60 | 1.27 | 0.68 | -0.01 | -0.24 | -0.70 | 0.95 |
|  | 5 | SWU13378 | SWU17846 | 9 | NAU3966 | SWU15157 | 6.31 | 2.98 | 0.28 | -0.93 | -0.30 | -0.13 | 0.47 | -0.04 |
|  | 7 | **PGML1916** | **SWU10864** | 10 | ICR00093 | ICR07050 | 5.91 | 1.94 | 1.15 | -0.85 | -1.08 | 0.31 | 0.23 | 0.54 |
|  | 7 | NAU1357 | SWU10067 | 11 | PGML2202 | CGR6580 | 5.90 | 2.18 | 0.38 | 0.79 | -0.03 | 0.31 | -0.53 | 0.25 |
|  | 5 | SWU17787 | SWU13378 | 11 | NAU1014 | ICR10344 | 5.61 | 2.47 | 0.53 | 0.85 | 0.33 | -0.38 | -0.38 | 0.43 |
|  | 8 | HAU1470a | SHIN1341 | 12 | DPL0303 | COT107 | 5.49 | 2.15 | 0.73 | -0.78 | -0.24 | -0.62 | 0.51 | 0.35 |
|  | 3 | SWU12732 | SWU12783 | 14 | **SWU14224** | **DPL0565** | 5.23 | 2.29 | 0.32 | 0.81 | 0.30 | 0.09 | -0.51 | 0.12 |
|  | 9 | PGML2830 | CGR6876 | 16 | DPL0048 | SWU10266 | 6.43 | 1.84 | 1.31 | 0.73 | 0.07 | -0.37 | -0.67 | 0.97 |
|  | 2 | PGML0700 | SWU12016 | 16 | NAU862 | CGR6802 | 6.31 | 1.80 | 1.14 | 0.72 | 0.69 | -0.88 | -0.04 | 0.22 |
|  | 11 | CGR6580 | **SWU15972** | 16 | SWU10038 | ICR00016 | 5.20 | 2.73 | 0.15 | 0.92 | 0.20 | -0.05 | -0.32 | 0.17 |
|  | 6 | CGR5108 | ICR03206 | 17 | CGR5576 | NAU3765 | 6.33 | 2.93 | 0.15 | 0.93 | 0.28 | 0.14 | -0.34 | -0.08 |
|  | 1 | SWU11191 | BNL2827b | 17 | CGR5576 | NAU3765 | 5.07 | 2.35 | 0.52 | -0.84 | -0.64 | 0.38 | 0.22 | 0.05 |
|  | 3 | SWU12732 | SWU12783 | 19 | SWU17782 | DPL0056 | 6.32 | 2.76 | 0.36 | 0.89 | 0.40 | -0.28 | -0.35 | 0.23 |
|  | 10 | BNL2960 | SWU20511 | 20 | SWU20035 | DPL0319 | 5.45 | 1.37 | 1.12 | 0.63 | 0.46 | 0.00 | -0.92 | 0.46 |
|  | 18 | **Gh60** | **SWU22281** | 20 | SWU20035 | DPL0319 | 5.23 | 2.19 | 0.65 | 0.79 | 0.27 | -0.39 | -0.45 | 0.57 |
|  | 9 | NAU1282 | CGR6771 | 21 | Gh451 | SWU16489 | 5.12 | 1.13 | 1.75 | -0.58 | -0.83 | 0.72 | 0.65 | -0.54 |
|  | 6 | NAU896 | BNL3650 | 21 | SWU0830 | HAU2004 | 5.68 | 2.19 | 0.41 | -0.81 | -0.48 | 0.38 | 0.36 | -0.25 |
|  | 20 | SWU20035 | DPL0319 | 21 | SWU14431a | SWU15915 | 5.31 | 2.16 | 0.64 | 0.80 | 0.65 | -0.25 | -0.45 | 0.05 |
|  | 7 | Gh474 | SWU10785 | 21 | **CGR5806** | DPL0777 | 5.76 | 1.37 | 1.08 | 0.62 | -0.19 | 0.51 | -0.82 | 0.50 |
|  | 8 | CGR6508 | Gh197 | 21 | CGR5217 | BNL3442a | 6.55 | 2.89 | 0.48 | 0.91 | 0.50 | 0.06 | -0.56 | 0.00 |
|  | 7 | CGR5001 | CGR6586 | 22 | SWU21646 | SWU21585 | 7.40 | 3.29 | 0.39 | 0.97 | 0.05 | 0.18 | -0.55 | 0.32 |
|  | 14 | NAU874 | SWU13824 | 22 | SWU21586 | PGML1712 | 5.14 | 2.23 | 0.60 | 0.81 | -0.26 | -0.14 | -0.32 | 0.72 |
|  | 13 | SWU22413 | CGR5331 | 23 | SWU14807 | PGML4185 | 5.29 | 2.73 | 0.42 | -0.89 | -0.08 | 0.36 | 0.25 | -0.54 |
|  | 17 | SWU12818 | CGR5576 | 24 | SWU13150 | PGML4657 | 5.36 | 1.01 | 1.80 | 0.55 | 0.93 | -0.96 | -0.39 | 0.43 |
|  | 2 | SWU12126 | SWU12147 | 24 | **BNL1521** | HAU2504 | 5.25 | 1.53 | 0.99 | -0.66 | -0.76 | 0.47 | 0.53 | -0.23 |
|  | 19 | NAU3437 | NAU2894 | 24 | **BNL1521** | HAU2504 | 5.91 | 2.07 | 0.69 | -0.77 | -0.45 | 0.30 | 0.58 | -0.43 |
|  | 5 | PGML1917 | SWU17715 | 24 | SWU13745 | Gh273 | 5.35 | 2.39 | 0.47 | 0.84 | 0.57 | 0.06 | -0.43 | -0.21 |
|  | 2 | PGML0700 | SWU12016 | 25 | HAU1382 | SWU19848 | 5.91 | 2.16 | 0.62 | 0.79 | 0.24 | -0.08 | -0.67 | 0.51 |
|  | 11 | PGML2202 | CGR6580 | 25 | **SWU19815** | BNL3594 | 5.68 | 2.14 | 0.91 | 0.78 | 0.47 | -0.58 | -0.43 | 0.54 |
|  | 5 | SWU20917 | NAU6240 | 25 | DPL0282 | SWU19763 | 5.25 | 1.91 | 0.47 | 0.74 | 0.00 | 0.36 | -0.59 | 0.23 |
|  | 24 | PGML4657 | Gh454 | 26 | SWU17336 | NAU5072 | 7.56 | 3.09 | 0.40 | 0.96 | -0.15 | 0.03 | -0.41 | 0.53 |
|  | 13 | SWU22413 | CGR5331 | 26 | SWU17251 | C2_0135 | 5.78 | 2.08 | 0.67 | -0.77 | -0.24 | -0.59 | 0.49 | 0.34 |
|  | 18 | DC40150 | ICR02849 | 26 | SWU17251 | C2_0135 | 5.84 | 2.76 | 0.37 | -0.94 | -0.07 | 0.14 | 0.44 | -0.51 |
|  | 26 | DPL0491 | Gh64 | 26 | SWU17251 | C2_0135 | 5.07 | 1.37 | 1.09 | -0.66 | 0.15 | -0.28 | 0.89 | -0.77 |
|  | 9 | PGML2830 | CGR6876 | 26 | SWU18681 | SWU0598 | 7.62 | 3.06 | 0.40 | -0.93 | -0.46 | -0.09 | 0.46 | 0.09 |
|  | 14 | PGML1368 | PGML1568 | 27 | SWU11038 | SWU11384 | 5.43 | 1.68 | 0.74 | -0.69 | 0.34 | -0.40 | 0.57 | -0.50 |
|  | 19 | TMB0107 | NAU3217 | 27 | SWU11384 | ICR11885 | 6.10 | 2.41 | 1.15 | -0.85 | -0.11 | 0.58 | 0.43 | -0.91 |
|  | 1 | ICR03724 | ICR03725 | 27 | ICR11883 | CGR6356 | 6.28 | 2.98 | 0.36 | -0.92 | -0.24 | -0.02 | 0.53 | -0.27 |
|  | 20 | SWU20675 | SWU20649 | 27 | ICR11883 | CGR6356 | 6.58 | 2.13 | 1.01 | -0.81 | -0.59 | -0.03 | 0.87 | -0.26 |
|  | 11 | NAU1014 | ICR10344 | 28 | BNL2877 | HAU3071 | 5.90 | 2.69 | 0.60 | 0.87 | 0.60 | -0.38 | -0.40 | 0.18 |
|  | 13 | SWU22374 | HAU2857 | 28 | BNL2877 | HAU3071 | 5.44 | 2.20 | 0.71 | -0.82 | -0.22 | -0.04 | 0.74 | -0.49 |
|  | 2 | SWU12126 | SWU12147 | 28 | SHIN0219 | TMB2386 | 6.54 | 2.91 | 0.60 | 0.91 | 0.20 | 0.56 | -0.53 | -0.23 |
|  | 25 | BNL3594 | DPL0282 | 28 | SHIN0219 | TMB2386 | 6.93 | 3.17 | 0.59 | 0.95 | -0.12 | -0.26 | -0.31 | 0.70 |
|  | 28 | TMB2386 | SWU12343 | 29 | BNL3261 | CGR5111 | 6.40 | 3.10 | 0.25 | 0.94 | 0.04 | -0.11 | -0.33 | 0.40 |
|  | 20 | SWU20035 | DPL0319 | 29 | BNL3261 | CGR5111 | 6.99 | 2.99 | 0.43 | 0.92 | 0.18 | -0.13 | -0.50 | 0.44 |
|  | 25 | HAU1382 | SWU19848 | 29 | C2_0115 | ICR03107 | 5.96 | 1.47 | 1.54 | 0.65 | -0.21 | 0.07 | -0.85 | 0.99 |
|  | 10 | CGR5873 | ICR00093 | 30 | BNL243 | CER0168 | 5.59 | 2.62 | 0.48 | -0.87 | 0.32 | -0.22 | 0.40 | -0.51 |
|  | 16 | SWU10266 | DC40065 | 30 | CER0168 | SWU21718 | 6.00 | 2.37 | 1.00 | 0.82 | 0.21 | -0.36 | -0.60 | 0.75 |
| BNP | 1 | ICR03724 | ICR03725 | 2 | PGML0700 | SWU12016 | 6.12 | 2.31 | 0.75 | -0.40 | 0.24 | 0.13 | 0.01 | -0.38 |
|  | 4 | ICR01729 | SWU16781 | 9 | NAU1282 | CGR6771 | 5.27 | 1.36 | 0.82 | 0.31 | 0.10 | -0.02 | -0.37 | 0.29 |
|  | 7 | CGR5372 | SWU10205 | 9 | NAU3966 | SWU15157 | 5.39 | 2.42 | 0.48 | 0.41 | -0.16 | -0.13 | -0.02 | 0.31 |
|  | 6 | NAU896 | BNL3650 | 10 | BNL2960 | SWU20511 | 6.00 | 2.21 | 0.67 | -0.40 | 0.10 | -0.30 | 0.29 | -0.09 |
|  | 5 | SWU17787 | SWU13378 | 11 | SWU15972 | TMB0628 | 5.14 | 0.71 | 2.13 | 0.22 | 0.21 | -0.50 | -0.22 | 0.51 |
|  | 7 | **SWU10067** | SWU10064 | 13 | SWU22413 | CGR5331 | 5.59 | 1.88 | 1.28 | 0.37 | 0.34 | -0.42 | -0.16 | 0.23 |
|  | 13 | DPL0894 | SWU10800 | 14 | BNL3661 | PGML2498 | 5.09 | 1.83 | 0.87 | 0.36 | -0.26 | 0.08 | -0.21 | 0.39 |
|  | 5 | SWU20913 | Gh260 | 16 | **SWU10266** | DC40065 | 5.92 | 2.61 | 0.40 | -0.43 | -0.16 | -0.04 | 0.28 | -0.08 |
|  | 2 | SWU12126 | SWU12147 | 16 | NAU2984 | SWU10062 | 5.14 | 1.75 | 0.74 | 0.35 | -0.09 | 0.16 | -0.34 | 0.28 |
|  | 1 | CGR6129 | DPL0790 | 19 | NAU1042 | **NAU3437** | 5.73 | 2.93 | 0.07 | 0.45 | -0.03 | 0.11 | -0.09 | 0.00 |
|  | 18 | SWU22287 | SWU22290 | 20 | CER0167 | SWU20064 | 5.39 | 2.69 | 0.02 | 0.43 | 0.00 | -0.03 | -0.03 | 0.06 |
|  | 2 | PGML0700 | SWU12016 | 21 | Gh451 | SWU16489 | 5.73 | 2.76 | 0.25 | -0.44 | 0.20 | -0.05 | 0.01 | -0.16 |
|  | 16 | **SWU10214** | **Gh56** | 21 | CGR5808 | HAU0423 | 5.42 | 2.28 | 0.56 | 0.40 | 0.18 | 0.24 | -0.24 | -0.18 |
|  | 19 | DC40122 | NAU833a | 22 | PGML1712 | SWU21538 | 5.03 | 2.47 | 0.42 | 0.43 | -0.13 | 0.26 | -0.19 | 0.06 |
|  | 5 | PGML1917 | SWU17715 | 24 | BNL1521 | HAU2504 | 5.17 | 2.26 | 0.42 | 0.40 | 0.17 | 0.12 | -0.28 | -0.01 |
|  | 19 | **NAU3437** | **NAU2894** | 24 | BNL1521 | HAU2504 | 7.13 | 2.29 | 1.29 | -0.40 | -0.22 | 0.27 | 0.32 | -0.38 |
|  | 11 | NAU2152 | NAU5428 | 25 | HAU1382 | SWU19848 | 5.36 | 1.79 | 1.30 | -0.35 | 0.39 | -0.06 | 0.11 | -0.44 |
|  | 5 | CGR5025 | NBRI0694 | 26 | DPL0491 | Gh64 | 6.00 | 3.05 | 0.41 | 0.46 | -0.03 | -0.12 | -0.14 | 0.29 |
|  | 13 | SWU22413 | CGR5331 | 26 | C2_0135 | PGML2321 | 5.67 | 2.68 | 0.07 | -0.44 | -0.06 | -0.11 | 0.10 | 0.06 |
|  | 9 | PGML2830 | CGR6876 | 26 | SWU18681 | SWU0598 | 8.17 | 2.83 | 0.75 | -0.44 | -0.29 | -0.02 | 0.35 | -0.04 |
|  | 1 | SWU0077 | HAU1417 | 27 | SWU10994 | HAU1001 | 5.87 | 2.66 | 0.56 | 0.44 | -0.10 | 0.01 | -0.23 | 0.32 |
|  | 16 | SWU10094 | SWU10060 | 27 | SWU11384 | ICR11885 | 5.20 | 2.36 | 0.17 | 0.41 | 0.14 | -0.09 | -0.07 | 0.02 |
|  | 20 | SWU20675 | SWU20649 | 27 | **CGR6857** | **ICR11205** | 5.78 | 2.46 | 0.51 | -0.42 | -0.13 | -0.10 | 0.36 | -0.13 |
|  | 11 | SWU15972 | TMB0628 | 28 | BNL2877 | HAU3071 | 5.00 | 1.89 | 0.78 | 0.36 | 0.34 | -0.20 | -0.23 | 0.09 |
|  | 13 | DPL0894 | SWU10800 | 31 | SWU16676 | SWU16755 | 6.40 | 3.35 | 0.12 | -0.49 | 0.10 | 0.08 | -0.10 | -0.08 |
| BW | 1 | DPL0090 | Gh398 | 1 | HAU1417 | NAU2437 | 5.22 | 2.30 | 0.05 | -0.07 | -0.01 | 0.00 | 0.00 | 0.02 |
|  | 1 | NAU6367 | MUSS422 | 2 | SWU12025 | SWU11889 | 7.06 | 3.33 | 0.20 | 0.08 | -0.03 | 0.03 | 0.01 | 0.00 |
|  | 1 | NAU2218 | SWU11191 | 4 | ICR01729 | SWU16781 | 5.10 | 2.43 | 0.49 | -0.07 | -0.03 | -0.02 | 0.05 | 0.00 |
|  | 5 | PGML1917 | SWU17715 | 8 | CGR6508 | Gh197 | 5.09 | 2.28 | 0.00 | 0.07 | 0.00 | 0.00 | 0.00 | 0.01 |
|  | 5 | **NAU6240** | PGML1671 | 9 | Gh158 | DC40407 | 6.25 | 2.75 | 0.19 | -0.08 | 0.02 | -0.02 | -0.02 | 0.02 |
|  | 1 | NAU3177 | ICR03724 | 10 | SWU20501b | CGR5873 | 5.56 | 2.55 | 0.00 | -0.07 | 0.01 | 0.00 | -0.01 | 0.01 |
|  | 8 | CGR6508 | Gh197 | 12 | DPL0732 | Gh631 | 6.18 | 2.70 | 0.14 | -0.07 | 0.00 | 0.03 | -0.02 | 0.00 |
|  | 6 | ICR03206 | NAU896 | 13 | SWU22374 | HAU2857 | 8.12 | 3.80 | 0.14 | -0.09 | 0.02 | 0.00 | 0.00 | -0.02 |
|  | 13 | SWU22413 | CGR5331 | 13 | SWU22374 | HAU2857 | 6.61 | 1.90 | 0.06 | 0.10 | -0.01 | 0.05 | -0.03 | -0.01 |
|  | 4 | SWU21617 | SWU11855 | 13 | DPL0894 | SWU10800 | 5.22 | 2.39 | 0.07 | 0.07 | -0.02 | 0.02 | 0.00 | 0.00 |
|  | 5 | DPL0022 | SWU17787 | 14 | PGML4763 | SWU13909 | 5.53 | 2.65 | 0.03 | 0.07 | 0.00 | -0.01 | 0.00 | 0.01 |
|  | 7 | NAU1357 | SWU10067 | 14 | PGML4763 | SWU13909 | 5.93 | 2.82 | 0.20 | 0.08 | -0.03 | 0.01 | 0.00 | 0.02 |
|  | 14 | TMB0071 | BNL3661 | 14 | SWU14224 | DPL0565 | 5.23 | 2.33 | 0.04 | 0.07 | 0.00 | -0.01 | 0.01 | 0.00 |
|  | 3 | HAU2424 | CER0028 | 14 | SWU14224 | DPL0565 | 5.51 | 2.48 | 0.08 | 0.07 | 0.00 | 0.01 | 0.01 | -0.02 |
|  | 12 | NAU943 | DPL0303 | 14 | PGML1568 | Gh529 | 5.06 | 2.40 | 0.23 | 0.07 | 0.01 | -0.01 | 0.03 | -0.03 |
|  | 5 | SWU17787 | SWU13378 | 15 | CGR6889 | DPL0182 | 6.51 | 3.04 | 0.12 | 0.08 | -0.02 | 0.01 | 0.02 | 0.00 |
|  | 11 | NAU5428 | Gh256 | 16 | PGML1709 | SWU10627 | 6.57 | 3.08 | 0.07 | -0.08 | 0.01 | -0.01 | -0.01 | 0.02 |
|  | 14 | PGML1568 | Gh529 | 16 | NAU862 | CGR6802 | 6.03 | 2.65 | 0.07 | 0.07 | -0.02 | 0.01 | 0.02 | -0.01 |
|  | 2 | PGML0700 | SWU12016 | 16 | CGR6802 | HAU1129 | 5.53 | 2.52 | 0.05 | -0.07 | -0.01 | -0.01 | 0.00 | 0.02 |
|  | 7 | CGR5372 | SWU10205 | 16 | **SWU10038** | **ICR00016** | 5.78 | 2.68 | 0.23 | 0.07 | 0.03 | 0.00 | -0.03 | 0.00 |
|  | 13 | BNL1495 | CGR5390 | 17 | SWU12818 | CGR5576 | 7.05 | 3.17 | 0.14 | 0.08 | 0.03 | 0.00 | -0.02 | -0.01 |
|  | 10 | ICR00093 | ICR07050 | 18 | NAU748 | SWU22192 | 6.25 | 2.88 | 0.05 | -0.08 | 0.02 | -0.01 | 0.00 | 0.00 |
|  | 13 | NAU3468 | SWU22309 | 18 | SWU22187 | DC40150 | 5.42 | 1.79 | 0.08 | 0.07 | 0.02 | 0.01 | -0.02 | -0.01 |
|  | 1 | NAU3384 | CGR5663 | 19 | NAU1042 | NAU3437 | 5.67 | 2.65 | 0.14 | 0.07 | 0.03 | 0.00 | -0.02 | 0.00 |
|  | 9 | NAU3966 | SWU15157 | 19 | NAU3437 | NAU2894 | 5.93 | 2.78 | 0.13 | -0.08 | 0.03 | 0.00 | -0.02 | -0.01 |
|  | 16 | **SWU10038** | **ICR00016** | 19 | NAU3437 | NAU2894 | 6.04 | 2.65 | 0.04 | 0.07 | 0.01 | 0.00 | -0.01 | -0.01 |
|  | 8 | Gh197 | DC20094 | 19 | SWU17882 | CAU0104 | 7.25 | 3.32 | 0.11 | 0.08 | 0.01 | 0.01 | 0.00 | -0.02 |
|  | 9 | PGML2830 | CGR6876 | 20 | CGR5548 | SWU20675 | 5.17 | 2.29 | 0.38 | -0.07 | 0.02 | 0.01 | -0.05 | 0.02 |
|  | 19 | NAU3437 | NAU2894 | 20 | SWU20675 | SWU20649 | 5.99 | 2.79 | 0.06 | -0.07 | 0.00 | -0.01 | 0.02 | 0.00 |
|  | 16 | Gh56 | NAU5120 | 20 | **SWU20501a** | **CER0167** | 6.28 | 3.07 | 0.16 | 0.08 | 0.00 | 0.03 | -0.01 | -0.02 |
|  | 5 | DPL0022 | SWU17787 | 21 | Gh451 | SWU16489 | 6.74 | 3.18 | 0.05 | -0.08 | -0.01 | 0.00 | 0.02 | -0.01 |
|  | 7 | SWU10785 | CER0036 | 21 | SWU16487 | SWU16488 | 6.00 | 2.63 | 0.08 | 0.07 | 0.00 | 0.01 | 0.01 | -0.02 |
|  | 8 | HAU1470a | SHIN1341 | 21 | SWU0830 | HAU2004 | 5.65 | 2.66 | 0.30 | 0.07 | 0.04 | -0.02 | -0.03 | 0.01 |
|  | 13 | SWU22413 | CGR5331 | 21 | CGR5806 | DPL0777 | 5.54 | 2.60 | 0.01 | 0.07 | 0.00 | 0.00 | -0.01 | 0.00 |
|  | 16 | CGR6802 | HAU1129 | 21 | CGR5806 | DPL0777 | 5.89 | 2.69 | 0.04 | -0.08 | 0.01 | 0.00 | 0.01 | -0.02 |
|  | 14 | SWU14224 | DPL0565 | 22 | CAU0161 | NAU2026 | 7.32 | 3.53 | 0.23 | 0.09 | -0.03 | 0.02 | 0.03 | -0.02 |
|  | 19 | NAU5330 | Gh72 | 22 | PGML0695 | SWU20813 | 6.94 | 3.32 | 0.23 | 0.08 | 0.03 | 0.01 | -0.03 | -0.01 |
|  | 7 | SWU10205 | HAU1483a | 23 | SWU14807 | PGML4185 | 6.00 | 2.81 | 0.09 | -0.08 | 0.02 | -0.02 | 0.00 | 0.00 |
|  | 18 | NAU748 | SWU22192 | 23 | SWU14807 | PGML4185 | 5.08 | 2.37 | 0.08 | -0.07 | 0.00 | -0.02 | 0.00 | 0.02 |
|  | 21 | SWU16487 | SWU16488 | 24 | CGR5202 | Gh298 | 5.16 | 2.32 | 0.60 | 0.07 | -0.01 | -0.01 | 0.06 | -0.04 |
|  | 9 | Gh158 | DC40407 | 24 | Gh298 | SWU13133 | 5.56 | 2.50 | 0.16 | 0.07 | -0.03 | 0.01 | 0.02 | 0.01 |
|  | 24 | SWU13267 | BNL1521 | 24 | BNL1521 | **HAU2504** | 6.23 | 2.85 | 0.06 | -0.08 | -0.01 | -0.01 | -0.02 | 0.03 |
|  | 20 | SWU20246 | **SWU20501a** | 24 | BNL1521 | **HAU2504** | 5.99 | 2.90 | 0.28 | -0.07 | -0.02 | -0.02 | 0.04 | 0.00 |
|  | 5 | **NAU6240** | PGML1671 | 25 | HAU1382 | SWU19848 | 6.61 | 2.88 | 0.05 | 0.08 | 0.00 | 0.01 | -0.01 | 0.00 |
|  | 6 | CGR5108 | ICR03206 | 25 | SWU19848 | CGR6864 | 7.53 | 3.52 | 0.11 | 0.08 | 0.00 | 0.02 | 0.01 | -0.02 |
|  | 20 | SWU20675 | SWU20649 | 25 | BNL3594 | DPL0282 | 5.30 | 2.41 | 0.10 | 0.07 | 0.00 | 0.01 | -0.02 | 0.01 |
|  | 8 | CGR6508 | Gh197 | 25 | SWU19763 | SWU19129 | 6.71 | 2.93 | 0.17 | 0.08 | -0.01 | 0.03 | 0.00 | -0.02 |
|  | 21 | SWU0830 | HAU2004 | 25 | SWU19129 | PGML2858 | 6.11 | 2.85 | 0.09 | -0.08 | -0.03 | 0.02 | 0.01 | 0.00 |
|  | 12 | NAU943 | DPL0303 | 25 | SWU19144 | Gh220 | 5.17 | 2.35 | 0.08 | 0.07 | 0.01 | -0.02 | 0.02 | -0.01 |
|  | 4 | SWU18876 | SWU12672 | 25 | SWU19430 | PGML1219 | 6.56 | 3.01 | 0.10 | -0.08 | -0.02 | -0.01 | 0.02 | 0.01 |
|  | 9 | CGR6876 | CGR5758 | 25 | SWU19430 | PGML1219 | 5.44 | 2.35 | 0.06 | -0.07 | 0.00 | -0.01 | 0.02 | -0.01 |
|  | 19 | **SWU17897** | **CGR5539** | 26 | MGHES31 | HAU1571 | 7.02 | 3.17 | 0.23 | 0.08 | 0.00 | 0.03 | -0.03 | 0.00 |
|  | 6 | ICR10602 | SWU19656 | 26 | SWU17467 | SWU17419 | 5.82 | 2.66 | 0.08 | -0.07 | 0.00 | -0.01 | 0.02 | -0.01 |
|  | 18 | CIR099 | NAU748 | 26 | SWU17432 | SWU17395 | 5.56 | 2.51 | 0.09 | 0.07 | 0.01 | -0.02 | 0.00 | 0.02 |
|  | 24 | SWU13758 | CGR5423 | 26 | SWU17432 | SWU17395 | 9.42 | 4.13 | 0.02 | 0.09 | 0.01 | 0.00 | 0.00 | -0.01 |
|  | 17 | NAU3765 | SWU14627 | 26 | DPL0491 | Gh64 | 5.29 | 2.30 | 0.12 | -0.07 | -0.01 | -0.01 | -0.01 | 0.03 |
|  | 21 | SWU16651 | SWU16645 | 26 | DPL0491 | Gh64 | 7.46 | 3.52 | 0.30 | -0.08 | 0.00 | -0.01 | 0.04 | -0.03 |
|  | 13 | SWU13032 | DPL0308 | 26 | SWU17251 | C2_0135 | 7.02 | 3.27 | 0.05 | -0.08 | 0.01 | -0.01 | 0.01 | -0.01 |
|  | 1 | HAU1417 | NAU2437 | 26 | C2_0135 | PGML2321 | 5.73 | 2.66 | 0.04 | 0.07 | -0.02 | 0.00 | 0.01 | 0.01 |
|  | 14 | **NAU874** | SWU13824 | 26 | SWU18488 | **SWU18672** | 5.96 | 2.73 | 0.05 | -0.07 | 0.01 | 0.01 | 0.00 | -0.02 |
|  | 26 | C2_0135 | PGML2321 | 26 | SWU18488 | **SWU18672** | 5.37 | 2.00 | 0.02 | 0.08 | -0.01 | 0.02 | -0.01 | 0.00 |
|  | 21 | CGR5217 | BNL3442a | 27 | SWU11038 | SWU11384 | 6.73 | 2.96 | 0.03 | -0.08 | -0.01 | 0.00 | 0.01 | 0.00 |
|  | 26 | SWU17251 | C2_0135 | 27 | SWU11384 | ICR11885 | 6.08 | 2.82 | 0.06 | 0.08 | 0.01 | 0.00 | 0.01 | -0.02 |
|  | 17 | CGR5576 | NAU3765 | 28 | BNL2877 | HAU3071 | 7.47 | 3.52 | 0.25 | 0.08 | 0.02 | 0.02 | -0.02 | -0.02 |
|  | 9 | NAU1282 | CGR6771 | 28 | HAU3071 | CGR5534 | 5.48 | 2.45 | 0.05 | 0.07 | 0.01 | -0.02 | 0.01 | 0.00 |
|  | 10 | ICR00093 | ICR07050 | 28 | HAU3071 | CGR5534 | 5.23 | 2.47 | 0.08 | 0.07 | 0.02 | 0.00 | -0.01 | -0.01 |
|  | 14 | PGML1368 | PGML1568 | 28 | CGR5534 | SHIN0219 | 6.04 | 2.88 | 0.05 | 0.08 | -0.02 | 0.01 | 0.01 | 0.00 |
|  | 5 | SWU17787 | SWU13378 | 28 | SHIN0219 | TMB2386 | 5.92 | 2.80 | 0.24 | 0.07 | -0.03 | 0.02 | 0.00 | 0.02 |
|  | 20 | SWU20636 | CGR6154 | 28 | SHIN0219 | TMB2386 | 5.27 | 2.35 | 0.12 | 0.07 | -0.02 | 0.02 | 0.00 | 0.01 |
|  | 7 | NAU1357 | SWU10067 | 28 | NBRI0014 | SWU12107 | 5.20 | 2.55 | 0.27 | 0.07 | 0.02 | 0.02 | -0.01 | -0.03 |
|  | 24 | SWU13267 | BNL1521 | 29 | DC20127 | DPL0252 | 5.63 | 2.60 | 0.23 | -0.07 | 0.03 | -0.03 | -0.01 | 0.01 |
|  | 19 | DC40122 | NAU833a | 29 | DC20127 | DPL0252 | 6.46 | 2.89 | 0.17 | 0.08 | -0.01 | 0.03 | -0.02 | 0.01 |
|  | 15 | DC40183 | DC40175 | 29 | **C2_0115** | **ICR03107** | 5.39 | 2.44 | 0.01 | -0.07 | 0.00 | 0.00 | 0.01 | -0.01 |
|  | 14 | TMB0071 | BNL3661 | 29 | **C2_0115** | **ICR03107** | 5.46 | 2.53 | 0.22 | -0.07 | 0.00 | -0.02 | 0.03 | -0.01 |
|  | 25 | SWU19411 | SWU19412 | 29 | **C2_0115** | **ICR03107** | 5.39 | 2.61 | 0.09 | 0.07 | 0.02 | 0.00 | -0.02 | 0.00 |
|  | 13 | BNL1495 | CGR5390 | 30 | **BNL243** | **CER0168** | 5.95 | 2.59 | 0.09 | 0.08 | 0.03 | 0.00 | -0.01 | -0.02 |
|  | 28 | NBRI0014 | SWU12107 | 32 | NAU2140 | NAU2957 | 6.25 | 3.10 | 0.25 | 0.08 | 0.01 | -0.04 | 0.02 | 0.01 |
| LP | 1 | SWU10912 | DPL0090 | 1 | CGR5663 | NAU2343 | 5.37 | 2.55 | 0.11 | 0.35 | -0.09 | -0.03 | 0.03 | 0.09 |
|  | 4 | ICR01729 | SWU16781 | 6 | CGR5801 | SWU19249 | 5.75 | 2.52 | 0.12 | -0.35 | 0.08 | 0.03 | 0.00 | -0.12 |
|  | 2 | SWU11887 | SWU11976 | 7 | SWU10067 | **SWU10064** | 6.05 | 2.70 | 0.12 | -0.35 | -0.05 | 0.12 | -0.04 | -0.03 |
|  | 1 | SWU10912 | DPL0090 | 7 | **SWU10064** | **NAU3181** | 6.98 | 3.07 | 0.08 | 0.38 | 0.01 | -0.09 | 0.09 | -0.01 |
|  | 4 | SWU12672 | HAU1332 | 7 | SWU10205 | HAU1483a | 5.92 | 2.63 | 0.08 | -0.36 | -0.02 | -0.02 | 0.10 | -0.06 |
|  | 5 | **TMB1296** | **HAU1603** | 7 | CER0036 | PGML1916 | 6.66 | 2.99 | 0.05 | -0.38 | 0.05 | 0.03 | -0.02 | -0.06 |
|  | 7 | PGML1916 | SWU10864 | 9 | NAU5474 | Gh158 | 6.90 | 3.06 | 0.12 | -0.42 | 0.10 | 0.03 | -0.10 | -0.02 |
|  | 5 | SWU20913 | Gh260 | 9 | SWU15157 | SWU14934 | 8.42 | 3.32 | 0.38 | 0.40 | 0.02 | -0.19 | 0.19 | -0.03 |
|  | 1 | ICR03724 | ICR03725 | 10 | ICR00093 | ICR07050 | 6.89 | 3.08 | 0.09 | 0.39 | 0.03 | -0.09 | 0.06 | 0.00 |
|  | 10 | ICR00093 | ICR07050 | 10 | ICR00093 | ICR07050 | 5.04 | 2.02 | 0.05 | -0.41 | -0.02 | 0.03 | 0.05 | -0.06 |
|  | 2 | **SWU11889** | SWU11887 | 11 | NAU2152 | NAU5428 | 5.51 | 2.37 | 0.20 | -0.34 | 0.15 | -0.04 | 0.01 | -0.12 |
|  | 3 | **SWU12819** | **SWU12765** | 11 | CER0098 | CGR5421 | 7.85 | 3.27 | 0.26 | -0.40 | 0.14 | 0.06 | -0.04 | -0.16 |
|  | 5 | SWU13378 | SWU17846 | 11 | **ICR01810** | CGR6525 | 5.88 | 2.76 | 0.05 | 0.36 | -0.01 | -0.07 | 0.01 | 0.07 |
|  | 9 | Gh27 | SWU15194 | 11 | PGML2202 | CGR6580 | 5.18 | 2.40 | 0.07 | 0.34 | -0.02 | -0.09 | 0.03 | 0.08 |
|  | 4 | SWU18876 | SWU12672 | 11 | SWU15972 | TMB0628 | 8.06 | 3.42 | 0.23 | 0.40 | -0.15 | -0.03 | 0.04 | 0.14 |
|  | 11 | PGML2202 | CGR6580 | 12 | HAU1316 | NAU3519 | 6.26 | 2.67 | 0.17 | 0.36 | -0.01 | -0.07 | -0.08 | 0.16 |
|  | 3 | **SWU12819** | **SWU12765** | 13 | SWU22309 | SWU22324 | 5.50 | 2.27 | 0.13 | 0.34 | -0.01 | -0.13 | 0.07 | 0.06 |
|  | 13 | SWU22413 | CGR5331 | 13 | **NAU2893** | **Gh157** | 5.05 | 1.87 | 0.44 | -0.33 | -0.05 | 0.14 | -0.23 | 0.14 |
|  | 1 | ICR03724 | ICR03725 | 13 | DPL0894 | SWU10800 | 7.09 | 3.35 | 0.05 | 0.40 | -0.04 | -0.03 | 0.08 | -0.01 |
|  | 5 | PGML4350 | SWU17781 | 14 | PGML4763 | SWU13909 | 8.15 | 3.65 | 0.20 | 0.42 | -0.06 | -0.12 | 0.14 | 0.05 |
|  | 6 | ICR00143 | CGR5108 | 14 | PGML4763 | SWU13909 | 5.31 | 1.86 | 0.41 | 0.30 | -0.21 | 0.02 | -0.01 | 0.20 |
|  | 13 | HAU2558 | **NAU2893** | 14 | PGML4763 | SWU13909 | 6.50 | 3.16 | 0.14 | 0.39 | -0.13 | -0.02 | 0.11 | 0.05 |
|  | 7 | CGR5001 | CGR6586 | 14 | PGML1568 | Gh529 | 5.96 | 2.67 | 0.06 | -0.36 | -0.01 | 0.08 | -0.06 | -0.01 |
|  | 3 | CER0028 | Gh663 | 15 | DPL0182 | SWU11691 | 7.06 | 2.98 | 0.31 | 0.38 | 0.04 | -0.16 | -0.04 | 0.16 |
|  | 5 | **SWU20917** | **NAU6240** | 16 | SWU10627 | PGML1309 | 9.00 | 3.67 | 0.58 | 0.42 | -0.15 | 0.00 | 0.27 | -0.13 |
|  | 2 | SWU12126 | SWU12147 | 16 | SWU10214 | Gh56 | 5.08 | 2.04 | 0.43 | 0.31 | -0.09 | -0.04 | 0.24 | -0.12 |
|  | 4 | SWU16783 | NAU3868 | 16 | CGR6802 | HAU1129 | 5.15 | 2.21 | 0.11 | -0.33 | -0.11 | 0.05 | -0.01 | 0.07 |
|  | 13 | DPL0894 | SWU10800 | 16 | NAU2984 | SWU10062 | 5.77 | 1.69 | 0.64 | 0.28 | -0.02 | -0.25 | 0.25 | 0.02 |
|  | 10 | BNL2960 | SWU20511 | 16 | SWU10062 | SWU10094 | 5.33 | 2.12 | 0.17 | -0.32 | -0.10 | 0.14 | 0.05 | -0.08 |
|  | 14 | ICR12037 | CGR5675 | 16 | SWU10056 | SWU10037 | 7.00 | 3.17 | 0.10 | 0.39 | 0.11 | -0.06 | -0.04 | -0.01 |
|  | 11 | CER0098 | CGR5421 | 17 | SWU14627 | CGR5871 | 6.13 | 2.75 | 0.10 | -0.37 | -0.03 | 0.12 | 0.00 | -0.09 |
|  | 13 | DPL0894 | SWU10800 | 18 | Gh60 | SWU22281 | 5.36 | 2.14 | 0.27 | -0.32 | 0.07 | 0.13 | -0.16 | -0.04 |
|  | 17 | SWU12818 | CGR5576 | 18 | CIR099 | NAU748 | 5.39 | 2.33 | 0.19 | -0.33 | 0.03 | 0.07 | 0.05 | -0.16 |
|  | 7 | SWU10067 | **SWU10064** | 19 | NAU5330 | Gh72 | 5.47 | 2.33 | 0.36 | 0.34 | 0.04 | 0.00 | -0.20 | 0.16 |
|  | 18 | CIR099 | NAU748 | 19 | SWU17782 | DPL0056 | 6.33 | 2.60 | 0.27 | -0.35 | 0.08 | 0.03 | -0.19 | 0.09 |
|  | 8 | HAU1470a | SHIN1341 | 19 | TMB0107 | NAU3217 | 6.82 | 2.66 | 0.38 | -0.36 | 0.20 | -0.08 | -0.16 | 0.04 |
|  | 7 | CGR5001 | CGR6586 | 20 | SWU20700 | CGR5548 | 5.57 | 2.26 | 0.36 | -0.34 | -0.08 | 0.06 | 0.18 | -0.17 |
|  | 3 | SWU12840 | NAU2742 | 20 | SWU20636 | CGR6154 | 6.70 | 3.00 | 0.21 | 0.38 | -0.14 | 0.09 | 0.09 | -0.04 |
|  | 11 | NAU3390 | NAU2460 | 20 | SWU20501a | **CER0167** | 5.65 | 2.58 | 0.03 | 0.35 | 0.00 | 0.05 | 0.02 | -0.07 |
|  | 6 | NAU896 | **BNL3650** | 20 | DPL0319 | HAU1378 | 8.78 | 3.54 | 0.27 | -0.42 | -0.14 | 0.13 | 0.10 | -0.08 |
|  | 18 | CIR099 | NAU748 | 21 | Gh451 | SWU16489 | 6.69 | 2.84 | 0.18 | -0.37 | 0.06 | 0.12 | -0.13 | -0.05 |
|  | 4 | ICR01729 | SWU16781 | 21 | Gh451 | SWU16489 | 8.28 | 3.26 | 0.46 | 0.40 | -0.15 | -0.14 | 0.09 | 0.20 |
|  | 20 | SWU20035 | DPL0319 | 21 | Gh451 | SWU16489 | 6.04 | 2.83 | 0.01 | 0.37 | -0.01 | -0.04 | -0.01 | 0.06 |
|  | 11 | CER0098 | CGR5421 | 21 | SWU16488 | SWU16138 | 6.33 | 2.70 | 0.25 | 0.36 | 0.00 | 0.06 | -0.18 | 0.12 |
|  | 21 | Gh451 | SWU16489 | 21 | SWU16488 | SWU16138 | 7.31 | 3.18 | 0.00 | -0.43 | 0.02 | -0.02 | 0.03 | -0.03 |
|  | 16 | DPL0048 | SWU10266 | 21 | SWU14431a | SWU15915 | 7.19 | 3.26 | 0.21 | -0.40 | 0.08 | -0.05 | -0.14 | 0.11 |
|  | 2 | SWU11887 | SWU11976 | 21 | BNL3171 | CGR5808 | 5.42 | 2.53 | 0.01 | -0.35 | 0.03 | -0.01 | 0.01 | -0.02 |
|  | 13 | HAU2558 | **NAU2893** | 21 | BNL3171 | CGR5808 | 7.87 | 3.65 | 0.12 | -0.41 | 0.12 | -0.03 | -0.11 | 0.01 |
|  | 1 | ICR03724 | ICR03725 | 21 | HAU0423 | CGR5806 | 6.12 | 2.45 | 0.36 | 0.34 | -0.12 | -0.12 | 0.03 | 0.20 |
|  | 20 | CGR6154 | SWU20246 | 22 | **SWU21635** | **SWU21646** | 5.92 | 2.69 | 0.15 | 0.35 | 0.12 | -0.09 | -0.08 | 0.04 |
|  | 5 | PGML1917 | SWU17715 | 22 | SWU21586 | **PGML1712** | 6.06 | 2.78 | 0.05 | -0.37 | 0.06 | 0.02 | -0.07 | -0.01 |
|  | 10 | ICR00093 | ICR07050 | 22 | SWU21586 | **PGML1712** | 5.11 | 1.91 | 0.46 | -0.31 | 0.13 | 0.16 | -0.10 | -0.19 |
|  | 18 | SWU22287 | SWU22290 | 22 | **CAU0161** | NAU2026 | 6.52 | 2.96 | 0.18 | 0.40 | 0.09 | -0.13 | -0.06 | 0.10 |
|  | 19 | NAU5330 | Gh72 | 22 | **CAU0161** | NAU2026 | 5.05 | 2.34 | 0.04 | -0.37 | 0.08 | 0.01 | -0.02 | -0.07 |
|  | 19 | **NAU3437** | **NAU2894** | 23 | SWU14807 | PGML4185 | 5.67 | 2.43 | 0.17 | 0.34 | 0.04 | -0.14 | 0.11 | -0.02 |
|  | 1 | NAU3384 | CGR5663 | 24 | SWU13150 | PGML4657 | 6.25 | 2.56 | 0.37 | 0.35 | 0.07 | -0.24 | 0.07 | 0.09 |
|  | 10 | HAU0635 | NAU2139 | 24 | PGML4657 | Gh454 | 7.86 | 2.66 | 0.75 | -0.36 | 0.25 | 0.11 | -0.20 | -0.16 |
|  | 9 | Gh111 | Gh27 | 24 | SWU13267 | BNL1521 | 7.47 | 3.22 | 0.33 | 0.39 | -0.01 | -0.20 | 0.11 | 0.10 |
|  | 20 | SWU20246 | SWU20501a | 24 | SWU13267 | BNL1521 | 5.83 | 2.35 | 0.37 | 0.33 | 0.01 | -0.22 | 0.13 | 0.09 |
|  | 21 | SWU0830 | HAU2004 | 24 | SWU13267 | BNL1521 | 5.63 | 2.32 | 0.29 | 0.34 | -0.14 | -0.05 | -0.01 | 0.20 |
|  | 24 | Gh298 | SWU13133 | 24 | BNL1521 | HAU2504 | 6.27 | 2.67 | 0.17 | 0.36 | 0.05 | -0.14 | 0.10 | 0.00 |
|  | 13 | SHIN1462 | SWU22374 | 24 | SWU13745 | Gh273 | 5.49 | 2.49 | 0.20 | 0.35 | 0.11 | -0.04 | 0.07 | -0.14 |
|  | 5 | **SWU20917** | **NAU6240** | 25 | **SWU19848** | **CGR6864** | 6.77 | 2.84 | 0.13 | 0.36 | -0.01 | -0.07 | -0.04 | 0.12 |
|  | 22 | **SWU21646** | SWU21585 | 25 | BNL3594 | DPL0282 | 5.41 | 2.31 | 0.08 | -0.33 | 0.03 | 0.06 | 0.02 | -0.11 |
|  | 12 | HAU1316 | NAU3519 | 25 | DPL0282 | SWU19763 | 7.42 | 2.99 | 0.35 | 0.38 | -0.03 | -0.20 | 0.11 | 0.12 |
|  | 13 | DPL0535 | CER0165 | 25 | SWU19430 | PGML1219 | 5.37 | 2.30 | 0.11 | -0.33 | -0.01 | 0.12 | -0.08 | -0.03 |
|  | 21 | SWU16138 | BNL1053 | 26 | MGHES31 | HAU1571 | 7.66 | 3.50 | 0.00 | -0.42 | 0.03 | 0.02 | -0.05 | 0.00 |
|  | 7 | Gh474 | SWU10785 | 26 | SWU17467 | SWU17419 | 6.14 | 2.51 | 0.31 | -0.35 | 0.14 | 0.10 | -0.15 | -0.09 |
|  | 9 | SWU15157 | SWU14934 | 26 | BNL2495 | DPL0491 | 6.59 | 2.91 | 0.24 | 0.37 | 0.13 | 0.01 | 0.03 | -0.17 |
|  | 18 | Gh60 | SWU22281 | 26 | Gh64 | SWU17257 | 5.20 | 2.21 | 0.28 | -0.32 | 0.09 | 0.08 | -0.19 | 0.03 |
|  | 22 | **DPL0562** | **CAU0161** | 26 | Gh64 | SWU17257 | 5.60 | 2.08 | 0.26 | 0.33 | 0.17 | -0.13 | 0.03 | -0.07 |
|  | 13 | SWU22413 | CGR5331 | 26 | SWU0514 | SWU18488 | 6.08 | 2.86 | 0.06 | -0.37 | -0.06 | 0.04 | 0.02 | -0.01 |
|  | 8 | CGR6508 | Gh197 | 26 | SWU18488 | SWU18672 | 5.26 | 2.44 | 0.06 | -0.34 | 0.07 | -0.10 | 0.01 | 0.02 |
|  | 14 | SWU14224 | DPL0565 | 26 | SWU18488 | SWU18672 | 5.66 | 2.59 | 0.05 | -0.35 | 0.01 | 0.06 | 0.01 | -0.08 |
|  | 26 | **C2_0135** | PGML2321 | 26 | SWU18488 | SWU18672 | 6.72 | 2.43 | 0.13 | -0.40 | 0.03 | 0.08 | -0.09 | -0.02 |
|  | 3 | SWU12840 | NAU2742 | 26 | SWU18681 | SWU0598 | 5.47 | 2.47 | 0.05 | -0.34 | -0.01 | -0.07 | 0.02 | 0.06 |
|  | 11 | CGR5421 | ICR08245 | 26 | SWU18681 | SWU0598 | 5.28 | 2.06 | 0.33 | -0.31 | -0.18 | 0.17 | 0.04 | -0.02 |
|  | 16 | SWU10038 | ICR00016 | 27 | SWU10994 | HAU1001 | 5.45 | 2.06 | 0.10 | 0.32 | 0.02 | -0.12 | 0.05 | 0.05 |
|  | 3 | HAU2424 | CER0028 | 27 | **SWU11038** | **SWU11384** | 5.84 | 2.57 | 0.10 | -0.36 | -0.04 | 0.11 | 0.00 | -0.07 |
|  | 25 | BNL3594 | DPL0282 | 27 | SWU11384 | ICR11885 | 5.55 | 2.52 | 0.04 | 0.35 | -0.01 | -0.06 | 0.00 | 0.07 |
|  | 6 | CGR5108 | ICR03206 | 27 | ICR11205 | DPL0847a | 8.42 | 3.17 | 0.50 | -0.39 | -0.13 | 0.24 | 0.04 | -0.15 |
|  | 14 | **NAU2960** | ICR12130 | 28 | CGR5534 | SHIN0219 | 5.33 | 2.13 | 0.24 | -0.32 | -0.02 | -0.03 | 0.17 | -0.12 |
|  | 19 | SWU17897 | CGR5539 | 28 | SHIN0219 | TMB2386 | 6.84 | 3.08 | 0.16 | -0.38 | 0.14 | 0.02 | -0.09 | -0.07 |
|  | 2 | SWU12126 | SWU12147 | 28 | TMB2386 | **SWU12343** | 5.61 | 2.50 | 0.09 | 0.34 | -0.11 | 0.01 | 0.06 | 0.04 |
|  | 28 | BNL2877 | HAU3071 | 28 | TMB2386 | **SWU12343** | 5.47 | 2.46 | 0.13 | 0.34 | 0.04 | -0.13 | 0.06 | 0.03 |
|  | 17 | NAU3765 | SWU14627 | 28 | **SWU12343** | **SWU14060** | 5.08 | 2.09 | 0.10 | -0.33 | 0.00 | -0.09 | -0.01 | 0.10 |
|  | 5 | **HAU1603** | PGML4457 | 28 | NBRI0014 | SWU12107 | 6.03 | 2.45 | 0.37 | 0.34 | 0.02 | -0.21 | 0.15 | 0.04 |
|  | 16 | SWU10627 | PGML1309 | 29 | DPL0252 | BNL3261 | 5.85 | 2.50 | 0.23 | 0.35 | 0.02 | -0.15 | -0.01 | 0.14 |
|  | 14 | PGML1568 | Gh529 | 29 | BNL3261 | CGR5111 | 5.07 | 2.40 | 0.10 | -0.34 | 0.03 | -0.08 | 0.09 | -0.04 |
|  | 6 | HAU1460 | HAU1371 | 29 | C2_0115 | ICR03107 | 6.28 | 2.79 | 0.11 | -0.37 | 0.01 | 0.09 | -0.10 | 0.00 |
|  | 24 | SWU13745 | Gh273 | 30 | BNL243 | CER0168 | 5.24 | 2.22 | 0.26 | -0.33 | -0.08 | 0.11 | 0.11 | -0.14 |
|  | 4 | ICR01729 | SWU16781 | 30 | CER0168 | SWU21718 | 5.23 | 2.28 | 0.12 | -0.33 | -0.03 | 0.07 | 0.06 | -0.11 |
|  | 11 | CAU0003 | DC40250 | 30 | CER0168 | SWU21718 | 9.47 | 4.24 | 0.15 | 0.45 | 0.06 | -0.07 | -0.11 | 0.11 |
|  | 26 | SWU17233 | **SWU17251** | 32 | NAU2140 | NAU2957 | 7.91 | 3.04 | 0.61 | -0.38 | 0.08 | 0.23 | -0.10 | -0.22 |
|  | 24 | SWU13267 | BNL1521 | 29 | DC20127 | DPL0252 | 5.63 | 2.60 | 0.23 | -0.07 | 0.03 | -0.03 | -0.01 | 0.01 |
|  | 19 | DC40122 | NAU833a | 29 | DC20127 | DPL0252 | 6.46 | 2.89 | 0.17 | 0.08 | -0.01 | 0.03 | -0.02 | 0.01 |
|  | 15 | DC40183 | DC40175 | 29 | C2_0115 | ICR03107 | 5.39 | 2.44 | 0.01 | -0.07 | 0.00 | 0.00 | 0.01 | -0.01 |
|  | 14 | TMB0071 | BNL3661 | 29 | C2_0115 | ICR03107 | 5.46 | 2.53 | 0.22 | -0.07 | 0.00 | -0.02 | 0.03 | -0.01 |
|  | 25 | SWU19411 | SWU19412 | 29 | C2_0115 | ICR03107 | 5.39 | 2.61 | 0.09 | 0.07 | 0.02 | 0.00 | -0.02 | 0.00 |
|  | 13 | **BNL1495** | **CGR5390** | 30 | BNL243 | CER0168 | 5.95 | 2.59 | 0.09 | 0.08 | 0.03 | 0.00 | -0.01 | -0.02 |
|  | 28 | NBRI0014 | SWU12107 | 32 | NAU2140 | NAU2957 | 6.25 | 3.10 | 0.25 | 0.08 | 0.01 | -0.04 | 0.02 | 0.01 |

**Supplementary Table S6-C. Main effect QTLs and environmental interactions detected for yield and yield-component traits in BC/M populations.** See footnotes in Table S6-A

| **Trait** | **Chr** | **Left marker** | **Right marker** | **LOD** | **V(A)%** | **V(AE)%** | **Effect value** | | | | |
| --- | --- | --- | --- | --- | --- | --- | --- | --- | --- | --- | --- |
|  |  |  |  |  |  |  | **A** | **AE1** | **AE2** | **AE3** | **AE4** |
| SY | 1 | **ICR03724** | ICR03725 | 2.61 | 1.13 | 0.86 | -1.15 | -1.66 | 0.60 | 0.93 | 0.13 |
|  | 4 | SWU16783 | NAU3868 | 3.03 | 0.11 | 1.27 | 0.36 | 0.15 | -1.82 | 1.57 | 0.09 |
|  | 5 | SWU20917 | NAU6240 | 4.38 | 1.93 | 0.67 | 1.49 | 0.66 | 0.14 | -1.47 | 0.67 |
|  | 6 | ICR00143 | CGR5108 | 3.67 | 1.94 | 0.07 | -1.51 | -0.19 | 0.07 | -0.32 | 0.44 |
|  | 7 | CGR5001 | CGR6586 | 2.67 | 0.00 | 1.56 | -0.02 | 1.10 | 0.76 | 0.42 | -2.27 |
|  | 14 | NAU2960 | ICR12130 | 2.58 | 0.08 | 1.11 | -0.31 | -0.08 | -0.63 | 1.84 | -1.13 |
|  | 16 | **SWU10062** | **SWU10094** | 3.54 | 1.55 | 1.22 | -1.35 | -1.96 | 1.26 | 0.16 | 0.54 |
|  | 20 | **DPL0319** | **HAU1378** | 2.58 | 0.03 | 1.25 | -0.18 | 0.90 | -1.25 | 1.46 | -1.11 |
|  | 21 | HAU0423 | CGR5806 | 4.54 | 0.15 | 2.24 | -0.42 | -1.46 | -1.35 | 2.54 | 0.28 |
|  | 24 | **Gh298** | SWU13133 | 2.64 | 0.01 | 1.00 | -0.11 | -0.19 | -1.24 | 1.71 | -0.28 |
|  | 26 | NAU2175 | **SWU17336** | 2.61 | 0.92 | 0.49 | 1.03 | -0.63 | -0.74 | 0.26 | 1.10 |
| LY | 1 | DPL0090 | Gh398 | 2.86 | 0.87 | 0.97 | -0.43 | -0.63 | -0.11 | 0.62 | 0.12 |
|  | 1 | NAU3177 | ICR03724 | 2.54 | 0.75 | 1.01 | -0.40 | -0.73 | 0.42 | 0.38 | -0.06 |
|  | 4 | SWU16783 | NAU3868 | 2.79 | 0.28 | 0.98 | 0.24 | -0.01 | -0.62 | 0.66 | -0.02 |
|  | 5 | SWU20917 | NAU6240 | 3.05 | 0.99 | 0.81 | 0.46 | 0.27 | 0.19 | -0.71 | 0.25 |
|  | 5 | HAU1603 | PGML4457 | 3.16 | 0.61 | 1.04 | -0.36 | 0.07 | -0.34 | 0.73 | -0.46 |
|  | 5 | SWU17787 | **SWU13378** | 3.24 | 0.35 | 1.32 | -0.27 | 0.90 | -0.45 | -0.20 | -0.25 |
|  | 6 | ICR00143 | CGR5108 | 3.15 | 1.64 | 0.06 | -0.59 | -0.05 | 0.11 | -0.16 | 0.10 |
|  | 10 | **BNL2960** | **SWU20511** | 3.29 | 0.00 | 1.67 | -0.01 | -0.34 | 1.00 | -0.14 | -0.52 |
|  | 16 | SWU10062 | SWU10094 | 3.37 | 1.33 | 0.98 | -0.53 | -0.78 | 0.33 | 0.15 | 0.30 |
|  | 20 | DPL0319 | HAU1378 | 2.61 | 0.03 | 1.22 | -0.08 | 0.31 | -0.54 | 0.66 | -0.43 |
|  | 21 | SWU16649 | BNL1552 | 3.19 | 0.27 | 1.17 | -0.24 | 0.40 | -0.84 | 0.27 | 0.17 |
|  | 21 | HAU0423 | CGR5806 | 3.52 | 0.04 | 1.79 | -0.09 | -0.67 | -0.33 | 0.98 | 0.02 |
|  | 22 | PGML0695 | SWU20813 | 2.95 | 0.17 | 1.23 | 0.19 | -0.08 | 0.81 | -0.14 | -0.59 |
|  | 26 | NAU2175 | SWU17336 | 3.13 | 1.34 | 0.52 | 0.53 | -0.37 | -0.14 | -0.02 | 0.53 |
| BNP | 1 | NAU3177 | ICR03724 | 2.59 | 0.46 | 0.97 | -0.16 | -0.39 | 0.07 | 0.13 | 0.19 |
|  | 5 | TMB1296 | HAU1603 | 2.68 | 0.00 | 1.32 | 0.00 | -0.08 | -0.23 | 0.45 | -0.14 |
|  | 10 | NAU2139 | SWU20689 | 2.60 | 0.96 | 0.45 | 0.23 | 0.15 | 0.16 | -0.15 | -0.16 |
|  | 16 | C2_0011B | DC20124 | 2.90 | 0.68 | 1.03 | -0.19 | 0.22 | -0.39 | 0.06 | 0.12 |
|  | 16 | SWU10056 | SWU10037 | 3.20 | 0.85 | 0.91 | -0.21 | -0.36 | 0.23 | 0.02 | 0.10 |
|  | 21 | SWU16649 | BNL1552 | 2.72 | 0.55 | 0.82 | -0.17 | 0.22 | -0.33 | -0.03 | 0.14 |
|  | 21 | HAU0423 | CGR5806 | 3.82 | 0.00 | 1.94 | -0.01 | -0.33 | -0.09 | 0.54 | -0.12 |
|  | 21 | CGR5806 | DPL0777 | 4.00 | 1.10 | 1.19 | -0.25 | 0.11 | 0.37 | -0.18 | -0.31 |
|  | 25 | SWU19815 | BNL3594 | 2.96 | 0.16 | 1.77 | 0.09 | -0.21 | -0.22 | -0.09 | 0.52 |
| BW | 1 | SWU10912 | DPL0090 | 2.94 | 1.47 | 0.12 | 0.04 | 0.02 | -0.01 | 0.00 | -0.01 |
|  | 1 | NAU3177 | ICR03724 | 2.70 | 1.16 | 0.31 | 0.03 | 0.03 | 0.00 | 0.00 | -0.02 |
|  | 1 | SWU10986 | NAU2218 | 3.22 | 1.22 | 0.35 | 0.03 | 0.00 | 0.03 | -0.01 | -0.02 |
|  | 3 | CER0028 | Gh663 | 3.22 | 1.18 | 0.56 | 0.03 | -0.03 | 0.01 | 0.03 | 0.00 |
|  | 5 | SWU20913 | Gh260 | 4.82 | 1.28 | 1.66 | 0.04 | -0.03 | -0.04 | 0.06 | 0.01 |
|  | 6 | CGR5124 | HAU1460 | 4.77 | 2.14 | 0.60 | -0.05 | 0.03 | -0.02 | -0.02 | 0.02 |
|  | 13 | **SWU22309** | **SWU22324** | 4.03 | 1.23 | 0.95 | 0.04 | -0.02 | -0.04 | 0.02 | 0.04 |
|  | 14 | Gh120 | PGML1884 | 3.37 | 1.62 | 0.32 | -0.04 | -0.02 | 0.02 | 0.00 | 0.00 |
|  | 20 | SWU20501a | CER0167 | 4.13 | 1.59 | 0.32 | -0.04 | 0.00 | 0.02 | 0.00 | -0.03 |
|  | 20 | SWU1259 | SWU20033 | 3.31 | 1.25 | 0.39 | -0.04 | -0.02 | -0.01 | 0.01 | 0.03 |
|  | 23 | SWU14807 | PGML4185 | 3.91 | 1.58 | 0.53 | 0.04 | 0.03 | -0.03 | -0.01 | 0.01 |
|  | 24 | HAU2504 | SWU13736 | 2.62 | 0.82 | 0.55 | -0.03 | 0.00 | 0.04 | -0.02 | -0.02 |
|  | 31 | SWU16753 | SWU16780 | 2.95 | 0.73 | 1.01 | 0.03 | 0.05 | -0.01 | -0.02 | -0.03 |
| LP | 3 | **SWU12765** | NAU3839 | 3.05 | 1.41 | 0.16 | 0.18 | 0.02 | -0.08 | 0.08 | -0.01 |
|  | 5 | SWU20913 | Gh260 | 10.73 | 3.26 | 2.63 | -0.27 | -0.16 | -0.30 | 0.17 | 0.29 |
|  | 5 | TMB1296 | **HAU1603** | 4.30 | 2.09 | 0.17 | -0.21 | -0.05 | 0.05 | 0.07 | -0.07 |
|  | 7 | **SWU10064** | **NAU3181** | 6.55 | 2.18 | 1.21 | -0.22 | 0.00 | -0.07 | -0.18 | 0.26 |
|  | 11 | BNL3442b | ICR01810 | 3.12 | 1.10 | 0.42 | 0.16 | -0.04 | 0.14 | 0.01 | -0.12 |
|  | 13 | PGML0014 | CGR6732 | 5.95 | 2.66 | 0.52 | -0.24 | -0.04 | 0.12 | -0.16 | 0.07 |
|  | 13 | BNL1495 | CGR5390 | 3.32 | 1.52 | 0.27 | -0.18 | -0.02 | 0.03 | 0.10 | -0.11 |
|  | 14 | TMB0071 | **BNL3661** | 2.62 | 1.02 | 0.42 | 0.15 | -0.12 | -0.06 | 0.09 | 0.09 |
|  | 15 | **DPL0182** | **SWU11691** | 2.62 | 0.56 | 0.84 | -0.11 | 0.01 | -0.17 | -0.05 | 0.21 |
|  | 19 | NAU3437 | NAU2894 | 2.81 | 1.19 | 0.21 | 0.16 | 0.09 | 0.04 | -0.06 | -0.07 |
|  | 21 | CGR5806 | DPL0777 | 4.53 | 1.36 | 0.98 | -0.17 | -0.12 | 0.21 | 0.06 | -0.15 |
|  | 22 | DPL0562 | **CAU0161** | 3.02 | 1.11 | 0.42 | 0.16 | -0.05 | -0.07 | 0.17 | -0.05 |
|  | 28 | SWU12343 | SWU14060 | 3.37 | 0.72 | 0.97 | 0.13 | 0.08 | -0.12 | -0.16 | 0.20 |

**Supplementary Table S6-D. Epistatic QTLs and environmental interactions detected for yield and yield-component traits in BC/M populations.** See footnotes in Table S6-B

| **Trait** | **Ch*i*** | **Left marker** | **Right marker** | **Ch*j*** | **Left marker** | **Right marker** | **LOD** | **V(AA)%** | **V(AAE)%** | **Effect value** | | | | |
| --- | --- | --- | --- | --- | --- | --- | --- | --- | --- | --- | --- | --- | --- | --- |
|  |  |  |  |  |  |  |  |  |  | **AA** | **AAE1** | **AAE2** | **AAE3** | **AAE4** |
| SY | 4 | SWU21617 | SWU11855 | 9 | Gh27 | SWU15194 | 5.49 | 1.29 | 1.58 | -1.22 | -2.05 | 1.45 | 0.85 | -0.24 |
|  | 9 | CGR6876 | CGR5758 | 11 | NAU3695 | DPL0050b | 5.09 | 0.21 | 2.32 | 0.57 | -0.23 | -1.32 | -1.27 | 2.81 |
|  | 12 | DPL0732 | Gh631 | 13 | PGML0014 | CGR6732 | 5.58 | 0.68 | 1.60 | -0.91 | 0.45 | -1.10 | 2.06 | -1.41 |
|  | 1 | NAU3177 | **ICR03724** | 13 | BNL1495 | CGR5390 | 5.26 | 1.01 | 1.29 | -1.09 | -1.00 | 1.98 | 0.09 | -1.07 |
|  | 15 | CGR6889 | DPL0182 | 16 | **SWU10062** | **SWU10094** | 5.03 | 1.50 | 0.92 | -1.34 | -0.81 | 1.65 | 0.28 | -1.12 |
|  | 14 | PGML1568 | Gh529 | 16 | **SWU10062** | **SWU10094** | 5.23 | 1.37 | 1.00 | 1.27 | 1.12 | 1.01 | -1.44 | -0.69 |
|  | 9 | HAU190 | HAU1618 | 18 | NAU748 | SWU22192 | 5.08 | 0.91 | 1.21 | -1.03 | -1.25 | -0.31 | 1.92 | -0.36 |
|  | 3 | SWU12732 | SWU12783 | 19 | PGML4342 | SWU14431b | 5.79 | 1.98 | 0.62 | 1.51 | 0.26 | -0.86 | -0.66 | 1.26 |
|  | 11 | NAU3695 | DPL0050b | 19 | SWU17782 | DPL0056 | 5.96 | 1.33 | 1.32 | 1.23 | -0.42 | -1.23 | -0.41 | 2.06 |
|  | 17 | CGR5576 | NAU3765 | 20 | **DPL0319** | **HAU1378** | 5.58 | 0.78 | 1.47 | -0.96 | -0.50 | -0.63 | 2.25 | -1.11 |
|  | 14 | PGML1568 | Gh529 | 21 | SWU14431a | SWU15915 | 5.74 | 0.92 | 1.35 | -1.04 | 0.55 | -1.04 | 1.81 | -1.32 |
|  | 7 | CER0036 | PGML1916 | 24 | **CGR5202** | Gh298 | 5.64 | 1.37 | 1.96 | 1.26 | 2.22 | -1.94 | -0.56 | 0.27 |
|  | 25 | SWU19129 | PGML2858 | 26 | **SWU17336** | **NAU5072** | 5.39 | 0.44 | 2.22 | 0.72 | 2.27 | 0.53 | -2.13 | -0.68 |
| LY | 6 | CGR5801 | SWU19249 | 7 | CGR5372 | SWU10205 | 5.09 | 2.33 | 0.41 | 0.70 | 0.44 | -0.16 | 0.07 | -0.35 |
|  | 4 | SWU21617 | SWU11855 | 9 | Gh27 | SWU15194 | 6.11 | 1.42 | 1.68 | -0.55 | -0.89 | 0.65 | 0.39 | -0.14 |
|  | 5 | **SWU13378** | SWU17846 | 10 | **BNL2960** | **SWU20511** | 6.62 | 0.39 | 2.56 | -0.29 | -0.74 | -0.51 | 1.21 | 0.04 |
|  | 11 | CAU0003 | DC40250 | 12 | HAU1316 | NAU3519 | 5.06 | 0.95 | 1.64 | 0.44 | 0.70 | -0.73 | -0.40 | 0.43 |
|  | 1 | NAU6367 | MUSS422 | 13 | SWU22309 | SWU22324 | 5.43 | 1.17 | 1.70 | 0.52 | -0.51 | -0.64 | 0.19 | 0.95 |
|  | 10 | ICR00093 | ICR07050 | 14 | ICR12037 | CGR5675 | 5.61 | 1.86 | 0.87 | 0.62 | -0.46 | -0.21 | -0.02 | 0.69 |
|  | 9 | SWU15511 | SWU15413 | 14 | NAU3820 | NAU2960 | 5.04 | 1.02 | 1.36 | -0.46 | -0.19 | 0.83 | 0.01 | -0.64 |
|  | 9 | HAU190 | HAU1618 | 18 | NAU748 | SWU22192 | 5.37 | 0.71 | 1.56 | -0.39 | -0.49 | -0.40 | 0.95 | -0.05 |
|  | 11 | NAU3695 | DPL0050b | 19 | SWU17782 | DPL0056 | 7.04 | 1.43 | 1.96 | 0.55 | -0.20 | -0.65 | -0.22 | 1.06 |
|  | 20 | SWU20675 | SWU20649 | 22 | CAU0161 | NAU2026 | 5.22 | 2.40 | 0.29 | 0.77 | -0.23 | -0.28 | 0.36 | 0.16 |
|  | 7 | CER0036 | PGML1916 | 24 | CGR5202 | Gh298 | 6.00 | 1.25 | 2.07 | 0.51 | 0.88 | -0.96 | -0.12 | 0.20 |
|  | 11 | NAU3390 | NAU2460 | 24 | BNL1521 | HAU2504 | 5.26 | 1.03 | 1.56 | 0.47 | 0.47 | -0.05 | -0.92 | 0.50 |
|  | 11 | CAU0003 | DC40250 | 25 | HAU1382 | SWU19848 | 5.80 | 1.93 | 1.33 | 0.63 | -0.38 | -0.38 | -0.14 | 0.90 |
|  | 20 | SWU20636 | CGR6154 | 26 | C2_0135 | PGML2321 | 5.13 | 1.19 | 1.38 | -0.52 | 0.00 | 0.56 | 0.33 | -0.88 |
|  | 19 | TMB0107 | NAU3217 | 27 | CGR6857 | ICR11205 | 5.40 | 0.01 | 1.87 | -0.05 | 0.05 | 1.01 | -0.46 | -0.60 |
| BNP | 7 | CGR5372 | SWU10205 | 10 | SWU20501b | CGR5873 | 5.52 | 2.02 | 0.86 | 0.33 | 0.03 | 0.14 | -0.35 | 0.19 |
|  | 1 | SWU10986 | NAU2218 | 11 | CER0098 | CGR5421 | 5.30 | 2.13 | 0.51 | -0.34 | 0.27 | -0.09 | 0.00 | -0.17 |
|  | 13 | SWU22413 | CGR5331 | 14 | NAU3820 | NAU2960 | 5.51 | 0.28 | 2.62 | 0.12 | -0.17 | -0.36 | -0.10 | 0.63 |
|  | 3 | SWU12732 | SWU12783 | 19 | PGML4342 | SWU14431b | 5.81 | 1.63 | 0.92 | 0.30 | -0.02 | -0.26 | -0.07 | 0.35 |
|  | 7 | SWU10785 | CER0036 | 20 | SWU20700 | CGR5548 | 5.33 | 2.50 | 0.19 | 0.38 | -0.06 | 0.13 | 0.05 | -0.13 |
|  | 18 | DC40150 | ICR02849 | 26 | Gh64 | SWU17257 | 5.98 | 1.99 | 0.90 | -0.35 | -0.13 | 0.00 | 0.40 | -0.27 |
|  | 26 | SWU17251 | C2_0135 | 26 | SWU0514 | SWU18488 | 5.29 | 1.16 | 1.03 | -0.27 | 0.32 | 0.22 | -0.20 | -0.34 |
|  | 13 | NAU2893 | Gh157 | 26 | SWU18681 | SWU0598 | 5.51 | 0.12 | 2.38 | 0.08 | 0.06 | 0.39 | -0.58 | 0.14 |
| BW | 10 | Gh320 | HAU0635 | 13 | NAU3468 | **SWU22309** | 5.92 | 2.85 | 0.27 | 0.05 | 0.01 | -0.02 | 0.02 | -0.01 |
|  | 6 | SWU19541 | CGR5801 | 13 | **SWU22309** | **SWU22324** | 6.38 | 3.33 | 0.70 | -0.06 | 0.02 | -0.01 | -0.04 | 0.03 |
|  | 11 | PGML2202 | CGR6580 | 25 | Gh220 | SWU19434 | 5.06 | 2.61 | 0.25 | -0.05 | 0.00 | 0.00 | -0.02 | 0.02 |
| LP | 1 | SWU11191 | BNL2827b | 5 | SWU20917 | NAU6240 | 5.14 | 1.89 | 0.72 | -0.21 | 0.05 | 0.15 | -0.17 | -0.02 |
|  | 5 | SWU17787 | SWU13378 | 6 | SWU19541 | CGR5801 | 5.60 | 1.89 | 0.65 | 0.20 | 0.07 | -0.19 | 0.01 | 0.12 |
|  | 2 | SWU11950 | TMB1268 | 7 | **SWU10064** | **NAU3181** | 5.54 | 1.26 | 0.97 | -0.17 | 0.00 | 0.05 | 0.18 | -0.23 |
|  | 4 | SWU16782 | SWU16783 | 10 | Gh320 | HAU0635 | 5.37 | 1.69 | 0.49 | -0.20 | -0.07 | 0.09 | 0.12 | -0.14 |
|  | 1 | BNL2827a | NAU6367 | 10 | NAU2139 | SWU20689 | 5.41 | 2.31 | 0.24 | -0.23 | -0.03 | 0.11 | 0.01 | -0.09 |
|  | 4 | SWU21415 | BNL530 | 11 | ICR01787 | NAU3695 | 5.18 | 1.79 | 0.58 | -0.19 | 0.10 | 0.14 | -0.13 | -0.11 |
|  | 7 | NAU1357 | SWU10067 | 13 | SWU22413 | CGR5331 | 6.93 | 3.16 | 0.29 | 0.26 | 0.02 | -0.12 | 0.10 | 0.00 |
|  | 11 | NAU3695 | DPL0050b | 13 | SWU22413 | CGR5331 | 5.09 | 1.30 | 1.16 | 0.17 | 0.14 | -0.23 | -0.06 | 0.15 |
|  | 13 | NAU3468 | SWU22309 | 13 | HAU2558 | NAU2893 | 5.20 | 2.04 | 0.03 | -0.24 | -0.02 | 0.08 | -0.11 | 0.05 |
|  | 1 | ICR03724 | ICR03725 | 13 | DPL0894 | SWU10800 | 5.80 | 2.78 | 0.03 | 0.25 | 0.00 | 0.02 | -0.04 | 0.02 |
|  | 7 | PGML1916 | SWU10864 | 14 | **BNL3661** | PGML2498 | 5.27 | 1.44 | 0.91 | 0.19 | -0.10 | 0.22 | 0.05 | -0.16 |
|  | 10 | BNL2960 | SWU20511 | 15 | **DPL0182** | **SWU11691** | 5.02 | 1.95 | 0.21 | -0.21 | 0.05 | -0.12 | 0.06 | 0.00 |
|  | 3 | CER0028 | Gh663 | 15 | **DPL0182** | **SWU11691** | 5.63 | 1.92 | 0.56 | 0.20 | 0.11 | -0.18 | 0.01 | 0.07 |
|  | 5 | **HAU1603** | PGML4457 | 16 | Gh56 | NAU5120 | 5.22 | 1.04 | 1.41 | 0.15 | 0.09 | -0.30 | 0.10 | 0.11 |
|  | 11 | NAU2152 | NAU5428 | 17 | SWU12818 | CGR5576 | 5.48 | 2.08 | 0.50 | 0.22 | -0.03 | -0.16 | 0.10 | 0.10 |
|  | 1 | NAU6367 | MUSS422 | 17 | NAU3765 | SWU14627 | 5.94 | 2.23 | 0.53 | 0.24 | 0.06 | -0.19 | 0.08 | 0.06 |
|  | 2 | SWU11887 | SWU11976 | 17 | SWU14627 | CGR5871 | 5.25 | 1.39 | 0.92 | -0.17 | 0.02 | 0.21 | -0.05 | -0.19 |
|  | 17 | NAU3765 | SWU14627 | 19 | TMB0107 | NAU3217 | 6.17 | 2.16 | 0.62 | 0.23 | 0.21 | -0.09 | -0.05 | -0.08 |
|  | 16 | HAU1129 | NAU2984 | 20 | SWU20501a | CER0167 | 7.37 | 2.21 | 0.88 | -0.22 | -0.07 | 0.22 | 0.01 | -0.16 |
|  | 21 | Gh451 | SWU16489 | 21 | SWU16488 | SWU16138 | 5.57 | 2.73 | 0.07 | -0.26 | -0.02 | -0.04 | 0.02 | 0.03 |
|  | 3 | SWU12783 | SWU12819 | 21 | SWU15915 | SWU0189 | 5.97 | 2.55 | 0.17 | 0.24 | 0.02 | -0.09 | 0.07 | 0.00 |
|  | 18 | SWU22281 | SWU21800 | 22 | **CAU0161** | NAU2026 | 5.77 | 2.02 | 0.41 | 0.23 | 0.05 | -0.11 | -0.08 | 0.15 |
|  | 17 | NAU3765 | SWU14627 | 24 | CGR5202 | Gh298 | 5.85 | 1.33 | 1.43 | 0.18 | 0.14 | -0.29 | -0.03 | 0.18 |
|  | 21 | SWU15915 | SWU0189 | 25 | SWU19129 | PGML2858 | 5.28 | 2.49 | 0.04 | 0.24 | 0.02 | -0.05 | 0.02 | 0.01 |
|  | 1 | SWU10912 | DPL0090 | 25 | SWU19144 | Gh220 | 5.35 | 1.80 | 0.57 | -0.20 | 0.01 | 0.15 | 0.01 | -0.16 |
|  | 7 | NAU1357 | SWU10067 | 25 | SWU19144 | Gh220 | 5.23 | 2.20 | 0.13 | -0.22 | -0.04 | 0.01 | 0.09 | -0.05 |
|  | 21 | SWU15915 | SWU0189 | 26 | SWU17336 | NAU5072 | 6.18 | 2.17 | 0.44 | -0.22 | 0.01 | 0.16 | -0.10 | -0.07 |
|  | 3 | SWU12819 | **SWU12765** | 26 | DPL0491 | Gh64 | 5.30 | 1.39 | 1.03 | 0.17 | 0.16 | -0.16 | -0.15 | 0.14 |
|  | 14 | SWU14224 | DPL0565 | 26 | SWU0514 | SWU18488 | 5.66 | 1.89 | 0.69 | -0.20 | -0.09 | 0.13 | 0.11 | -0.14 |
|  | 13 | SWU22413 | CGR5331 | 26 | SWU18488 | SWU18672 | 6.30 | 1.67 | 1.20 | -0.19 | -0.14 | 0.25 | 0.03 | -0.14 |
|  | 20 | SWU20700 | CGR5548 | 26 | SWU18488 | SWU18672 | 6.28 | 2.18 | 0.50 | -0.23 | -0.15 | 0.14 | 0.08 | -0.07 |
|  | 12 | DPL0303 | COT107 | 29 | DPL0252 | BNL3261 | 5.78 | 1.63 | 0.85 | -0.19 | -0.01 | 0.11 | 0.12 | -0.22 |
|  | 9 | Gh111 | Gh27 | 30 | BNL243 | CER0168 | 5.36 | 2.08 | 0.48 | -0.21 | 0.03 | 0.09 | -0.17 | 0.06 |
|  | 11 | CGR6580 | SWU15972 | 30 | BNL243 | CER0168 | 5.16 | 2.06 | 0.22 | 0.21 | -0.03 | 0.10 | -0.09 | 0.01 |
|  | 4 | SWU16782 | SWU16783 | 30 | CER0168 | SWU21718 | 6.97 | 3.02 | 0.37 | -0.26 | -0.03 | 0.15 | -0.02 | -0.10 |
|  | 26 | Gh64 | SWU17257 | 32 | NAU2140 | NAU2957 | 5.23 | 1.84 | 0.67 | -0.20 | 0.06 | 0.13 | 0.02 | -0.21 |

**Supplementary Table S6-E Main effect QTLs and environmental interactions detected for yield and yield-component traits in MPH-M datasets.** See footnotes in Table S6-A.

| **Trait** | **Chr** | **Left marker** | **Right marker** | **LOD** | **V(A)%** | **V(AE)%** | **Effect value** | | | | |
| --- | --- | --- | --- | --- | --- | --- | --- | --- | --- | --- | --- |
|  |  |  |  |  |  |  | **A** | **AE1** | **AE2** | **AE3** | **AE4** |
| SY | 1 | SWU11191 | BNL2827b | 2.57 | 0.85 | 0.52 | -0.94 | 0.34 | -0.17 | 0.91 | -1.08 |
|  | 6 | CGR5801 | SWU19249 | 2.71 | 1.02 | 0.98 | -1.02 | 0.38 | -1.71 | 0.83 | 0.50 |
|  | 11 | **NAU3390** | **NAU2460** | 3.03 | 0.37 | 1.22 | -0.62 | 1.00 | 1.16 | -0.69 | -1.47 |
|  | 12 | **Gh631** | HAU1321 | 3.67 | 0.90 | 1.61 | 0.95 | 0.13 | 1.68 | 0.11 | -1.92 |
|  | 21 | SWU16573 | Gh451 | 4.00 | 0.59 | 2.61 | 0.77 | 0.25 | 2.48 | -1.94 | -0.79 |
|  | 21 | HAU0423 | CGR5806 | 3.29 | 0.75 | 0.67 | 0.88 | 1.33 | -0.22 | -0.98 | -0.12 |
|  | 24 | CGR5202 | **Gh298** | 2.78 | 0.10 | 1.39 | 0.32 | 1.23 | 1.15 | -1.18 | -1.21 |
| LY | 11 | **NAU3390** | **NAU2460** | 2.95 | 0.32 | 1.28 | -0.24 | 0.48 | 0.46 | -0.32 | -0.62 |
|  | 12 | Gh631 | HAU1321 | 3.37 | 0.78 | 1.53 | 0.37 | -0.03 | 0.69 | 0.11 | -0.78 |
|  | 13 | PGML0014 | CGR6732 | 2.56 | 0.05 | 1.20 | -0.09 | -0.67 | 0.65 | 0.10 | -0.09 |
|  | 14 | NAU3820 | NAU2960 | 2.51 | 0.50 | 0.98 | -0.30 | 0.57 | -0.12 | -0.59 | 0.13 |
|  | 21 | SWU16573 | Gh451 | 3.97 | 0.45 | 2.57 | 0.28 | 0.10 | 1.02 | -0.85 | -0.27 |
|  | 21 | **HAU0423** | **CGR5806** | 4.08 | 1.05 | 0.74 | 0.44 | 0.63 | -0.18 | -0.30 | -0.15 |
|  | 25 | SWU19763 | SWU19129 | 2.80 | 0.03 | 1.47 | 0.07 | 0.20 | -0.39 | -0.56 | 0.74 |
| BNP | 11 | NAU2152 | NAU5428 | 2.82 | 0.48 | 1.16 | -0.15 | 0.33 | -0.21 | 0.12 | -0.24 |
|  | 11 | PGML2202 | CGR6580 | 3.09 | 0.20 | 1.68 | -0.10 | -0.12 | -0.19 | -0.18 | 0.49 |
|  | 14 | **NAU2960** | ICR12130 | 2.52 | 0.73 | 0.89 | -0.19 | 0.29 | 0.01 | -0.30 | 0.01 |
|  | 16 | SWU10094 | SWU10060 | 2.83 | 0.34 | 1.30 | -0.13 | -0.23 | -0.24 | 0.35 | 0.12 |
|  | 19 | SWU18015 | SWU17990 | 3.87 | 0.00 | 2.39 | 0.01 | -0.34 | 0.11 | -0.28 | 0.51 |
|  | 25 | SWU19430 | PGML1219 | 3.54 | 0.72 | 1.52 | 0.19 | -0.02 | -0.44 | 0.22 | 0.23 |
| BW | 13 | NAU3468 | SWU22309 | 2.56 | 0.18 | 1.26 | -0.01 | -0.01 | 0.01 | -0.05 | 0.05 |
|  | 16 | PGML1709 | SWU10627 | 2.77 | 0.17 | 1.00 | -0.01 | 0.00 | 0.04 | 0.01 | -0.05 |
|  | 25 | Gh220 | SWU19434 | 3.30 | 1.43 | 0.25 | -0.04 | 0.01 | -0.02 | 0.01 | 0.00 |
|  | 26 | SWU17251 | C2_0135 | 2.68 | 1.23 | 0.62 | 0.04 | 0.03 | -0.03 | 0.01 | -0.01 |
|  | 28 | SHIN0219 | TMB2386 | 3.47 | 0.25 | 1.67 | 0.02 | 0.02 | -0.01 | 0.05 | -0.06 |
| LP | 2 | SWU12147 | CGR6695 | 2.99 | 1.51 | 0.37 | 0.15 | 0.11 | 0.00 | -0.02 | -0.09 |
|  | 5 | **PGML4350** | **SWU17781** | 2.60 | 1.30 | 0.14 | -0.14 | 0.06 | -0.04 | 0.03 | -0.05 |
|  | 7 | **PGML1916** | **SWU10864** | 3.27 | 0.00 | 1.88 | -0.01 | -0.20 | -0.02 | -0.06 | 0.27 |
|  | 11 | SWU15972 | TMB0628 | 2.54 | 1.21 | 0.32 | -0.14 | -0.05 | -0.06 | 0.01 | 0.11 |
|  | 13 | NAU2893 | Gh157 | 3.21 | 1.57 | 0.47 | 0.16 | 0.09 | 0.00 | 0.05 | -0.14 |
|  | 14 | PGML4763 | SWU13909 | 4.04 | 0.91 | 1.59 | 0.12 | 0.22 | -0.01 | -0.22 | 0.01 |
|  | 14 | NAU2960 | ICR12130 | 3.08 | 0.40 | 1.51 | -0.08 | 0.00 | 0.24 | -0.17 | -0.07 |
|  | 17 | HAU1413 | CGR6017 | 2.56 | 0.52 | 0.89 | 0.09 | 0.01 | 0.06 | -0.19 | 0.12 |
|  | 22 | SWU21635 | SWU21646 | 2.77 | 0.13 | 1.86 | -0.05 | 0.22 | 0.02 | -0.25 | 0.01 |
|  | 27 | SWU11038 | SWU11384 | 3.18 | 1.28 | 0.82 | 0.14 | 0.11 | -0.16 | 0.10 | -0.05 |

**Supplementary Table S6-F. Epistatic QTLs and environmental interactions detected for yield and yield-component traits in MPH-M dataset.** See footnotes in Table S6-B.

| **Trait** | **Ch*i*** | **Left marker** | **Right marker** | **Ch*j*** | **Left marker** | **Right marker** | **LOD** | **V(AA)%** | **V(AAE)%** | **Effect value** | | | | |
| --- | --- | --- | --- | --- | --- | --- | --- | --- | --- | --- | --- | --- | --- | --- |
|  |  |  |  |  |  |  |  |  |  | **AA** | **AAE1** | **AAE2** | **AAE3** | **AAE4** |
| SY | 9 | Gh27 | SWU15194 | 12 | DPL0732 | **Gh631** | 5.45 | 1.25 | 1.53 | 1.13 | -1.69 | 0.59 | 1.67 | -0.58 |
|  | 1 | NAU6367 | MUSS422 | 13 | DPL0535 | CER0165 | 5.09 | 1.38 | 1.22 | 1.22 | 0.97 | 0.44 | -1.96 | 0.55 |
|  | 10 | Gh320 | HAU0635 | 16 | SWU10062 | SWU10094 | 5.21 | 0.65 | 2.23 | -0.81 | 1.95 | -2.31 | 0.12 | 0.24 |
|  | 3 | SWU12765 | NAU3839 | 19 | SWU17897 | CGR5539 | 5.55 | 1.23 | 1.59 | 1.13 | -1.55 | 1.87 | -0.65 | 0.33 |
|  | 7 | PGML1916 | SWU10864 | 19 | TMB0107 | NAU3217 | 5.24 | 0.23 | 2.53 | -0.53 | 1.33 | -2.14 | 2.09 | -1.28 |
|  | 14 | ICR12130 | DPL0502 | 21 | SWU14431a | SWU15915 | 5.33 | 0.04 | 2.25 | -0.21 | 2.01 | 0.96 | -1.23 | -1.74 |
|  | 16 | PGML1709 | SWU10627 | 23 | PGML4186 | NAU3100 | 5.00 | 1.34 | 1.52 | 1.17 | -0.24 | 1.70 | -1.78 | 0.32 |
|  | 16 | SWU20341 | DPL0897 | 24 | **Gh298** | SWU13133 | 5.19 | 1.23 | 1.51 | 1.14 | 0.36 | 1.67 | -1.83 | -0.20 |
|  | 26 | DPL0491 | Gh64 | 26 | Gh64 | SWU17257 | 5.56 | 0.87 | 1.17 | 1.21 | 1.13 | 1.11 | -3.34 | 1.09 |
|  | 26 | SWU17251 | C2_0135 | 27 | ICR11205 | DPL0847a | 5.04 | 1.80 | 0.65 | -1.36 | 1.40 | -0.70 | -0.46 | -0.23 |
|  | 11 | **NAU3390** | **NAU2460** | 28 | TMB2386 | SWU12343 | 6.51 | 0.93 | 2.28 | -1.00 | 1.16 | -1.95 | 1.78 | -0.99 |
|  | 7 | CGR5001 | CGR6586 | 30 | BNL243 | CER0168 | 5.31 | 0.92 | 2.01 | 0.98 | -0.06 | 2.25 | -1.78 | -0.42 |
|  | 2 | SWU11976 | SWU11950 | 31 | SWU16676 | SWU16755 | 5.81 | 0.03 | 2.67 | -0.17 | -1.39 | -0.41 | 2.82 | -1.03 |
| LY | 3 | SWU12765 | NAU3839 | 19 | SWU17897 | CGR5539 | 5.28 | 1.21 | 1.34 | 0.47 | -0.66 | 0.67 | -0.20 | 0.19 |
|  | 7 | PGML1916 | SWU10864 | 19 | TMB0107 | NAU3217 | 5.43 | 0.17 | 2.67 | -0.19 | 0.50 | -0.92 | 0.96 | -0.53 |
|  | 21 | **HAU0423** | **CGR5806** | 26 | SWU17432 | SWU17395 | 5.04 | 1.24 | 1.34 | 0.49 | -0.08 | 0.70 | -0.70 | 0.09 |
|  | 26 | DPL0491 | Gh64 | 26 | Gh64 | SWU17257 | 5.75 | 0.66 | 1.64 | 0.48 | 0.48 | 0.44 | -1.43 | 0.52 |
|  | 3 | SWU12732 | SWU12783 | 27 | SWU11384 | ICR11885 | 5.57 | 1.15 | 1.76 | 0.46 | -0.70 | 0.74 | -0.40 | 0.36 |
|  | 26 | SWU17251 | C2_0135 | 27 | ICR11205 | DPL0847a | 5.11 | 1.65 | 0.71 | -0.55 | 0.62 | -0.30 | -0.20 | -0.12 |
|  | 11 | **NAU3390** | **NAU2460** | 28 | TMB2386 | SWU12343 | 6.60 | 0.87 | 2.17 | -0.41 | 0.47 | -0.73 | 0.76 | -0.50 |
|  | 2 | SWU11950 | TMB1268 | 31 | SWU16676 | SWU16755 | 5.75 | 0.03 | 2.77 | 0.08 | -0.59 | -0.03 | 1.18 | -0.56 |
| BNP | 3 | SWU12783 | SWU12819 | 3 | SWU12765 | NAU3839 | 5.43 | 0.01 | 2.58 | -0.03 | -0.34 | 0.43 | -0.43 | 0.33 |
|  | 1 | SWU0077 | HAU1417 | 6 | CGR5108 | ICR03206 | 5.04 | 0.00 | 2.77 | 0.00 | -0.25 | -0.08 | 0.64 | -0.31 |
|  | 13 | SWU22374 | HAU2857 | 15 | DC40183 | DC40175 | 5.35 | 0.37 | 2.95 | -0.14 | 0.23 | 0.48 | -0.51 | -0.20 |
|  | 14 | NAU3820 | **NAU2960** | 17 | SWU12818 | CGR5576 | 5.09 | 0.60 | 2.23 | -0.17 | 0.11 | 0.32 | -0.56 | 0.13 |
|  | 14 | PGML1368 | PGML1568 | 26 | SWU17467 | SWU17419 | 5.42 | 0.05 | 2.86 | 0.05 | 0.21 | -0.39 | 0.51 | -0.32 |
|  | 13 | NAU2893 | Gh157 | 26 | SWU18681 | SWU0598 | 5.81 | 0.06 | 3.07 | 0.05 | -0.59 | -0.11 | 0.32 | 0.38 |
|  | 16 | ICR00010 | SWU10038 | 29 | DC20127 | DPL0252 | 5.65 | 0.00 | 3.01 | -0.01 | -0.37 | 0.18 | -0.36 | 0.54 |
| LP | 5 | **PGML4350** | **SWU17781** | 7 | **PGML1916** | **SWU10864** | 5.13 | 1.15 | 1.72 | 0.15 | 0.21 | -0.11 | 0.12 | -0.22 |
|  | 3 | SWU12765 | NAU3839 | 14 | PGML1568 | Gh529 | 5.42 | 1.74 | 1.03 | -0.16 | -0.05 | 0.12 | -0.18 | 0.11 |
|  | 7 | NAU3181 | SHIN0376 | 16 | CGR6802 | HAU1129 | 5.26 | 1.78 | 0.99 | -0.17 | 0.00 | 0.03 | -0.19 | 0.16 |
|  | 7 | **PGML1916** | **SWU10864** | 17 | ICR03391 | SWU12838a | 5.65 | 1.18 | 1.48 | -0.15 | -0.15 | 0.22 | -0.18 | 0.11 |
|  | 7 | CER0036 | **PGML1916** | 22 | PGML0695 | SWU20813 | 5.02 | 0.02 | 2.10 | -0.02 | -0.01 | 0.22 | 0.06 | -0.27 |
|  | 5 | SWU17787 | SWU13378 | 24 | Gh298 | SWU13133 | 6.08 | 2.53 | 0.44 | -0.20 | -0.01 | 0.08 | -0.13 | 0.06 |
|  | 17 | NAU3765 | SWU14627 | 25 | SWU19129 | PGML2858 | 6.12 | 2.79 | 0.70 | -0.21 | -0.04 | 0.13 | -0.15 | 0.06 |
|  | 4 | BNL530 | SWU21485 | 26 | SWU17233 | SWU17251 | 5.62 | 0.67 | 2.18 | 0.10 | 0.16 | 0.09 | 0.06 | -0.31 |

**Supplementary Table S7-A. Main effect QTLs and environmental interactions detected for yield and yield-component traits in RIL-P populations.** The result detected by software ICIMapping 4.1. E1-E3: three environments; E1, Cangzhou in 2015; E2, Handan in 2016; E3, Cangzhou in 2016. Chr represented the linkage group number of the loci being tested in the analysis. V(A)% and V(AE)%: percentage of the total phenotypic variation explained by one QTL and by QTL × environmentat the current scanning position, respectively. Effect value: A, the total genetic effect value; AE, the effect value by QTL × environment; AE1, AE2 and AE3: the effect value under environments of E1, E2 and E3, respectively. Bold figures indicated common QTLs between E-QTLs by QEs and M-QTLs by QEs in the population.

| Trait | Chr | Left marker | Right marker | LOD | V(A)% | V(AE)% | Effect value | | | |
| --- | --- | --- | --- | --- | --- | --- | --- | --- | --- | --- |
|  |  |  |  |  |  |  | A | AE1 | AE2 | AE3 |
| SY | 1 | NAU3177 | ICR03724 | 2.51 | 1.55 | 0.28 | -1.90 | 0.96 | 0.07 | -1.03 |
|  | 2 | **SWU11887** | **SWU11976** | 3.75 | 2.21 | 0.62 | 2.24 | 0.84 | 0.85 | -1.69 |
|  | 2 | **SWU11976** | SWU11950 | 2.82 | 1.45 | 0.71 | 1.83 | 1.10 | 0.69 | -1.78 |
|  | 4 | SWU18876 | **SWU12672** | 2.70 | 1.85 | 0.13 | -2.08 | 0.78 | -0.37 | -0.41 |
|  | 5 | SWU20917 | NAU6240 | 2.76 | 1.91 | 0.06 | 2.09 | -0.51 | 0.20 | 0.31 |
|  | 20 | **SWU20246** | **SWU20501a** | 7.39 | 5.52 | 0.45 | -3.55 | -0.92 | -0.48 | 1.40 |
|  | 21 | ICR08249 | SWU15733 | 4.28 | 2.63 | 0.80 | -2.46 | -1.85 | 0.48 | 1.37 |
|  | 22 | **CAU0161** | NAU2026 | 2.59 | 1.97 | 0.01 | -2.28 | -0.15 | -0.11 | 0.25 |
|  | 28 | SHIN0219 | TMB2386 | 2.69 | 1.87 | 0.03 | 2.07 | 0.21 | 0.17 | -0.38 |
|  | 29 | C2_0115 | ICR03107 | 3.13 | 1.50 | 1.00 | 1.85 | 2.07 | -1.48 | -0.59 |
| LY | 2 | SWU11889 | **SWU11887** | 6.22 | 3.74 | 1.29 | 1.16 | 0.61 | 0.34 | -0.95 |
|  | 4 | SWU18876 | SWU12672 | 2.99 | 2.24 | 0.04 | -0.90 | 0.12 | 0.05 | -0.16 |
|  | 20 | **SWU20246** | **SWU20501a** | 4.78 | 3.50 | 0.28 | -1.12 | -0.38 | -0.03 | 0.40 |
|  | 21 | CGR5217 | BNL3442a | 3.59 | 2.24 | 0.50 | -0.90 | -0.46 | -0.10 | 0.57 |
|  | 22 | DPL0562 | CAU0161 | 2.51 | 1.96 | 0.00 | -0.91 | -0.03 | 0.06 | -0.03 |
|  | 24 | SWU13267 | BNL1521 | 2.52 | 1.72 | 0.14 | 0.79 | 0.31 | -0.21 | -0.09 |
|  | 25 | SWU19430 | PGML1219 | 2.75 | 1.83 | 0.34 | -0.81 | -0.16 | 0.48 | -0.32 |
|  | 28 | SHIN0219 | **TMB2386** | 2.71 | 1.95 | 0.09 | 0.84 | 0.25 | -0.14 | -0.10 |
| BNP | 1 | SWU10912 | DPL0090 | 3.80 | 1.60 | 0.82 | -0.39 | 0.33 | 0.02 | -0.36 |
|  | 4 | SWU18876 | SWU12672 | 3.09 | 1.57 | 0.22 | -0.39 | -0.07 | 0.20 | -0.14 |
|  | 5 | HAU1603 | PGML4457 | 2.62 | 0.01 | 2.15 | -0.03 | -0.45 | 0.63 | -0.18 |
|  | 9 | HAU1618 | NAU2873 | 3.04 | 0.01 | 2.14 | 0.04 | 0.02 | 0.55 | -0.57 |
|  | 15 | DPL0182 | SWU11691 | 4.72 | 3.16 | 0.34 | -0.55 | 0.25 | -0.11 | -0.15 |
|  | 20 | SWU20246 | SWU20501a | 3.56 | 1.90 | 0.87 | -0.43 | -0.31 | -0.08 | 0.39 |
|  | 21 | CGR5217 | BNL3442a | 3.13 | 1.62 | 0.52 | -0.40 | -0.32 | 0.14 | 0.18 |
|  | 27 | CGR6857 | ICR11205 | 2.53 | 1.33 | 1.02 | 0.36 | -0.39 | 0.39 | 0.00 |
|  | 29 | C2_0115 | ICR03107 | 3.65 | 0.02 | 2.28 | -0.04 | 0.67 | -0.33 | -0.34 |
| BW | 2 | SWU11887 | SWU11976 | 2.55 | 1.42 | 0.40 | 0.05 | 0.04 | -0.03 | 0.00 |
|  | 4 | BNL530 | SWU21485 | 5.20 | 3.96 | 0.24 | 0.09 | 0.03 | 0.00 | -0.03 |
|  | 5 | **SWU20917** | **NAU6240** | 10.55 | 8.51 | 0.02 | 0.13 | 0.00 | 0.01 | -0.01 |
|  | 6 | HAU1460 | HAU1371 | 3.36 | 2.16 | 0.28 | -0.07 | -0.03 | 0.03 | -0.01 |
|  | 11 | CGR6525 | ICR01787 | 2.66 | 1.28 | 0.85 | -0.05 | 0.05 | -0.06 | 0.01 |
|  | 16 | **SWU10094** | SWU10060 | 3.44 | 2.21 | 0.12 | 0.07 | -0.02 | 0.00 | 0.02 |
|  | 16 | **SWU10038** | **ICR00016** | 4.03 | 3.04 | 0.02 | 0.08 | 0.01 | 0.00 | -0.01 |
|  | 19 | **NAU3437** | **NAU2894** | 3.49 | 2.62 | 0.18 | -0.07 | 0.00 | -0.02 | 0.02 |
|  | 19 | SWU17897 | CGR5539 | 3.53 | 2.73 | 0.07 | -0.08 | 0.00 | -0.01 | 0.02 |
|  | 20 | **CGR6154** | SWU20246 | 2.51 | 1.64 | 0.14 | -0.06 | -0.02 | 0.02 | -0.01 |
|  | 20 | **SWU20501a** | **CER0167** | 2.95 | 2.07 | 0.21 | -0.07 | -0.03 | 0.02 | 0.01 |
| LP | 1 | NAU2218 | SWU11191 | 2.62 | 1.64 | 0.07 | -0.31 | 0.00 | -0.08 | 0.08 |
|  | 2 | SWU12025 | SWU11889 | 4.38 | 2.62 | 0.14 | 0.39 | 0.08 | 0.05 | -0.12 |
|  | 5 | SWU20917 | NAU6240 | 21.39 | 14.88 | 0.08 | -0.92 | 0.06 | 0.04 | -0.09 |
|  | 5 | TMB1296 | HAU1603 | 3.18 | 1.24 | 0.63 | -0.27 | -0.17 | 0.27 | -0.10 |
|  | 7 | NAU3181 | SHIN0376 | 2.58 | 1.56 | 0.10 | -0.30 | -0.05 | -0.06 | 0.11 |
|  | 9 | SWU15511 | SWU15413 | 2.99 | 1.73 | 0.09 | 0.31 | -0.08 | -0.02 | 0.10 |
|  | 11 | BNL3442b | ICR01810 | 3.16 | 1.94 | 0.01 | 0.33 | -0.02 | 0.03 | 0.00 |
|  | 13 | PGML3887 | BNL2449 | 3.07 | 1.60 | 0.32 | -0.31 | 0.16 | 0.00 | -0.17 |
|  | 13 | BNL1495 | CGR5390 | 9.04 | 5.18 | 0.92 | -0.55 | -0.02 | -0.27 | 0.29 |
|  | 14 | NAU3308 | HAU1057 | 4.50 | 2.74 | 0.21 | 0.40 | -0.08 | 0.16 | -0.08 |
|  | 14 | NAU3820 | NAU2960 | 2.60 | 1.46 | 0.20 | 0.29 | 0.11 | 0.03 | -0.14 |
|  | 14 | CIR228 | BNL2485 | 2.67 | 1.60 | 0.10 | 0.30 | -0.01 | 0.10 | -0.09 |
|  | 19 | NAU3437 | NAU2894 | 4.50 | 2.74 | 0.06 | 0.40 | -0.08 | 0.02 | 0.06 |
|  | 20 | CGR6154 | SWU20246 | 3.50 | 2.09 | 0.15 | 0.35 | 0.05 | 0.08 | -0.13 |
|  | 20 | CER0167 | SWU20064 | 3.80 | 2.06 | 0.37 | 0.34 | 0.07 | 0.13 | -0.20 |
|  | 22 | DPL0562 | CAU0161 | 3.17 | 1.80 | 0.25 | 0.33 | -0.05 | 0.17 | -0.12 |
|  | 25 | SWU19430 | PGML1219 | 3.02 | 1.77 | 0.07 | -0.32 | -0.05 | 0.09 | -0.04 |
|  | 26 | SWU18672 | SWU18681 | 2.80 | 1.70 | 0.05 | -0.31 | 0.07 | -0.04 | -0.03 |
|  | 27 | CGR5056 | DC40052 | 3.64 | 1.89 | 0.44 | -0.33 | -0.07 | -0.15 | 0.22 |

**Supplementary Table S7-B. Epistatic QTLs and environmental interactions detected for yield and yield-component traits in RIL-P populations.** The result detected by software ICIMapping 4.1. E1-E3, bold figures: see footnotes in Table S7. Ch*i* and Ch*j* represented the linkage group number of the loci being tested in the analysis. V(AA)% and V(AAE)%: percentage of the total phenotypic variation, explaining by epistatic QTLs and by epistatic QTLs × the environment, respectively. Effect value: AA, the total effect value of epistatic QTLs; AAEl, AAE2 and AAE3 indicated the effect values by epistatic QTLs × environment in E1, E2 and E3, respectively.

| **Trait** | **Ch*i*** | **Left marker** | | **Right marker** | | **Ch*j*** | **Left marker** | **Right marker** | | **LOD** | **V(AA)%** | **V(AAE)%** | **Effect value** | | | |
| --- | --- | --- | --- | --- | --- | --- | --- | --- | --- | --- | --- | --- | --- | --- | --- | --- |
|  |  |  | |  | |  |  |  | |  |  |  | **AA** | **AAE1** | **AAE2** | **AAE3** |
| SY | 1 | SWU10986 | NAU2218 | | 2 | | **SWU11887** | **SWU11976** | 5.36 | | 3.34 | 0.24 | -2.78 | 0.73 | 0.29 | -1.02 |
|  | 4 | SWU16782 | SWU16783 | | 5 | | HAU1603 | PGML4457 | 5.02 | | 3.22 | 0.50 | -2.72 | 0.13 | -1.38 | 1.25 |
|  | 6 | ICR03206 | NAU896 | | 7 | | Gh474 | SWU10785 | 6.48 | | 3.76 | 0.43 | -3.01 | -0.03 | -1.21 | 1.23 |
|  | 9 | NAU3966 | SWU15157 | | 10 | | CGR5873 | ICR00093 | 7.23 | | 4.81 | 0.06 | 3.36 | 0.48 | -0.03 | -0.46 |
|  | 4 | SWU16783 | NAU3868 | | 11 | | NAU3390 | NAU2460 | 6.98 | | 4.57 | 0.35 | -3.28 | -0.56 | -0.67 | 1.23 |
|  | 10 | HAU0635 | NAU2139 | | 13 | | BNL1495 | CGR5390 | 6.40 | | 3.54 | 0.69 | 2.87 | -0.80 | 1.79 | -0.99 |
|  | 13 | SWU22309 | SWU22324 | | 13 | | DPL0894 | SWU10800 | 5.99 | | 3.88 | 0.47 | -3.18 | 0.89 | 0.75 | -1.64 |
|  | 5 | DPL0022 | SWU17787 | | 16 | | NAU862 | CGR6802 | 5.28 | | 2.94 | 0.87 | 2.61 | -1.99 | 0.76 | 1.23 |
|  | 16 | SWU10266 | DC40065 | | 16 | | SWU10038 | ICR00016 | 5.31 | | 3.46 | 0.09 | -2.84 | -0.05 | -0.58 | 0.63 |
|  | 12 | DPL0303 | COT107 | | 17 | | NAU3765 | SWU14627 | 5.77 | | 3.40 | 0.49 | -2.83 | 0.56 | 0.95 | -1.51 |
|  | 10 | NAU2139 | SWU20689 | | 18 | | NAU748 | SWU22192 | 5.17 | | 3.63 | 0.04 | 2.91 | -0.31 | 0.47 | -0.16 |
|  | 16 | SWU10062 | SWU10094 | | 20 | | **SWU20246** | **SWU20501a** | 7.42 | | 5.47 | 0.02 | 3.58 | 0.07 | -0.27 | 0.20 |
|  | 20 | SWU20649 | SWU20636 | | 20 | | SWU1259 | SWU20033 | 6.42 | | 4.41 | 0.35 | -3.21 | -1.08 | -0.06 | 1.15 |
|  | 11 | ICR01787 | NAU3695 | | 21 | | SWU16487 | SWU16488 | 5.39 | | 3.19 | 0.39 | 2.72 | 1.38 | -0.71 | -0.67 |
|  | 14 | SWU14224 | DPL0565 | | 21 | | SWU16488 | SWU16138 | 5.59 | | 3.71 | 0.24 | -2.94 | 0.85 | -1.01 | 0.16 |
|  | 20 | SWU20035 | DPL0319 | | 21 | | CGR5602 | JESPR154 | 6.35 | | 4.42 | 0.17 | 3.25 | -0.03 | 0.77 | -0.74 |
|  | 5 | DPL0022 | SWU17787 | | 21 | | CGR5808 | HAU0423 | 5.62 | | 3.95 | 0.03 | -3.02 | 0.10 | 0.13 | -0.22 |
|  | 14 | SWU14224 | DPL0565 | | 22 | | DPL0562 | **CAU0161** | 5.02 | | 3.44 | 0.17 | 2.96 | 0.18 | 0.71 | -0.89 |
|  | 9 | Gh27 | SWU15194 | | 24 | | SWU13267 | BNL1521 | 5.42 | | 3.28 | 0.55 | 2.73 | 0.43 | 1.11 | -1.54 |
|  | 11 | ICR01787 | NAU3695 | | 24 | | SWU13758 | CGR5423 | 5.05 | | 2.49 | 0.89 | 2.38 | 0.91 | 1.11 | -2.01 |
|  | 4 | **SWU12672** | HAU1332 | | 25 | | Gh220 | SWU19434 | 5.50 | | 3.74 | 0.18 | -2.93 | 0.70 | -0.84 | 0.14 |
|  | 13 | BNL1495 | CGR5390 | | 26 | | SWU17336 | NAU5072 | 8.62 | | 5.66 | 0.22 | -3.65 | 0.19 | -0.97 | 0.79 |
|  | 25 | Gh220 | SWU19434 | | 26 | | SWU17336 | NAU5072 | 6.18 | | 4.23 | 0.16 | 3.11 | -0.32 | -0.53 | 0.85 |
|  | 5 | PGML1671 | PGML1917 | | 27 | | SWU11038 | SWU11384 | 5.55 | | 3.77 | 0.02 | -2.94 | -0.15 | 0.43 | -0.29 |
| LY | 1 | SWU10986 | NAU2218 | | 2 | | **SWU11887** | SWU11976 | 6.10 | | 4.01 | 0.13 | -1.21 | 0.10 | 0.20 | -0.30 |
|  | 6 | ICR03206 | NAU896 | | 7 | | Gh474 | SWU10785 | 7.37 | | 4.35 | 0.52 | -1.28 | -0.06 | -0.50 | 0.56 |
|  | 6 | NAU896 | BNL3650 | | 9 | | SWU15157 | SWU14934 | 5.74 | | 3.88 | 0.25 | -1.18 | -0.01 | -0.36 | 0.36 |
|  | 9 | NAU3966 | SWU15157 | | 10 | | CGR5873 | ICR00093 | 5.33 | | 3.48 | 0.11 | 1.13 | 0.28 | -0.19 | -0.08 |
|  | 5 | SWU17787 | SWU13378 | | 11 | | NAU1014 | ICR10344 | 5.83 | | 3.71 | 0.35 | 1.17 | -0.46 | 0.41 | 0.05 |
|  | 10 | NAU2139 | SWU20689 | | 12 | | HAU1316 | NAU3519 | 6.50 | | 4.01 | 0.51 | 1.21 | -0.51 | 0.55 | -0.04 |
|  | 10 | HAU0635 | NAU2139 | | 13 | | BNL1495 | CGR5390 | 5.67 | | 2.98 | 0.72 | 1.04 | -0.47 | 0.71 | -0.24 |
|  | 13 | SWU22309 | SWU22324 | | 13 | | DPL0894 | SWU10800 | 5.48 | | 3.41 | 0.57 | -1.19 | 0.43 | 0.30 | -0.73 |
|  | 5 | DPL0022 | SWU17787 | | 16 | | NAU862 | CGR6802 | 5.58 | | 2.93 | 1.10 | 1.03 | -0.88 | 0.29 | 0.58 |
|  | 12 | DPL0303 | COT107 | | 17 | | NAU3765 | SWU14627 | 6.04 | | 3.54 | 0.60 | -1.14 | 0.35 | 0.32 | -0.66 |
|  | 16 | SWU10062 | SWU10094 | | 20 | | **SWU20246** | **SWU20501a** | 7.16 | | 5.10 | 0.10 | 1.37 | -0.04 | -0.22 | 0.25 |
|  | 11 | ICR01787 | NAU3695 | | 21 | | SWU16487 | SWU16488 | 5.49 | | 3.35 | 0.30 | 1.11 | 0.47 | -0.33 | -0.13 |
|  | 14 | SWU14224 | DPL0565 | | 21 | | SWU16488 | SWU16138 | 5.41 | | 3.80 | 0.03 | -1.18 | 0.10 | -0.17 | 0.07 |
|  | 15 | DPL0182 | SWU11691 | | 21 | | SWU16488 | SWU16138 | 5.14 | | 3.62 | 0.00 | 1.15 | -0.02 | 0.02 | 0.00 |
|  | 20 | SWU20035 | DPL0319 | | 21 | | SWU14431a | SWU15915 | 8.09 | | 5.90 | 0.02 | 1.48 | 0.02 | 0.12 | -0.14 |
|  | 4 | BNL1167 | SWU21415 | | 25 | | Gh220 | SWU19434 | 5.25 | | 2.93 | 0.76 | -1.03 | 0.66 | -0.62 | -0.03 |
|  | 13 | BNL1495 | CGR5390 | | 26 | | SWU17336 | NAU5072 | 5.27 | | 3.28 | 0.24 | -1.10 | 0.16 | -0.42 | 0.26 |
|  | 25 | Gh220 | SWU19434 | | 26 | | SWU17336 | NAU5072 | 6.43 | | 4.02 | 0.41 | 1.20 | -0.22 | -0.32 | 0.54 |
|  | 14 | CIR228 | BNL2485 | | 26 | | BNL2495 | DPL0491 | 5.13 | | 3.60 | 0.06 | 1.14 | -0.16 | 0.24 | -0.07 |
|  | 9 | PGML2830 | CGR6876 | | 26 | | SWU18681 | SWU0598 | 5.01 | | 3.51 | 0.05 | -1.12 | 0.11 | -0.16 | 0.05 |
|  | 5 | PGML1671 | PGML1917 | | 27 | | SWU11038 | SWU11384 | 5.61 | | 3.80 | 0.12 | -1.17 | 0.08 | 0.21 | -0.29 |
|  | 28 | **TMB2386** | SWU12343 | | 29 | | BNL3261 | CGR5111 | 5.17 | | 3.35 | 0.16 | 1.10 | -0.29 | 0.28 | 0.01 |
|  | 26 | SWU17251 | C2_0135 | | 29 | | C2_0115 | ICR03107 | 5.45 | | 2.89 | 0.73 | -1.02 | 0.59 | 0.07 | -0.66 |
|  | 10 | ICR00093 | ICR07050 | | 30 | | BNL243 | CER0168 | 5.78 | | 3.94 | 0.15 | -1.20 | 0.30 | -0.29 | -0.01 |
| BNP | 6 | HAU1460 | HAU1371 | | 7 | | SWU10064 | NAU3181 | 6.08 | | 2.07 | 1.79 | -0.45 | 0.26 | -0.59 | 0.33 |
|  | 2 | SWU11887 | SWU11976 | | 8 | | HAU1470a | SHIN1341 | 5.25 | | 1.55 | 2.12 | 0.39 | -0.40 | 0.63 | -0.23 |
|  | 5 | SWU17787 | SWU13378 | | 11 | | NAU1014 | ICR10344 | 7.54 | | 2.92 | 1.75 | 0.54 | -0.24 | 0.59 | -0.35 |
|  | 21 | CGR5808 | HAU0423 | | 21 | | CGR5806 | DPL0777 | 5.10 | | 2.35 | 1.26 | 0.55 | -0.39 | 0.51 | -0.11 |
|  | 24 | Gh298 | SWU13133 | | 24 | | HAU2504 | SWU13736 | 5.48 | | 2.72 | 1.03 | 0.52 | -0.29 | 0.44 | -0.16 |
|  | 2 | SWU11950 | TMB1268 | | 25 | | CGR6864 | SWU19815 | 5.27 | | 1.77 | 2.24 | -0.42 | 0.35 | -0.66 | 0.31 |
|  | 5 | DPL0022 | SWU17787 | | 26 | | SWU17336 | NAU5072 | 5.04 | | 1.43 | 1.62 | 0.38 | -0.05 | 0.54 | -0.49 |
|  | 13 | NAU2893 | Gh157 | | 26 | | DPL0491 | Gh64 | 5.37 | | 3.03 | 0.85 | -0.55 | 0.13 | -0.40 | 0.27 |
|  | 9 | CGR6876 | CGR5758 | | 26 | | SWU18488 | SWU18672 | 6.50 | | 2.44 | 2.17 | -0.49 | 0.29 | -0.65 | 0.37 |
|  | 14 | NAU2960 | ICR12130 | | 30 | | CER0168 | SWU21718 | 5.03 | | 1.07 | 2.94 | -0.32 | 0.51 | -0.74 | 0.23 |
| BW | 1 | NAU6367 | MUSS422 | | 2 | | SWU12025 | SWU11889 | 6.76 | | 4.77 | 0.19 | 0.10 | -0.02 | 0.02 | 0.00 |
|  | 1 | NAU3384 | CGR5663 | | 4 | | SWU18881 | NAU2701 | 5.07 | | 3.59 | 0.03 | 0.09 | -0.01 | 0.01 | 0.00 |
|  | 5 | SWU20913 | Gh260 | | 10 | | Gh320 | HAU0635 | 5.48 | | 3.96 | 0.08 | 0.09 | 0.02 | -0.01 | -0.01 |
|  | 1 | BNL2827a | NAU6367 | | 10 | | NAU2139 | SWU20689 | 5.22 | | 2.98 | 0.80 | 0.08 | -0.05 | 0.05 | -0.01 |
|  | 13 | SWU22374 | HAU2857 | | 15 | | CGR6889 | DPL0182 | 5.04 | | 3.18 | 0.35 | -0.09 | 0.02 | -0.04 | 0.02 |
|  | 11 | PGML2202 | CGR6580 | | 17 | | NAU3765 | SWU14627 | 6.02 | | 4.05 | 0.43 | 0.09 | -0.04 | 0.04 | 0.01 |
|  | 10 | ICR00093 | ICR07050 | | 18 | | Gh60 | SWU22281 | 6.55 | | 4.80 | 0.00 | -0.10 | 0.00 | 0.00 | 0.00 |
|  | 13 | SWU22413 | CGR5331 | | 18 | | CIR099 | NAU748 | 5.74 | | 3.76 | 0.10 | 0.09 | 0.01 | 0.01 | -0.02 |
|  | 16 | **SWU10038** | **ICR00016** | | 19 | | **NAU3437** | **NAU2894** | 5.55 | | 3.35 | 0.31 | 0.09 | 0.04 | -0.01 | -0.02 |
|  | 7 | NAU3181 | SHIN0376 | | 19 | | SWU14431b | SWU17782 | 5.05 | | 3.31 | 0.19 | -0.08 | 0.00 | -0.02 | 0.03 |
|  | 2 | SWU12025 | SWU11889 | | 20 | | SWU20700 | CGR5548 | 6.46 | | 4.48 | 0.26 | 0.10 | 0.03 | -0.01 | -0.02 |
|  | 10 | SWU20501b | CGR5873 | | 20 | | SWU20636 | **CGR6154** | 5.07 | | 3.51 | 0.21 | -0.09 | -0.03 | 0.00 | 0.03 |
|  | 16 | SWU10062 | **SWU10094** | | 20 | | **SWU20501a** | **CER0167** | 5.57 | | 4.02 | 0.06 | 0.09 | -0.01 | 0.02 | -0.01 |
|  | 20 | SWU20035 | DPL0319 | | 21 | | SWU16573 | Gh451 | 7.15 | | 5.07 | 0.08 | -0.10 | -0.02 | 0.01 | 0.01 |
|  | 12 | HAU1316 | NAU3519 | | 21 | | Gh451 | SWU16489 | 5.94 | | 3.99 | 0.21 | -0.09 | 0.00 | -0.02 | 0.03 |
|  | 1 | SWU10912 | DPL0090 | | 21 | | HAU0423 | CGR5806 | 5.10 | | 3.61 | 0.01 | 0.09 | 0.01 | 0.00 | -0.01 |
|  | 1 | NAU6367 | MUSS422 | | 22 | | PGML1712 | SWU21538 | 6.65 | | 4.77 | 0.08 | -0.10 | -0.02 | -0.01 | 0.02 |
|  | 5 | **SWU20917** | **NAU6240** | | 22 | | SWU21533 | DPL0562 | 5.31 | | 3.62 | 0.31 | -0.09 | 0.03 | -0.03 | 0.01 |
|  | 9 | NAU3966 | SWU15157 | | 24 | | CGR5202 | Gh298 | 5.54 | | 3.94 | 0.17 | 0.09 | 0.01 | -0.03 | 0.02 |
|  | 14 | PGML4763 | SWU13909 | | 24 | | BNL1521 | HAU2504 | 6.94 | | 4.51 | 0.54 | 0.10 | 0.00 | 0.04 | -0.04 |
|  | 21 | SWU0830 | HAU2004 | | 26 | | MGHES31 | HAU1571 | 6.40 | | 4.60 | 0.10 | 0.10 | 0.01 | 0.01 | -0.02 |
|  | 24 | SWU13758 | CGR5423 | | 26 | | MGHES31 | HAU1571 | 6.37 | | 4.12 | 0.10 | 0.09 | 0.00 | 0.02 | -0.02 |
|  | 21 | CGR5217 | BNL3442a | | 27 | | SWU11038 | SWU11384 | 5.89 | | 3.81 | 0.35 | -0.09 | 0.02 | -0.04 | 0.02 |
|  | 21 | CGR5806 | DPL0777 | | 28 | | SHIN0219 | TMB2386 | 6.72 | | 4.50 | 0.24 | -0.10 | 0.03 | 0.00 | -0.03 |
|  | 15 | DC40183 | DC40175 | | 29 | | C2_0115 | ICR03107 | 5.79 | | 3.69 | 0.40 | -0.09 | 0.02 | -0.04 | 0.02 |
|  | 1 | SWU11191 | BNL2827b | | 30 | | BNL243 | CER0168 | 5.40 | | 2.98 | 0.77 | 0.08 | 0.05 | 0.01 | -0.05 |
| LP | 5 | **TMB1296** | **HAU1603** | | 7 | | CER0036 | PGML1916 | 5.62 | | 2.88 | 0.18 | -0.41 | 0.04 | -0.14 | 0.10 |
|  | 4 | SWU18881 | NAU2701 | | 11 | | **ICR01810** | CGR6525 | 5.91 | | 3.13 | 0.03 | -0.42 | 0.05 | -0.06 | 0.01 |
|  | 9 | Gh27 | SWU15194 | | 11 | | CAU0003 | DC40250 | 5.35 | | 2.40 | 0.49 | 0.37 | 0.01 | 0.20 | -0.21 |
|  | 1 | ICR03724 | ICR03725 | | 13 | | DPL0535 | CER0165 | 5.54 | | 3.12 | 0.08 | 0.43 | 0.08 | -0.06 | -0.02 |
|  | 11 | NAU2152 | NAU5428 | | 14 | | TMB0071 | BNL3661 | 7.02 | | 3.33 | 0.26 | -0.45 | -0.05 | -0.12 | 0.17 |
|  | 1 | ICR03725 | SWU10987 | | 14 | | **CIR228** | **BNL2485** | 6.13 | | 3.46 | 0.00 | -0.45 | -0.01 | 0.02 | -0.01 |
|  | 4 | SWU16783 | NAU3868 | | 16 | | CGR6802 | HAU1129 | 5.23 | | 2.95 | 0.08 | -0.41 | 0.09 | -0.09 | 0.01 |
|  | 8 | HAU1470a | SHIN1341 | | 16 | | HAU1129 | NAU2984 | 5.02 | | 2.63 | 0.07 | 0.40 | 0.01 | 0.07 | -0.08 |
|  | 14 | ICR12037 | CGR5675 | | 16 | | SWU10062 | SWU10094 | 5.98 | | 3.18 | 0.12 | 0.43 | -0.05 | 0.12 | -0.07 |
|  | 16 | ICR00010 | SWU10038 | | 17 | | NAU3765 | SWU14627 | 8.27 | | 4.31 | 0.05 | -0.51 | -0.06 | -0.01 | 0.07 |
|  | 11 | CER0098 | CGR5421 | | 17 | | NAU3765 | SWU14627 | 5.75 | | 3.19 | 0.08 | -0.45 | -0.09 | 0.08 | 0.01 |
|  | 3 | SWU12819 | SWU12765 | | 20 | | SWU20501a | **CER0167** | 5.57 | | 2.72 | 0.09 | 0.41 | 0.03 | -0.10 | 0.07 |
|  | 13 | DPL0572 | HAU2558 | | 20 | | SWU20501a | **CER0167** | 5.34 | | 2.70 | 0.22 | -0.39 | -0.12 | -0.03 | 0.14 |
|  | 11 | NAU3390 | NAU2460 | | 20 | | SWU20501a | **CER0167** | 5.78 | | 2.70 | 0.20 | 0.39 | -0.03 | 0.14 | -0.11 |
|  | 21 | Gh451 | SWU16489 | | 21 | | SWU16488 | SWU16138 | 7.82 | | 3.78 | 0.11 | -0.51 | -0.15 | 0.06 | 0.09 |
|  | 16 | NAU862 | CGR6802 | | 21 | | CGR5806 | DPL0777 | 5.11 | | 3.02 | 0.00 | 0.42 | -0.03 | 0.02 | 0.01 |
|  | 4 | SWU21617 | SWU11855 | | 25 | | HAU1382 | SWU19848 | 5.83 | | 2.95 | 0.21 | -0.42 | 0.10 | -0.15 | 0.05 |
|  | 18 | NAU748 | SWU22192 | | 25 | | SWU19848 | CGR6864 | 6.12 | | 2.96 | 0.23 | 0.41 | -0.11 | -0.05 | 0.16 |
|  | 12 | HAU1316 | NAU3519 | | 25 | | DPL0282 | SWU19763 | 7.61 | | 4.10 | 0.12 | 0.49 | -0.12 | 0.10 | 0.02 |
|  | 25 | DPL0282 | SWU19763 | | 25 | | Gh220 | SWU19434 | 6.25 | | 3.42 | 0.04 | 0.45 | -0.07 | 0.02 | 0.04 |
|  | 21 | SWU16138 | BNL1053 | | 26 | | MGHES31 | HAU1571 | 10.04 | | 5.55 | 0.06 | -0.57 | 0.07 | -0.08 | 0.01 |
|  | 22 | **DPL0562** | **CAU0161** | | 26 | | SWU17233 | SWU17251 | 6.08 | | 2.65 | 0.40 | 0.41 | 0.01 | 0.18 | -0.19 |
|  | 16 | Gh56 | NAU5120 | | 26 | | SWU0514 | SWU18488 | 6.97 | | 3.72 | 0.16 | -0.46 | 0.02 | -0.13 | 0.10 |
|  | 26 | C2_0135 | PGML2321 | | 26 | | SWU18488 | **SWU18672** | 5.27 | | 2.50 | 0.04 | -0.42 | 0.05 | -0.05 | 0.00 |
|  | 13 | DPL0572 | HAU2558 | | 27 | | SWU10994 | HAU1001 | 5.06 | | 2.45 | 0.16 | -0.40 | 0.12 | -0.11 | -0.02 |
|  | 1 | CGR5663 | NAU2343 | | 27 | | SWU11384 | ICR11885 | 5.63 | | 3.05 | 0.02 | -0.42 | 0.06 | -0.05 | -0.01 |
|  | 17 | NAU3765 | SWU14627 | | 28 | | SWU12343 | SWU14060 | 8.88 | | 4.47 | 0.13 | -0.53 | -0.02 | -0.09 | 0.11 |
|  | 14 | TMB0071 | BNL3661 | | 29 | | BNL3261 | CGR5111 | 5.12 | | 2.79 | 0.05 | 0.40 | 0.08 | -0.04 | -0.04 |
|  | 16 | SWU10266 | DC40065 | | 29 | | C2_0115 | ICR03107 | 6.63 | | 3.04 | 0.34 | 0.42 | 0.06 | 0.13 | -0.20 |
|  | 4 | ICR01729 | SWU16781 | | 30 | | CER0168 | SWU21718 | 5.82 | | 3.27 | 0.01 | -0.43 | 0.03 | 0.00 | -0.03 |
|  | 11 | CAU0003 | DC40250 | | 30 | | CER0168 | SWU21718 | 6.70 | | 3.24 | 0.28 | 0.43 | -0.18 | 0.12 | 0.06 |

**Supplementary Table S7-C. Main effect QTLs and environmental interactions detected for yield and yield-component traits in BC/P populations.** See footnotes in Table S7-A.

| **Trait** | **Chr** | **Left marker** | **Right marker** | **LOD** | **V(A)%** | **V(AE)%** | **Effect value** | | | |
| --- | --- | --- | --- | --- | --- | --- | --- | --- | --- | --- |
|  |  |  |  |  |  |  | **A** | **AE1** | **AE2** | **AE3** |
| SY | 2 | SWU11889 | SWU11887 | 4.35 | 3.48 | 0.01 | 2.25 | -0.04 | -0.09 | 0.13 |
|  | 2 | SWU11976 | SWU11950 | 7.41 | 4.72 | 1.18 | 2.62 | -0.92 | 1.85 | -0.93 |
|  | 4 | BNL530 | SWU21485 | 2.97 | 2.33 | 0.09 | 1.85 | -0.18 | -0.32 | 0.50 |
|  | 16 | NAU2984 | SWU10062 | 2.74 | 0.63 | 1.57 | 0.97 | -0.02 | -1.86 | 1.88 |
|  | 20 | SWU20246 | SWU20501a | 2.85 | 2.02 | 0.14 | -1.72 | -0.45 | -0.16 | 0.61 |
|  | 22 | CAU0161 | NAU2026 | 3.60 | 2.86 | 0.04 | -2.20 | 0.37 | -0.28 | -0.09 |
|  | 28 | ICR11064 | NAU935 | 3.06 | 2.03 | 0.51 | 1.74 | 0.78 | -1.22 | 0.44 |
| LY | 2 | SWU11889 | SWU11887 | 5.28 | 4.18 | 0.01 | 1.00 | -0.01 | -0.05 | 0.06 |
|  | 2 | SWU11976 | SWU11950 | 7.01 | 4.54 | 1.10 | 1.04 | -0.33 | 0.72 | -0.39 |
|  | 4 | BNL530 | SWU21485 | 3.00 | 2.40 | 0.12 | 0.75 | -0.14 | -0.10 | 0.24 |
|  | 7 | NAU3181 | SHIN0376 | 3.15 | 2.34 | 0.26 | -0.75 | 0.35 | -0.17 | -0.18 |
|  | 22 | CAU0161 | NAU2026 | 3.02 | 2.45 | 0.04 | -0.82 | 0.15 | -0.06 | -0.09 |
|  | 25 | SWU19430 | PGML1219 | 2.92 | 2.14 | 0.23 | -0.71 | -0.04 | 0.31 | -0.26 |
|  | 28 | ICR11064 | NAU935 | 3.34 | 2.22 | 0.48 | 0.73 | 0.33 | -0.47 | 0.15 |
| BNP | 1 | NAU3177 | ICR03724 | 3.09 | 2.25 | 0.37 | -0.38 | 0.21 | -0.15 | -0.06 |
|  | 1 | SWU11191 | BNL2827b | 2.89 | 1.64 | 0.89 | -0.33 | 0.23 | -0.33 | 0.10 |
|  | 28 | ICR11064 | NAU935 | 2.86 | 1.68 | 0.20 | 0.33 | 0.09 | -0.16 | 0.07 |
| BW | 2 | SWU11887 | SWU11976 | 2.67 | 1.61 | 0.13 | 0.05 | -0.01 | -0.01 | 0.02 |
|  | 5 | SWU20913 | Gh260 | 5.04 | 2.48 | 2.01 | 0.06 | -0.05 | 0.07 | -0.02 |
|  | 5 | **NAU6240** | PGML1671 | 3.87 | 2.16 | 0.32 | 0.05 | -0.01 | -0.01 | 0.03 |
|  | 20 | SWU20501a | CER0167 | 4.55 | 3.15 | 0.54 | -0.06 | 0.03 | -0.03 | 0.00 |
| LP | 5 | SWU20917 | NAU6240 | 18.77 | 13.26 | 0.26 | -0.59 | 0.08 | -0.11 | 0.03 |
|  | 5 | TMB1296 | **HAU1603** | 3.66 | 2.31 | 0.11 | -0.25 | -0.06 | 0.00 | 0.07 |
|  | 6 | NAU896 | BNL3650 | 3.15 | 1.44 | 0.40 | -0.19 | 0.14 | -0.06 | -0.08 |
|  | 6 | ICR10602 | SWU19656 | 3.16 | 1.93 | 0.10 | -0.22 | 0.07 | -0.03 | -0.04 |
|  | 7 | SWU10064 | NAU3181 | 2.51 | 1.63 | 0.02 | -0.21 | 0.03 | -0.03 | 0.01 |
|  | 12 | **HAU1316** | **NAU3519** | 2.74 | 1.13 | 0.45 | -0.17 | 0.06 | 0.09 | -0.15 |
|  | 13 | DPL0308 | DPL0535 | 2.72 | 1.82 | 0.04 | -0.22 | -0.01 | -0.03 | 0.05 |
|  | 13 | **NAU2893** | Gh157 | 3.18 | 1.95 | 0.36 | -0.23 | -0.01 | -0.12 | 0.12 |
|  | 13 | BNL1495 | CGR5390 | 8.11 | 4.94 | 0.26 | -0.36 | 0.08 | 0.03 | -0.11 |
|  | 14 | NAU3820 | NAU2960 | 2.68 | 1.68 | 0.08 | 0.21 | 0.03 | 0.03 | -0.06 |
|  | 19 | NAU3437 | NAU2894 | 3.78 | 2.31 | 0.25 | 0.25 | 0.11 | -0.03 | -0.08 |
|  | 22 | PGML0695 | SWU20813 | 2.75 | 1.23 | 0.41 | 0.18 | 0.09 | -0.14 | 0.05 |
|  | 27 | CGR5056 | DC40052 | 3.13 | 1.52 | 0.35 | -0.20 | -0.09 | 0.14 | -0.05 |

**Supplementary Table S7-D. Epistatic QTLs and environmental interactions detected for yield and yield-component traits in BC/P populations.** See footnotes in Table S7-B.

| **Trait** | **Ch*i*** | **Left marker** | **Right marker** | | **Ch*j*** | **Left marker** | **Right marker** | | **LOD** | **V(AA)%** | **V(AAE)%** | **Effect value** | | | |
| --- | --- | --- | --- | --- | --- | --- | --- | --- | --- | --- | --- | --- | --- | --- | --- |
|  |  |  |  | |  |  |  | |  |  |  | **AA** | **AAE1** | **AAE2** | **AAE3** |
| SY | 1 | NAU3384 | CGR5663 | 5 | | SWU20913 | Gh260 | 5.34 | | 1.38 | 2.28 | -1.41 | -0.31 | 2.37 | -2.07 |
|  | 10 | ICR00093 | ICR07050 | 11 | | NAU1014 | ICR10344 | 5.35 | | 2.75 | 1.11 | 2.02 | 0.17 | -1.65 | 1.48 |
|  | 13 | HAU2558 | NAU2893 | 13 | | BNL1495 | CGR5390 | 6.87 | | 3.92 | 0.00 | -3.22 | 0.09 | -1.63 | 1.53 |
|  | 5 | SWU20913 | Gh260 | 14 | | PGML4763 | SWU13909 | 5.12 | | 2.84 | 0.59 | 2.04 | 1.07 | -1.20 | 0.13 |
|  | 1 | HAU1417 | NAU2437 | 21 | | SWU16649 | BNL1552 | 6.50 | | 3.90 | 0.66 | 2.40 | 0.22 | 1.07 | -1.29 |
|  | 16 | SWU10038 | ICR00016 | 26 | | C2_0135 | PGML2321 | 5.40 | | 3.71 | 0.49 | 2.45 | -1.10 | 0.22 | 0.89 |
| LY | 1 | CGR5663 | NAU2343 | 5 | | SWU20913 | Gh260 | 5.30 | | 1.41 | 2.17 | -0.58 | -0.21 | 0.97 | -0.76 |
|  | 13 | HAU2558 | NAU2893 | 13 | | BNL1495 | CGR5390 | 6.86 | | 3.10 | 0.00 | -1.24 | 0.09 | -0.75 | 0.66 |
|  | 13 | DPL0894 | SWU10800 | 17 | | SWU12818 | CGR5576 | 5.35 | | 3.10 | 0.65 | -0.89 | 0.24 | -0.56 | 0.32 |
|  | 1 | HAU1417 | NAU2437 | 21 | | SWU16573 | Gh451 | 5.91 | | 3.38 | 0.60 | 0.92 | 0.11 | 0.40 | -0.51 |
|  | 6 | NAU896 | BNL3650 | 24 | | PGML4657 | Gh454 | 5.63 | | 3.00 | 0.52 | 0.85 | 0.32 | -0.49 | 0.17 |
|  | 16 | SWU10038 | ICR00016 | 26 | | C2_0135 | PGML2321 | 5.57 | | 3.77 | 0.41 | 1.00 | -0.38 | 0.02 | 0.37 |
| BNP | 5 | HAU1603 | PGML4457 | 14 | | NAU2960 | ICR12130 | 5.09 | | 2.75 | 0.76 | -0.42 | 0.31 | -0.17 | -0.14 |
|  | 3 | CER0028 | Gh663 | 15 | | DPL0182 | SWU11691 | 5.01 | | 1.53 | 1.30 | 0.31 | -0.40 | 0.11 | 0.29 |
|  | 13 | SWU22309 | SWU22324 | 16 | | SWU10214 | Gh56 | 5.55 | | 1.38 | 2.04 | 0.33 | -0.52 | 0.38 | 0.14 |
|  | 13 | BNL1495 | CGR5390 | 17 | | SWU12818 | CGR5576 | 5.22 | | 2.24 | 1.13 | -0.40 | 0.36 | -0.35 | -0.01 |
|  | 8 | HAU3177 | NAU4064 | 17 | | SWU12818 | CGR5576 | 7.09 | | 1.79 | 2.71 | -0.35 | 0.60 | -0.25 | -0.35 |
|  | 14 | CIR228 | BNL2485 | 18 | | SWU22287 | SWU22290 | 5.21 | | 2.50 | 1.11 | 0.40 | -0.38 | 0.16 | 0.22 |
|  | 16 | Gh56 | NAU5120 | 21 | | CGR5808 | HAU0423 | 6.51 | | 3.54 | 0.86 | 0.48 | -0.31 | 0.23 | 0.08 |
|  | 20 | SWU20636 | CGR6154 | 25 | | SWU19815 | BNL3594 | 5.49 | | 0.94 | 2.45 | 0.25 | -0.57 | 0.33 | 0.23 |
|  | 13 | BNL1495 | CGR5390 | 25 | | SWU19411 | SWU19412 | 6.19 | | 4.12 | 0.33 | -0.52 | 0.19 | -0.11 | -0.07 |
|  | 16 | SWU10038 | ICR00016 | 26 | | SWU17251 | C2_0135 | 5.77 | | 2.99 | 0.85 | 0.46 | -0.26 | 0.33 | -0.07 |
|  | 12 | NAU943 | DPL0303 | 27 | | SWU11384 | ICR11885 | 7.10 | | 3.37 | 1.38 | 0.46 | -0.41 | 0.13 | 0.28 |
|  | 13 | SWU22374 | HAU2857 | 27 | | CGR6857 | ICR11205 | 5.49 | | 1.32 | 1.83 | -0.30 | 0.46 | -0.39 | -0.07 |
|  | 20 | CER0167 | SWU20064 | 28 | | SHIN0219 | TMB2386 | 6.53 | | 2.03 | 1.87 | 0.36 | -0.46 | 0.38 | 0.08 |
|  | 17 | CGR5871 | SWU12876 | 29 | | DC20127 | DPL0252 | 5.54 | | 2.99 | 0.77 | -0.44 | 0.25 | -0.29 | 0.04 |
| BW | 1 | SWU0077 | HAU1417 | 6 | | HAU1460 | HAU1371 | 5.67 | | 3.82 | 0.02 | -0.07 | 0.00 | 0.00 | 0.00 |
|  | 5 | SWU13378 | SWU17846 | 11 | | ICR01810 | CGR6525 | 8.36 | | 5.87 | 0.02 | -0.09 | 0.00 | 0.00 | 0.01 |
|  | 14 | PGML4763 | SWU13909 | 14 | | ICR12037 | CGR5675 | 5.00 | | 3.46 | 0.19 | 0.07 | 0.01 | 0.01 | -0.02 |
|  | 5 | SWU20917 | **NAU6240** | 16 | | DPL0048 | SWU10266 | 5.57 | | 3.73 | 0.00 | -0.07 | -0.01 | 0.01 | 0.00 |
|  | 10 | ICR00093 | ICR07050 | 18 | | DC40150 | ICR02849 | 5.45 | | 1.96 | 1.86 | -0.05 | 0.04 | -0.07 | 0.04 |
|  | 13 | SHIN1462 | SWU22374 | 18 | | DC40150 | ICR02849 | 5.61 | | 2.08 | 0.24 | 0.08 | 0.01 | 0.02 | -0.03 |
|  | 7 | CGR5001 | CGR6586 | 26 | | SWU17432 | SWU17395 | 5.56 | | 3.53 | 0.43 | 0.07 | 0.03 | -0.03 | 0.00 |
|  | 21 | CGR5806 | DPL0777 | 27 | | SWU11038 | SWU11384 | 5.03 | | 2.60 | 0.53 | -0.06 | 0.03 | -0.03 | -0.01 |
|  | 4 | SWU12672 | HAU1332 | 27 | | SWU11384 | ICR11885 | 5.03 | | 2.03 | 1.49 | 0.05 | -0.04 | 0.06 | -0.02 |
|  | 14 | PGML1568 | Gh529 | 28 | | CGR5534 | SHIN0219 | 6.65 | | 3.82 | 0.77 | 0.07 | -0.03 | 0.04 | -0.01 |
| LP | 5 | SWU13378 | SWU17846 | 11 | | ICR01810 | CGR6525 | 5.85 | | 3.58 | 0.02 | 0.31 | -0.02 | 0.03 | -0.01 |
|  | 13 | SWU22309 | SWU22324 | 13 | | HAU2558 | **NAU2893** | 5.56 | | 2.29 | 0.00 | -0.29 | -0.13 | -0.02 | 0.14 |
|  | 5 | **HAU1603** | PGML4457 | 14 | | TMB0071 | BNL3661 | 5.24 | | 2.71 | 0.20 | 0.27 | -0.10 | 0.03 | 0.07 |
|  | 1 | SWU10986 | NAU2218 | 14 | | CIR228 | BNL2485 | 5.73 | | 3.32 | 0.08 | -0.30 | -0.04 | 0.01 | 0.04 |
|  | 7 | PGML1916 | SWU10864 | 16 | | DPL0048 | SWU10266 | 5.69 | | 2.93 | 0.55 | 0.28 | -0.14 | 0.16 | -0.01 |
|  | 14 | ICR12037 | CGR5675 | 16 | | SWU10056 | SWU10037 | 5.74 | | 2.98 | 0.01 | 0.28 | 0.00 | 0.00 | 0.00 |
|  | 16 | SWU10056 | SWU10037 | 17 | | NAU3765 | SWU14627 | 6.22 | | 3.00 | 0.26 | -0.29 | -0.07 | 0.12 | -0.05 |
|  | 2 | SWU11950 | TMB1268 | 21 | | SWU16651 | SWU16645 | 5.01 | | 2.86 | 0.17 | -0.28 | 0.08 | -0.08 | 0.00 |
|  | 1 | SWU11191 | BNL2827b | 21 | | SWU16649 | BNL1552 | 6.26 | | 2.70 | 0.74 | 0.27 | -0.11 | 0.20 | -0.08 |
|  | 21 | Gh451 | SWU16489 | 21 | | SWU16488 | SWU16138 | 5.56 | | 2.45 | 0.29 | -0.28 | -0.13 | 0.03 | 0.10 |
|  | 20 | SWU20035 | DPL0319 | 21 | | SWU14431a | SWU15915 | 5.53 | | 3.28 | 0.00 | 0.30 | 0.00 | 0.01 | -0.01 |
|  | 10 | SWU20501b | CGR5873 | 21 | | CGR5806 | DPL0777 | 5.01 | | 2.67 | 0.11 | -0.28 | 0.00 | 0.07 | -0.06 |
|  | 12 | **HAU1316** | **NAU3519** | 25 | | DPL0282 | SWU19763 | 5.20 | | 2.58 | 0.32 | 0.26 | 0.09 | -0.12 | 0.03 |
|  | 10 | SWU20501b | CGR5873 | 25 | | SWU19411 | SWU19412 | 6.77 | | 4.00 | 0.01 | -0.33 | -0.01 | 0.01 | 0.00 |
|  | 16 | SWU10056 | SWU10037 | 26 | | NAU2175 | SWU17336 | 5.08 | | 2.55 | 0.16 | 0.26 | 0.06 | 0.02 | -0.08 |
|  | 22 | DPL0562 | CAU0161 | 26 | | Gh64 | SWU17257 | 6.34 | | 2.94 | 0.57 | 0.29 | 0.04 | 0.13 | -0.17 |
|  | 13 | SWU22413 | CGR5331 | 27 | | CGR6857 | ICR11205 | 5.29 | | 2.71 | 0.48 | -0.27 | 0.09 | -0.16 | 0.07 |
|  | 2 | SWU12126 | SWU12147 | 28 | | TMB2386 | SWU12343 | 5.03 | | 2.69 | 0.00 | 0.27 | 0.04 | -0.01 | -0.03 |
|  | 17 | NAU3765 | SWU14627 | 28 | | SWU12343 | SWU14060 | 6.28 | | 3.62 | 0.19 | -0.32 | -0.07 | 0.10 | -0.03 |
|  | 21 | SWU0830 | HAU2004 | 28 | | SWU12343 | SWU14060 | 5.21 | | 2.90 | 0.00 | 0.28 | 0.03 | -0.03 | 0.00 |
|  | 4 | ICR01729 | SWU16781 | 30 | | CER0168 | SWU21718 | 6.92 | | 3.71 | 0.25 | -0.31 | 0.02 | -0.10 | 0.08 |

**Supplementary Table S7-E. Main effect QTLs and environmental interactions detected for yield and yield-component traits in MPH-P datasets.** See footnotes in Table S7-A.

| **Trait** | **Chr** | **Left marker** | **Right marker** | **LOD** | **V(A)%** | **V(AE)%** | **Effect value** | | | |
| --- | --- | --- | --- | --- | --- | --- | --- | --- | --- | --- |
|  |  |  |  |  |  |  | **A** | **AE1** | **AE2** | **AE3** |
| SY | 2 | SWU11887 | SWU11976 | 4.9595 | 1.52 | 2.25 | 1.29 | -2.01 | 1.82 | 0.19 |
|  | 17 | ICR03391 | SWU12838a | 2.8396 | 1.46 | 0.80 | 1.29 | -1.34 | 0.77 | 0.57 |
|  | 17 | NAU3765 | **SWU14627** | 3.0034 | 1.34 | 0.88 | 1.23 | 0.92 | -1.39 | 0.46 |
|  | 26 | SWU18681 | SWU0598 | 2.5255 | 1.45 | 0.43 | -1.26 | 0.87 | -0.07 | -0.80 |
| LY | 2 | SWU11887 | SWU11976 | 4.9291 | 1.46 | 2.16 | 0.50 | -0.81 | 0.66 | 0.15 |
|  | 17 | ICR03391 | SWU12838a | 2.8053 | 1.27 | 1.02 | 0.48 | -0.60 | 0.37 | 0.23 |
|  | 17 | **NAU3765** | SWU14627 | 2.6215 | 1.56 | 0.37 | 0.53 | 0.23 | -0.36 | 0.13 |
| BNP | 1 | **NAU2218** | SWU11191 | 4.7661 | 2.34 | 3.11 | -0.39 | 0.35 | -0.63 | 0.28 |
|  | 5 | SWU13378 | SWU17846 | 3.5693 | 1.32 | 1.34 | -0.29 | 0.22 | 0.19 | -0.42 |
|  | 14 | CIR228 | BNL2485 | 2.7879 | 1.78 | 0.16 | -0.34 | 0.08 | 0.07 | -0.14 |
|  | 17 | **NAU3765** | SWU14627 | 3.3288 | 0.63 | 1.71 | 0.20 | -0.01 | -0.40 | 0.42 |
|  | 21 | BNL3171 | CGR5808 | 2.5917 | 0.75 | 1.87 | 0.22 | -0.13 | 0.47 | -0.34 |
| BW | 1 | HAU1417 | NAU2437 | 3.9734 | 1.68 | 1.41 | -0.05 | -0.05 | 0.00 | 0.05 |
|  | 5 | SWU20913 | Gh260 | 3.1875 | 0.74 | 2.37 | 0.03 | -0.07 | 0.06 | 0.00 |
|  | 13 | SWU22374 | HAU2857 | 2.5398 | 1.08 | 0.31 | 0.04 | 0.01 | -0.03 | 0.02 |
|  | 16 | DPL0897 | BNL1026 | 2.6301 | 0.16 | 1.43 | -0.01 | -0.01 | 0.05 | -0.05 |
|  | 20 | SWU20246 | SWU20501a | 2.531 | 0.68 | 2.16 | -0.03 | 0.06 | -0.07 | 0.01 |
| LP | 5 | NAU6240 | PGML1671 | 3.361 | 1.68 | 0.97 | -0.15 | 0.07 | -0.16 | 0.09 |
|  | 18 | SWU22187 | DC40150 | 4.0284 | 0.30 | 2.76 | 0.06 | -0.13 | 0.27 | -0.14 |
|  | 23 | Gh327 | SWU14770 | 2.625 | 1.77 | 0.54 | 0.15 | -0.06 | -0.05 | 0.12 |
|  | 27 | SWU11384 | ICR11885 | 3.0156 | 0.02 | 2.39 | -0.01 | -0.09 | 0.25 | -0.16 |
|  | 27 | CGR6356 | SWU11632 | 3.0894 | 0.00 | 2.48 | 0.01 | -0.05 | 0.24 | -0.19 |

**Supplementary Table S7-F. Epistatic QTLs and environmental interactions detected for yield and yield-component traits in MPH-P datasets.** See footnotes in Table S7-B

| **Trait** | **Ch*i*** | **Left marker** | **Right marker** | **Ch*j*** | **Left marker** | **Right marker** | **LOD** | **V(AA)%** | **V(AAE)%** | **Effect value** | | | |
| --- | --- | --- | --- | --- | --- | --- | --- | --- | --- | --- | --- | --- | --- |
|  |  |  |  |  |  |  |  |  |  | **AA** | **AAE1** | **AAE2** | **AAE3** |
| SY | 17 | **SWU14627** | CGR5871 | 19 | SWU17897 | CGR5539 | 5.27 | 3.01 | 0.94 | 1.85 | 1.34 | -1.02 | -0.32 |
|  | 13 | SWU22413 | CGR5331 | 27 | SWU11384 | ICR11885 | 6.33 | 4.02 | 0.35 | -2.11 | -0.77 | 0.02 | 0.75 |
| LY | 3 | CER0028 | Gh663 | 9 | NAU5474 | Gh158 | 5.52 | 2.45 | 1.02 | 0.65 | -0.12 | 0.57 | -0.44 |
|  | 6 | CGR5108 | ICR03206 | 26 | SWU17251 | C2_0135 | 5.58 | 3.28 | 0.31 | -0.76 | 0.23 | -0.32 | 0.09 |
|  | 26 | BNL2495 | DPL0491 | 27 | SWU10994 | HAU1001 | 5.60 | 3.82 | 0.37 | 0.81 | 0.33 | -0.29 | -0.04 |
|  | 13 | SWU22413 | CGR5331 | 27 | SWU11384 | ICR11885 | 5.83 | 3.93 | 0.25 | -0.83 | -0.24 | -0.03 | 0.27 |
|  | 17 | CGR5576 | **NAU3765** | 28 | BNL2877 | HAU3071 | 5.18 | 3.27 | 0.37 | 0.76 | 0.36 | -0.21 | -0.16 |
| BNP | 7 | PGML1916 | SWU10864 | 9 | Gh111 | Gh27 | 5.42 | 2.15 | 1.28 | 0.38 | -0.38 | 0.33 | 0.05 |
|  | 1 | HAU1417 | NAU2437 | 14 | SWU13909 | TMB0071 | 5.87 | 2.35 | 1.51 | -0.39 | 0.38 | -0.38 | -0.01 |
|  | 1 | NAU6367 | MUSS422 | 17 | CGR5576 | **NAU3765** | 6.07 | 1.12 | 2.86 | 0.28 | -0.59 | 0.44 | 0.16 |
|  | 13 | BNL1495 | CGR5390 | 25 | SWU19129 | PGML2858 | 5.73 | 2.32 | 1.30 | -0.40 | 0.43 | -0.28 | -0.15 |
|  | 19 | SWU17782 | DPL0056 | 26 | C2_0135 | PGML2321 | 5.22 | 0.87 | 2.31 | 0.24 | -0.49 | 0.45 | 0.03 |
|  | 1 | SWU10986 | **NAU2218** | 29 | C2_0115 | ICR03107 | 5.08 | 1.53 | 1.35 | -0.32 | 0.36 | 0.01 | -0.37 |
| BW | 4 | SWU16782 | SWU16783 | 5 | PGML1917 | SWU17715 | 5.46 | 2.62 | 1.51 | 0.06 | -0.02 | 0.06 | -0.04 |
|  | 11 | CGR6580 | SWU15972 | 14 | NAU3820 | NAU2960 | 6.23 | 3.34 | 1.75 | 0.06 | -0.03 | 0.07 | -0.03 |
|  | 3 | Gh663 | CGR6528 | 16 | SWU10627 | PGML1309 | 5.34 | 2.92 | 0.97 | -0.06 | 0.01 | -0.05 | 0.03 |
|  | 16 | SWU10060 | SWU10054 | 18 | DC40150 | ICR02849 | 5.39 | 2.01 | 2.31 | 0.05 | -0.02 | 0.08 | -0.05 |
|  | 2 | SWU11950 | TMB1268 | 26 | PGML1289 | SWU18919 | 5.40 | 2.99 | 1.14 | -0.06 | 0.02 | -0.05 | 0.03 |
|  | 14 | NAU3820 | NAU2960 | 28 | SHIN0219 | TMB2386 | 5.56 | 3.88 | 0.57 | 0.07 | -0.01 | 0.04 | -0.03 |
| LP | 3 | CER0028 | Gh663 | 20 | SWU20649 | SWU20636 | 5.46 | 0.68 | 3.00 | 0.10 | 0.08 | -0.28 | 0.19 |
|  | 5 | DPL0022 | SWU17787 | 25 | SWU19430 | PGML1219 | 5.27 | 0.63 | 2.61 | -0.09 | 0.22 | -0.23 | 0.01 |

**Supplementary Table S8. Genetic types of epistatic QTLs for yield and yield-component traits in BC/M and BC/P trials.** ^†^The BC/M trial was performed across 4 environments; ^§^The BC/P trial was performed across 3 environments. ^a^Three types of epistatic QTLs, Type I, interaction between two M-QTLs; Type II, interaction between one M-QTL and non M-QTL; Type III, interaction between two non M-QTLs. ^§^The number of QTL.

| **Trial** |  | **TypeⅠ^a^** |  |  | **Type Ⅱ** |  |  | **Type Ⅲ** |  | **n**^§^ |
| --- | --- | --- | --- | --- | --- | --- | --- | --- | --- | --- |
|  | **BC** | **MPH** | **RIL** | **BC** | **MPH** | **RIL** | **BC** | **MPH** | **RIL** |  |
| BC/M^†^ | 0 | 0 | 0 | 6 | 3 | 13 | 7 | 10 | 46 | 85 |
|  | 1 | 0 | 0 | 0 | 2 | 8 | 14 | 6 | 44 | 75 |
|  | 0 | 0 | 0 | 0 | 1 | 6 | 8 | 6 | 19 | 40 |
|  | 1 | 0 | 2 | 1 | 0 | 12 | 1 | 0 | 63 | 80 |
|  | 0 | 1 | 1 | 7 | 2 | 31 | 29 | 5 | 69 | 145 |
|  | 2 | 1 | 3 | 14 | 8 | 70 | 59 | 27 | 241 | 425 |
| BC/P^§^ | 0 | 0 | 0 | 0 | 1 | 4 | 6 | 1 | 20 | 32 |
|  | 0 | 0 | 0 | 0 | 1 | 3 | 6 | 4 | 21 | 35 |
|  | 0 | 0 | 0 | 0 | 2 | 0 | 14 | 4 | 11 | 31 |
|  | 0 | 0 | 2 | 1 | 0 | 2 | 9 | 6 | 22 | 42 |
|  | 0 | 0 | 0 | 3 | 0 | 8 | 18 | 2 | 23 | 54 |
|  | 0 | 0 | 2 | 4 | 4 | 17 | 53 | 17 | 97 | 194 |

**Supplementary Table S9. Common single locus QTLs detected in 2015 and 2016 and in previous 2012.** See footnotes in Table S2.

| **QTL** | **Env.** | **Flanking makers** | | **BC** | | |  | **MPH** | | |  | | **RIL** | | | ***d/a*** | **Type** |
| --- | --- | --- | --- | --- | --- | --- | --- | --- | --- | --- | --- | --- | --- | --- | --- | --- | --- |
|  |  |  |  | **LOD** | **Effect value** | **Var%** |  | **LOD** | **Effect value** | **Var%** |  | | **LOD** | **Effect value** | **Var%** |  |  |
| *qSY-Chr2-1* | 2015E2 | SWU11887 | SWU11976 |  |  |  |  |  |  |  |  | 3.23 | | 3.92 | 7.11 |  |  |
|  | 2015E2 | SWU11887 | SWU11976 |  |  |  |  |  |  |  |  | 2.89 | | 4.53 | 7.46 |  |  |
|  | 2016E1 | SWU11889 | SWU11887 | 8.88 | 6.46 | 27.26 |  | 4.46 | 4.00 | 12.41 |  |  | | (±2.46) |  | 1.6 | OD |
|  | 2016E2 | SWU11889 | SWU11887 |  |  |  |  | 3.10 | 2.61 | 7.01 |  |  | |  |  |  | OD |
|  | 2012E3 | SWU11887 | SWU11976 | 2.86 | 7.22 | 6.46 |  |  | (±1.50) |  |  | 8.38 | | 8.72 | 21.29 | 0.2 | PD |
| *qSY-Chr5-1* | 2016E2 | SWU20917 | NAU6240 | 3.63 | 3.28 | 9.41 |  |  |  |  |  |  | |  |  |  | A |
|  | 2012E2 | NAU6240 | PGML1671 |  |  |  |  |  |  |  |  | 4.57 | | 4.09 | 11.03 |  |  |
|  | 2012E3 | NAU6240 | PGML1671 | 3.69 | 8.59 | 9.46 |  |  | (±4.50) |  |  |  | |  |  | 1.1 | OD |
| *qSY-Chr21-1* | 2015E1 | CGR5808 | HAU0423 | 3.86 | 3.03 | 9.33 |  |  |  |  |  |  | |  |  |  | A |
|  | 2015E3 | CGR5808 | HAU0423 |  |  |  |  |  |  |  |  | 4.58 | | -4.19 | 9.20 |  |  |
|  | 2012E1 | CGR5808 | HAU0423 |  |  |  |  |  |  |  |  | 4.71 | | -3.13 | 11.15 |  |  |
|  | 2012E2 | CGR5808 | HAU0423 |  |  |  |  |  |  |  |  | 3.94 | | -3.59 | 9.09 |  |  |
| *qLY-Chr1-1* | 2015E2 | NAU3177 | ICR03724 | 2.58 | -1.27 | 5.44 |  |  |  |  |  |  | |  |  |  | A |
|  | 2016E2 | NAU3177 | ICR03724 |  |  |  |  |  |  |  |  | 3.37 | | -1.67 | 7.30 |  |  |
|  | 2012E2 | NAU3177 | SWU10987 |  |  |  |  |  |  |  |  | 4.21 | | -1.54 | 10.08 |  |  |
|  | 2012E3 | NAU3177 | SWU10987 | 3.21 | -3.31 | 8.48 |  |  | (±1.21) |  |  | 3.35 | | -2.06 | 7.02 | 0.6 | PD |
| *qLY-Chr2-1* | 2015E1 | SWU12025 | SWU11889 |  |  |  |  |  |  |  |  | 4.72 | | 4.11 | 19.42 |  |  |
|  | 2015E2 | SWU11887 | SWU11976 |  |  |  |  |  |  |  |  | 3.83 | | 1.66 | 7.75 |  |  |
|  | 2015E2 | SWU11887 | SWU11976 |  |  |  |  |  |  |  |  | 3.66 | | 1.90 | 8.85 |  |  |
|  | 2016E1 | SWU11889 | SWU11950 | 6.68 | 2.23 | 19.61 |  | 5.15 | 1.56 | 12.85 |  |  | | (±0.68) |  | 2.3 | OD |
|  | 2016E2 | SWU11887 | SWU11976 | 3.20 | 1.42 | 7.81 |  | 2.86 | 1.18 | 8.03 |  |  | | (±0.24) |  | 4.9 | OD |
|  | 2012E3 | SWU12025 | SWU11889 |  |  |  |  |  |  |  |  | 8.48 | | 3.29 | 18.50 |  |  |
|  | 2012E3 | SWU11889 | SWU11887 |  |  |  |  |  |  |  |  | 10.30 | | 3.29 | 19.45 |  |  |
|  | 2012E3 | SWU11887 | SWU11976 | 3.42 | 3.27 | 8.05 |  |  | (±0.30) |  |  | 8.61 | | 3.57 | 21.91 | 0.1 | PD |
|  | 2012E1 | SWU11887 | SWU11976 |  |  |  |  |  |  |  |  | 3.96 | | 1.36 | 8.59 |  |  |
| *qLY-Chr20-1* | 2015E2 | SWU20501a | CER0167 |  |  |  |  |  |  |  |  | 3.48 | | -1.65 | 6.70 |  |  |
|  | 2012E2 | CER0167 | SWU20064 |  |  |  |  |  |  |  |  | 3.76 | | -1.79 | 14.22 |  |  |
| *qLY-Chr21-2* | 2015E1 | HAU0423 | CGR5806 |  |  |  |  | 3.72 | 1.03 | 8.02 |  |  | |  |  |  | OD |
|  | 2015E3 | HAU0423 | CGR5806 |  |  |  |  |  |  |  |  | 4.92 | | -1.78 | 9.82 |  |  |
|  | 2016E1 | CGR5806 | DPL0777 |  |  |  |  |  |  |  |  | 3.65 | | -2.40 | 16.15 |  |  |
|  | 2016E2 | CGR5806 | DPL0777 |  |  |  |  |  |  |  |  | 3.29 | | -2.53 | 16.66 |  |  |
|  | 2012E1 | CGR5808 | HAU0423 |  |  |  |  |  |  |  |  | 3.04 | | -1.03 | 8.12 |  |  |
| *qBNP-Chr1-2* | 2015E2 | NAU3177 | ICR03724 | 3.77 | -0.69 | 7.97 |  |  |  |  |  |  | |  |  |  | A |
|  | 2012E2 | SWU10986 | NAU2218 |  |  |  |  |  |  |  |  | 3.24 | | -0.80 | 7.29 |  |  |
|  | 2012E3 | NAU3177 | SWU10987 |  |  |  |  |  |  |  |  | 2.80 | | -0.97 | 7.05 |  |  |
| *qBNP-Chr21-2* | 2015E1 | CGR5808 | HAU0423 | 2.95 | 0.62 | 8.88 |  |  |  |  |  |  | |  |  |  | A |
|  | 2016E2 | HAU0423 | CGR5806 | 4.27 | -1.02 | 12.22 |  |  |  |  |  |  | |  |  |  | A |
|  | 2012E1 | CGR5808 | HAU0423 |  |  |  |  |  |  |  |  | 3.21 | | -0.68 | 6.27 |  |  |
| *qBNP-Chr24-1* | 2015E3 | Gh268 | SWU13268 |  |  |  |  |  |  |  |  | 4.28 | | 0.73 | 9.11 |  |  |
|  | 2012E3 | Gh268 | SWU13268 | 3.02 | 1.36 | 6.17 |  |  | (±0.31) |  |  |  | |  |  | 0.3 | PD |
|  | 2012E3 | PGML4657 | Gh454 |  |  |  |  |  |  |  |  | 3.88 | | 1.05 | 8.57 |  |  |
| *qBW-Chr2-1* | 2015E2 | SWU11889 | SWU11887 |  |  |  |  |  |  |  |  | 2.54 | | 0.13 | 7.12 |  |  |
|  | 2016E2 | SWU11889 | SWU11887 | 4.68 | 0.11 | 11.22 |  |  |  |  |  |  | |  |  |  | A |
|  | 2012E1 | SWU11889 | SWU11887 |  |  |  |  |  |  |  |  | 2.05 | | 0.10 | 3.74 |  |  |
|  | 2012E3 | SWU11976 | SWU11950 |  |  |  |  |  |  |  |  | 2.29 | | 0.11 | 4.34 |  |  |
|  | 2012E2 | SWU11887 | SWU11976 |  |  |  |  |  |  |  |  | 3.81 | | 0.14 | 10.34 |  |  |
| *qBW-Chr5-1* | 2015E1 | SWU20913 | Gh260 | 4.83 | 0.13 | 13.51 |  |  |  |  |  |  | |  |  |  | A |
|  | 2015E3 | PGML0120 | SWU20914 | 3.73 | 0.08 | 7.60 |  |  |  |  |  |  | |  |  |  | A |
|  | 2016E1 | SWU20913 | Gh260 | 5.24 | 0.13 | 10.95 |  | 2.67 | 0.10 | 6.09 |  |  | | (±0.03) |  | 3.6 | OD |
|  | 2012E2 | SWU20913 | Gh260 |  |  |  |  |  |  |  |  | 3.53 | | 0.13 | 8.94 |  |  |
|  | 2012E3 | PGML0120 | SWU20914 |  |  |  |  |  |  |  |  | 6.01 | | 0.15 | 11.56 |  |  |
| *qBW-Chr5-2* | 2015E2 | SWU20917 | NAU6240 |  |  |  |  |  |  |  |  | 2.63 | | 0.13 | 7.18 |  |  |
|  | 2015E3 | SWU20917 | NAU6240 | 4.00 | 0.10 | 12.34 |  |  | (±0.04) |  |  | 3.68 | | 0.14 | 11.38 | 0.3 | PD |
|  | 2016E2 | SWU20917 | NAU6240 | 3.93 | 0.11 | 11.11 |  |  | (±0.02) |  |  | 4.61 | | 0.15 | 13.31 | 0.2 | PD |
|  | 2016E2 | SWU20917 | NAU6240 |  |  |  |  |  |  |  |  | 3.66 | | 0.13 | 9.06 |  |  |
|  | 2012E2 | SWU20917 | NAU6240 |  |  |  |  |  |  |  |  | 6.28 | | 0.17 | 15.73 |  |  |
|  | 2012E3 | SWU20917 | NAU6240 | 3.60 | 0.17 | 9.85 |  |  | (±0.00) |  |  | 6.81 | | 0.21 | 18.19 |  | PD |
| *qBW-Chr23-1* | 2015E2 | SWU14807 | PGML4185 | 2.88 | 0.08 | 5.60 |  |  |  |  |  |  | |  |  |  | A |
|  | 2015E2 | PGML4186 | NAU3100 | 2.52 | 0.08 | 5.61 |  | 2.54 | 0.08 | 5.42 |  |  | |  |  | 16.0 | OD |
|  | 2012E1 | SWU14807 | PGML4185 |  |  |  |  |  |  |  |  | 2.14 | | 0.11 | 4.48 |  |  |
|  | 2012E2 | SWU14807 | PGML4185 |  |  |  |  |  |  |  |  | 3.10 | | 0.11 | 6.00 |  |  |
| *qLP-Chr5-1* | 2015E2 | SWU20913 | Gh260 | 6.57 | -0.50 | 13.75 |  |  | (±0.16) |  |  | 5.19 | | -0.66 | 9.73 | 0.2 | PD |
|  | 2015E3 | SWU20913 | Gh260 | 7.59 | -0.59 | 15.76 |  |  |  |  |  |  | |  |  |  | A |
|  | 2016E1 | PGML0120 | SWU20914 | 7.33 | -0.67 | 14.23 |  |  |  |  |  |  | |  |  |  | A |
|  | 2012E1 | SWU20913 | Gh260 | 6.07 | -1.05 | 14.30 |  |  | (±0.01) |  |  | 10.70 | | -1.06 | 21.13 |  | PD |
|  | 2012E3 | SWU20913 | Gh260 | 6.36 | -0.89 | 13.67 |  |  | (±0.18) |  |  | 8.03 | | -1.07 | 19.78 | 0.2 | PD |
|  | 2012E2 | SWU20913 | Gh260 |  |  |  |  |  |  |  |  | 11.30 | | -1.01 | 25.28 |  |  |
|  | 2012E3 | PGML0120 | SWU20914 |  |  |  |  |  |  |  |  | 12.80 | | -1.13 | 23.26 |  |  |
| *qLP-Chr5-2* | 2015E1 | SWU20917 | NAU6240 | 5.00 | -0.60 | 14.15 |  |  | (±0.77) |  |  | 4.03 | | 0.17 | 11.27 | 4.5 | OD |
|  | 2015E2 | SWU20917 | NAU6240 |  |  |  |  |  |  |  |  | 5.58 | | -0.80 | 14.22 |  |  |
|  | 2015E2 | SWU20917 | NAU6240 | 5.88 | -0.60 | 15.42 |  |  |  |  |  | 7.59 | | -0.99 | 18.75 | 0.4 | PD |
|  | 2015E3 | SWU20917 | NAU6240 | 8.04 | -0.71 | 22.38 |  |  |  |  |  | 9.06 | | -1.03 | 26.92 | 0.3 | PD |
|  | 2016E1 | PGML1671 | PGML1917 | 7.75 | -0.81 | 20.82 |  | 2.62 | -0.28 | 5.18 |  | 7.36 | | -1.13 | 18.80 | 0.5 | PD |
|  | 2016E2 | SWU20917 | NAU6240 | 5.16 | -0.52 | 10.78 |  |  | (±0.54) |  |  | 7.45 | | -1.06 | 20.01 | 0.5 | PD |
|  | 2016E2 | SWU20917 | NAU6240 | 7.78 | -0.69 | 19.50 |  |  | (±0.35) |  |  | 9.73 | | -1.04 | 20.51 | 0.3 | PD |
|  | 2012E1 | SWU20917 | NAU6240 | 6.11 | -1.09 | 15.54 |  |  | (±0.13) |  |  | 10.50 | | -1.22 | 28.20 | 0.1 | PD |
|  | 2012E2 | SWU20917 | NAU6240 | 6.26 | -1.24 | 16.93 |  |  | (±0.21) |  |  | 10.70 | | -1.03 | 26.16 | 0.2 | PD |
|  | 2012E3 | SWU20917 | NAU6240 |  |  |  |  |  | 0.61 | 9.88 |  | 14.80 | | -1.41 | 37.72 | 0.5 | PD |
| *qLP-Chr13-2* | 2015E3 | NAU2893 | Gh157 |  |  |  |  |  |  |  |  | 3.73 | | -0.57 | 7.89 |  |  |
|  | 2016E1 | NAU2893 | Gh157 | 2.67 | -0.41 | 4.98 |  |  |  |  |  |  | |  |  |  | A |
|  | 2016E2 | NAU2893 | Gh157 |  |  |  |  |  |  |  |  | 2.68 | | -0.57 | 5.38 |  |  |
|  | 2012E2 | DPL0572 | HAU2558 |  |  |  |  |  | -0.56 | 6.73 |  |  | |  |  |  | OD |
|  | 2012E3 | Gh157 | BNL1495 | 3.57 | -0.64 | 6.75 |  |  | (±0.13) |  |  | 5.28 | | -0.68 | 8.61 | 0.3 | PD |
|  | 2012E3 | DPL0572 | HAU2558 |  |  |  |  |  |  |  |  | 2.22 | | -0.51 | 4.41 |  |  |
| *qLP-Chr13-3* | 2015E2 | BNL1495 | CGR5390 |  |  |  |  | 4.75 | 0.46 | 13.26 |  | 4.79 | | -0.72 | 11.27 | 0.6 | PD |
|  | 2015E2 | BNL1495 | CGR5390 |  |  |  |  |  |  |  |  | 3.54 | | -0.66 | 8.21 |  |  |
|  | 2015E3 | BNL1495 | CGR5390 |  |  |  |  |  |  |  |  | 2.99 | | -0.54 | 7.05 |  |  |
|  | 2016E1 | BNL1495 | CGR5390 |  |  |  |  |  |  |  |  | 6.21 | | -0.95 | 13.25 |  |  |
|  | 2016E2 | BNL1495 | CGR5390 |  |  |  |  |  |  |  |  | 3.00 | | -0.61 | 6.44 |  |  |
|  | 2016E2 | DPL0894 | SWU10800 | 5.17 | -0.65 | 17.59 |  |  | (±0.04) |  |  |  | | (-0.61) |  | 0.1 | PD |
|  | 2012E1 | DPL0894 | SWU10800 | 2.04 | -0.69 | 5.61 |  |  |  |  |  |  | |  |  |  | A |
|  | 2012E3 | BNL1495 | CGR5390 | 2.90 | -0.65 | 7.11 |  |  | (±0.08) |  |  | 4.10 | | -0.73 | 9.13 | 0.1 | PD |
|  | 2012E1 | DPL0894 | SWU10800 |  |  |  |  |  |  |  |  | 3.17 | | -0.72 | 9.98 |  |  |
| *qLP-Chr19-1* | 2015E2 | NAU3437 | NAU2894 | 2.88 | 0.37 | 5.68 |  |  |  |  |  |  | |  |  |  | A |
|  | 2016E1 | NAU3437 | NAU2894 |  |  |  |  |  |  |  |  | 2.69 | | 1.03 | 15.67 |  |  |
|  | 2016E2 | NAU3437 | NAU2894 |  |  |  |  |  |  |  |  | 3.00 | | 0.65 | 8.11 |  |  |
|  | 2012E1 | NAU3437 | NAU2894 | 3.19 | 0.69 | 6.18 |  |  | (±0.12) |  |  | 4.05 | | 0.57 | 6.80 | 0.2 | PD |
|  | 2012E2 | NAU3437 | NAU2894 | 4.02 | 1.02 | 11.37 |  |  | (±0.46) |  |  | 3.57 | | 0.56 | 6.48 | 0.8 | PD |

**Supplementary Table S10. Common single locus QTLs in previous study in 2008 and 2009 in comparison to QTLs in present study.** See footnotes in Table S2.

| **QTLs** | **Population** | **Env.** | **Chr.** | **Flanking marker** | | **LOD** | **A** | **D** | ***d/a*** | **Var%** |
| --- | --- | --- | --- | --- | --- | --- | --- | --- | --- | --- |
| *qBNP-Chr19-1* | F_2:3_ | 2008E1 | 19 | NAU3437 | GH616 | 2.04 | -1.00 | 1.10 | 1.15 | 8.21 |
|  | F_2:4_ | 2009E2 | 19 | NAU3437 | GH616 | 2.43 | -0.90 | 0.97 | 1.14 | 20.23 |
| *qBNP-Chr11-1* | F_2:4_ | 2009E2 | 11 | CGR5808 | CGR5217 | 3.03 | 1.46 | -1.20 | -0.79 | 8.79 |
| *qBNP-Chr22-1* | F_2:4_ | 2009E1 | 22 | CGR5806 | CGR6410 | 3.26 | -0.20 | 1.07 | 5.11 | 6.96 |
| *qBW-Chr5-1* | F_2:3_ | 2008E1 | 5 | BNL3447 | GH260 | 2.40 | -0.20 | 0.17 | 0.71 | 7.54 |
|  | F_2:3_ | 2008E2 | 5 | BNL3447 | GH260 | 2.50 | -0.20 | -0.01 | -0.06 | 8.14 |
|  | F_2:4_ | 2009E2 | 5 | BNL3447 | GH260 | 2.63 | -0.10 | -0.01 | -0.29 | 7.20 |
| *qBW-Chr5-2* | F_2:4_ | 2009E1 | 5 | GH388 | HAU1603 | 2.05 | -0.10 | -0.01 | -0.10 | 3.84 |
|  | F_2:3_ | 2008E2 | 5 | NAU2865 | GH388 | 4.61 | -0.30 | -0.10 | -0.35 | 9.69 |
|  | F_2:3_ | 2008E1 | 5 | HAU1603 | TMB1296 | 2.70 | -0.20 | 0.07 | 0.30 | 5.94 |
| *qBW-Chr6-1* | F_2:3_ | 2008E1 | 6 | DPL124 | HAU1460 | 2.90 | 0.20 | -0.01 | -0.21 | 7.05 |
|  | F_2:4_ | 2009E1 | 6 | CGR6749 | DPL124 | 2.69 | 0.06 | 0.07 | 1.23 | 5.13 |
| *qBW-Chr11-1* | F_2:4_ | 2009E1 | 11 | BNL3171 | CGR5808 | 6.70 | 0.23 | -0.01 | -0.18 | 14.11 |
|  | F_2:4_ | 2009E2 | 11 | DPL050a | HAU423 | 2.68 | 0.21 | -0.20 | -0.85 | 6.68 |
| *qBW-Chr11-2* | F_2:4_ | 2009E2 | 11 | CGR5808 | CGR5217 | 3.74 | 0.23 | -0.10 | -0.61 | 10.44 |
| *qLP-Chr5-1* | F_2:3_ | 2008E1 | 5 | GH260 | NAU6240 | 3.76 | 0.95 | -0.80 | -0.81 | 9.33 |
|  | F_2:3_ | 2008E2 | 5 | GH260 | NAU6240 | 6.10 | 1.34 | -0.80 | -0.56 | 16.70 |
|  | F_2:4_ | 2009E2 | 5 | GH260 | NAU6240 | 6.68 | 1.20 | -0.60 | -0.47 | 16.18 |
